# Comprehensive metabolomic characterization of atrial fibrillation

**DOI:** 10.3389/fcvm.2022.911845

**Published:** 2022-08-08

**Authors:** Chengcan Lu, Chunyan Liu, Di Mei, Mengjie Yu, Jian Bai, Xue Bao, Min Wang, Kejia Fu, Xin Yi, Weihong Ge, Jizhong Shen, Yuzhu Peng, Wei Xu

**Affiliations:** ^1^Nanjing Drum Tower Hospital, China Pharmaceutical University, Nanjing, China; ^2^Department of Pharmacy, Nanjing Drum Tower Hospital, The Affiliated Hospital of Nanjing University Medical School, Nanjing, China; ^3^Department of Cardiology, Nanjing Drum Tower Hospital, The Affiliated Hospital of Nanjing University Medical School, Nanjing, China; ^4^Key Laboratory of Drug Metabolism and Pharmacokinetics, China Pharmaceutical University, Nanjing, China

**Keywords:** atrial fibrillation, metabolomics, diagnostic model, risk factors, biomarker

## Abstract

**Background:**

Using human humoral metabolomic profiling, we can discover the diagnostic biomarkers and pathogenesis of disease. The specific characterization of atrial fibrillation (AF) subtypes with metabolomics may facilitate effective and targeted treatment, especially in early stages.

**Objectives:**

By investigating disturbed metabolic pathways, we could evaluate the diagnostic value of biomarkers based on metabolomics for different types of AF.

**Methods:**

A cohort of 363 patients was enrolled and divided into a discovery and validation set. Patients underwent an electrocardiogram (ECG) for suspected AF. Groups were divided as follows: healthy individuals (Control), suspected AF (Sus-AF), first diagnosed AF (Fir-AF), paroxysmal AF (Par-AF), persistent AF (Per-AF), and AF causing a cardiogenic ischemic stroke (Car-AF). Serum metabolomic profiles were determined by gas chromatography–mass spectrometry (GC-MS) and liquid chromatography–quadrupole time-of-flight mass spectrometry (LC-QTOF-MS). Metabolomic variables were analyzed with clinical information to identify relevant diagnostic biomarkers.

**Results:**

The metabolic disorders were characterized by 16 cross-comparisons. We focused on comparing all of the types of AF (All-AFs) plus Car-AF vs. Control, All-AFs vs. Car-AF, Par-AF vs. Control, and Par-AF vs. Per-AF. Then, 117 and 94 metabolites were identified by GC/MS and LC-QTOF-MS, respectively. The essential altered metabolic pathways during AF progression included D-glutamine and D-glutamate metabolism, glycerophospholipid metabolism, etc. For differential diagnosis, the area under the curve (AUC) of specific metabolomic biomarkers ranged from 0.8237 to 0.9890 during the discovery phase, and the predictive values in the validation cohort were 78.8–90.2%.

**Conclusions:**

Serum metabolomics is a powerful way to identify metabolic disturbances. Differences in small–molecule metabolites may serve as biomarkers for AF onset, progression, and differential diagnosis.

## Introduction

In clinical practice globally, atrial fibrillation (AF) is the most common cardiac arrhythmia, with substantial morbidity and mortality (1). The prevalence of AF in adults is currently estimated at 2–4% (2). As a result of extended longevity in the general population and the intensified search for undiagnosed AF, the incidence of AF is expected to increase 2.3-fold (3, 4). Among the elderly (aged >85 years), the prevalence is expected to rise to 17.8% (5). AF not only causes clinical symptoms (6), but it also raises the risk of stroke, heart failure, hospitalization, and cardiac death (7), thus severely burdening patients, society, and the economy (8).

It is well-known that AF is one of the most critical risk factors for ischemic stroke (9). When AF causes a cardioembolic stroke, it is usually severe, highly recurrent, and often fatal (10–12). By deciphering the metabolic mechanisms of AF, the incidence and mortality could be reduced. In addition, the embolus formed in the left atrial appendage, a common reason for cardiogenic ischemic stroke caused by AF, is a slow and complex process (13). However, the exact causes of AF, how it develops over time, and how a blood clot enters the cerebral vessels are unknown.

By screening and diagnosing AF early, optimal treatments can be initiated for patients. Current screening tools for AF are largely based on patient symptoms (14), electrophysiological examination (15), electrocardiogram (ECG), and some mobile health technologies (16). Among these methods, the 12-lead ECG has been the “gold standard” for arrhythmia diagnosis for over a century (17). However, a baseline resting ECG may be insufficient for diagnosing AF, as it can be paroxysmal and asymptomatic (18, 19). In addition, the sensitivity, specificity, and technology of other screening tools are not yet sufficient or mature.

The metabolome of biofluids changes because of metabolic changes in the heart (20). Metabolites of multiple small molecules may provide excellent diagnostic information (21). The field of metabolomics is rapidly growing in systems biology and includes the study of metabolic alterations caused by disease (22). As an essential source for metabolic profiling, the serum is not interfered with by anticoagulants (23). A single analytical platform cannot detect all of the metabolites in a biological sample because of the complexity of mammalian metabolomes (24). The application of metabolomics in clinical epidemiology needs to adjust for clinical confounding factors (25). Without the validation set, the discovery set can also lead to an unstable prediction model (26). Therefore, we used the dual platforms of gas chromatography–mass spectrometry (GC-MS) and liquid chromatography–quadrupole time-of-flight mass spectrometry (LC-QTOF-MS) to discover and validate the diagnostic model of AF, combining individual differences. We also performed a comprehensive metabolomic evaluation to identify the difference among AF subtypes.

## Materials and methods

Participants from the center (Nanjing Drum Tower Hospital, The Affiliated Hospital of Nanjing University Medical School, Nanjing, Jiangsu, China) formed the discovery and validation cohorts between February 2020 and June 2021. The ethics committee approved this study, which was completed under the guidance of the Helsinki Declaration (Lot number: 2020346).

### Inclusion criteria

Inclusion criteria were symptoms of palpitations, fatigue, dizziness, dyspnea, chest pain, anxiety, cardiovascular risk factors, or abnormal changes in cardiac biomarkers, such as cardiac troponin (cTn), B-type natriuretic peptide (BNP), and myocardial enzymes. The diagnosis could be confirmed with an ECG. Exclusion criteria were: severe heart valve diseases, heart failure with left ventricular ejection fraction <20%, and acute coronary syndrome (ACS). Patients with acute myocarditis, pericarditis, Takotsubo cardiomyopathy, aortic dissection, pulmonary embolism, malignant tumor, autoimmune disorders, trauma, or a recent surgical procedure were excluded. In addition, we excluded patients with liver dysfunction (alanine aminotransferase level >135 U/l), severe renal dysfunction (creatinine >3.0 mg/dl), or blood-borne infectious diseases, including human immunodeficiency virus/acquired immunodeficiency syndrome, hepatitis B, and hepatitis C.

### Definition of different groups

As a supraventricular tachyarrhythmia, AF is marked by uncoordinated atrial electrical activity and inefficient atrial contraction. AF's ECG characteristics include irregular R-R intervals, the absence of distinct repeating P waves, and irregular atrial activations (27). The minimum duration of an ECG tracing of AF required to establish the diagnosis of clinical AF is at least 30 s (28). First diagnosed AF (Fir-AF) refers to AF that has not been diagnosed before, irrespective of its duration or the presence/severity of AF-related symptoms. Paroxysmal AF (Par-AF) refers to AF that terminates spontaneously or with intervention within 7 days of onset. Persistent AF (Per-AF) is defined as AF that is continuously sustained beyond 7 days, including episodes terminated by cardioversion after ≥7 days (27). Long-term persistence and Permanent AF are more representatives of the treatment attitude of patients and physicians. Suspected AF (Sus-AF) is some cardiovascular disease with similar symptoms but different ECG manifestations, such as premature beats, ventricular tachycardia, etc. Cardiogenic ischemic stroke caused by AF is called Car-AF. The ECG data were confirmed independently by two senior physicians in each study.

### Sample collection

For all of the enrolled participants, serum samples were collected on the next day of hospitalization in the Cardiovascular Inpatient Ward and on the day of the medical examination in the Health Management Center. In the morning, procoagulant tubes were used to collect venous blood samples from fasting individuals. After 2 h, we transferred each supernatant serum to another tube and froze it at −80°C in a refrigerator. We thawed the samples at room temperature for 20 min and vortexed and centrifuged them at 650 g for 3 min before use. Pooled quality control samples were prepared by mixing equal amounts of serum from every enrolled individual to ensure data quality (24).

### Chemicals

L-2-chlorophenyl alanine (L-Cl-Phe) was purchased from PepTech Corporation (Burlington, Massachusetts, USA). We obtained pyridine (≥99.8% GC), methoxamine hydrochloride (purity 98%), methyl myristate, and (^13^C2)-myristic acid From Sigma-Aldrich (St. Louis, MO, USA). The reagents were provided by Pierce Chemical (Rockford, IL, USA) as N-methyl-trimethylsilyl-trifluoroacetamide (MSTFA) and 1% v/v trimethylchlorosilane (TMCS). The HPLC grades of acetonitrile, methanol, and n-heptane were purchased from Merck (Darmstadt, Germany). The Milli-Q (Millipore, Bedford, MA, USA) system produced purified water. Aladdin (Shanghai, China) supplied the formic acid.

### Sample preparation

The samples were prepared according to a previously reported method (29). First, we added 50 μl of serum to 200 μl of methanol [containing internal standard (IS), 5 μg/ml (^13^C2)-myristic acid] and then vortexed vigorously for 5 min. After that, we extracted the samples at 1,800 rpm for 10 min in the SORVALL Biofuge Stratos centrifuge (Sollentum, Germany). After transferring 180 μl of supernatant into another tube, we centrifuged again for 10 min, transferred 100 μl of supernatant into a GC vial, and evaporated it to dryness in a SpeedVac concentrator (Thermo Fisher Scientific, SavantTM SC250EXP, Holbrook, USA). Next, we added 30 μl of methoxyamine pyridine solution (10 mg/ml) to the residue and incubated it for 16 h at room temperature. We trimethylsilylated the analytes by adding 30 μl of MSTFA and TMCS as a catalyst. After 1 h, we used an external standard (ES), 30 μl of n-heptane containing methyl myristate (15 μg/ml), to monitor the stability of the instruments. The final 90–μl mixture was vortexed for 1 min and then ready for GC/MS analysis. For LC-QTOF-MS analysis, we slightly modified the pretreating method. Unlike the method mentioned above, we used L-Cl-Phe dissolved in methanol (15 μg/ml) as IS. After evaporation, we redissolved the residue with 100 μl of purified water. Finally, we centrifuged the dissolved solution at 18,000 rpm for 5 min and transferred 80 μl of supernatant to an LC vial.

### Metabolomics study

Dual metabolomics platforms were used in the discovery set following previously developed methods (30). The LC-QTOF-MS analysis was carried out in the validation set as previously reported (31). Random numbers were generated in Excel (Microsoft, Redmond, Washington) to assign samples. Then, 10 blank samples were injected first during analyses of the sample sequence to ensure a stable baseline. One quality control sample was run after every 15 injections of the prepared sera. One blank sample sequence was run after dozens of random injections during analyses of the sample sequence in LC-QTOF-MS. After the complete run, we performed tasks including chromatogram acquisition, retention time alignment, peak deconvolution, metabolite identification, integration of chromatograms, etc., to pre-process the data. The “80% rule” (32) and “20% RSD rule” (33) were used to clean data for each sample group. The specific parameters are shown in Table 1.

**Table 1:** Specific parameters for metabolomic studies.

	**GC/MS**	**LC-QTOF-MS 1**	**LC-QTOF-MS 2**
Instruments	Shimadzu GCMS-QP2010 (Ultra, Kyoto, Japan)	ACQUITY UPLC H-Class System, Xevo G2-XS-Quadrupole-Time of Flight system (Waters Corporation, USA)	Exion LC AD System, TripleTOF^®^5,600-Quadrupole/Time-of-Flight system (AB SCIEX LLC., Redwood City, CA, USA).
Columns	Rtx-5 MS capillary column (0.25 μm, 0.25 mm × 30 m; Restek, PA, USA)	Amide XBridge UPLC column (3.5 μm, 4.6 mm × 100 mm; Waters Corporation, USA)	Kinetex C18 column (2.6 μm, 100 mm × 2.1 mm; Phenomenex, Torrance, CA, USA)
Injection volume	0.5 μl	10 μl	10 μl
Chromatographic conditions	Split mode (split ratio 8:1); injector temperature 250°C; septum purge flow rate 6 ml/min; carrier gas flow rate 1.5 ml/min. Column temperature gradient: 0–5 min, 80°C; 5–16 min, 80°C−300°C; 16–21 min, 300°C	Flow rate 0.4 ml/min; column temperature 40°C; autosampler temperature 4°C. Mobile phase: 0.1% formic acid (solvent A), acetonitrile (solvent B). Solvent gradient: 0–3 min, 85% B; 3–6 min, 85–30% B; 6–15 min, 30–2% B; 15–18 min, 2% B; 18–19 min, 2–85% B; and 19–26 min, 85% B	Flow rate 0.4 ml/min; column temperature 40°C; autosampler temperature 4°V. Mobile phase: 0.1% formic acid (solvent A), acetonitrile (solvent B). Solvent gradient: 0–1 min, 10–30% B; 1–19 min, 30–95% B; 19–20 min, 95% B
Mass spectrometry conditions	Electron impact (EI) ion source; ion source temperature 220°C; ionization electron beam 70 eV; full scan mode, m/z 50–700; run time 19 min, solvent cutting acquisition time 4.5 min	Electrospray ionization (ESI) ion source; positive ion mode, gas 1 pressure 50 psi, gas 2 pressure 30 psi, curtain gas 30 psi; ion spray voltage 4,500 V; turbo spray temperature 500°C; full scan m/z 50–1,000, product ions m/z 50–900; declustering potential (DP) 100 V, collision energy (CE) 35 eV	Electrospray ionization (ESI) ion source; positive and negative ion modes; information-dependent acquisition (IDA) criteria; ion spray voltage +5,500/−4,500 V; turbo spray temperature 550°C; nebulizer gas pressure 55 psi; heater gas pressure 55 psi; curtain gas pressure 35 psi; DP ±80 V; CE 35 eV; mass range m/z 60–1,000
Data pre-processing software	Shimadzu GC Postrun Analysis	Progenesis QI (Waters Corporation, USA)	Analysis Base File Converter, MSDIAL ver.4.70
Metabolite identification	National Institute of Standards and Technology (NIST) library 2.0 (2008); Wiley 9 (Wiley-VCH Verlag GmbH & Co. S5 KGaA, Weinheim, Germany); Standard compound self-built library (CPU library, China Pharmaceutical University, China)	Human Metabolome Database (HMDB), METLIN, Chemspider, CPU library	MSMS-Public-Pos-VS15, MSMS-Public-Neg-VS15 (matching MSDIAL), CPU library
Normalization method	IS normalization	IS normalization	LOWESS regression

### Statistical analysis

Continuous variables with normal distribution are presented as mean ± standard deviation, and those with skewed distribution are presented as median (lower quartile, upper quartile). Differences between two groups and among three or more groups were compared using the Student's *t*-test and one-way ANOVA, respectively, for normally-distributed continuous variables, or the Mann–Whitney *U*-test and Kruskal Wallis *H*-test for skewed variables. Categorical variables are described as numbers and percentages, and the differences across groups were measured by the chi-square test or Fisher's exact test where appropriate. The Benjamini–Hochberg false discovery rate (FDR) correction adjusted all of the *P*-values, and an adjusted *P*-value of < 0.05 was considered to be statistically significant.

The GC/MS and LC/MS semiquantitative data were logarized and distributed normally. The differential metabolites that satisfied the criterion variable importance in the projection (VIP) of >1.0 and P (independent sample *t*-test) of <0.05 were considered to be biomarker candidates. We used normalized data to calculate fold-change (FC) values. We also built and plotted the clustering or separation of samples from different groups by principal component analysis (PCA), partial least square to latent structure discriminant analysis (PLS-DA), and orthogonal partial least squares discriminant analysis (OPLS-DA) models. We selected the appropriate metabolites as variables for binary logistic regression analysis and then chose the markers with the best diagnostic efficacy to establish a prediction model. The diagnostic performance of the selected model was evaluated using the value of areas under the curve (AUC) by receiver operating characteristic (ROC) curve analysis (34). According to the Youden Index, the appropriate sensitivity and specificity were chosen (35). SIMCA-P 14.1 (Umetrics, Umeå, Sweden) and SPSS (version 26.0, SPSS Inc., Chicago, IL, USA) were used to screen and choose the discriminant metabolites between different groups. Metabolic pathway analysis and heatmaps cluster analysis were performed using online MetaboAnalyst 5.0 (https://www.metaboanalyst.ca/). We also used SPSS to obtain logistic regression analysis and ROC analysis. Other column and scatter plots were made by GraphPad Prism 8.0.

## Results

### Clinical descriptions

**Figure 1:**
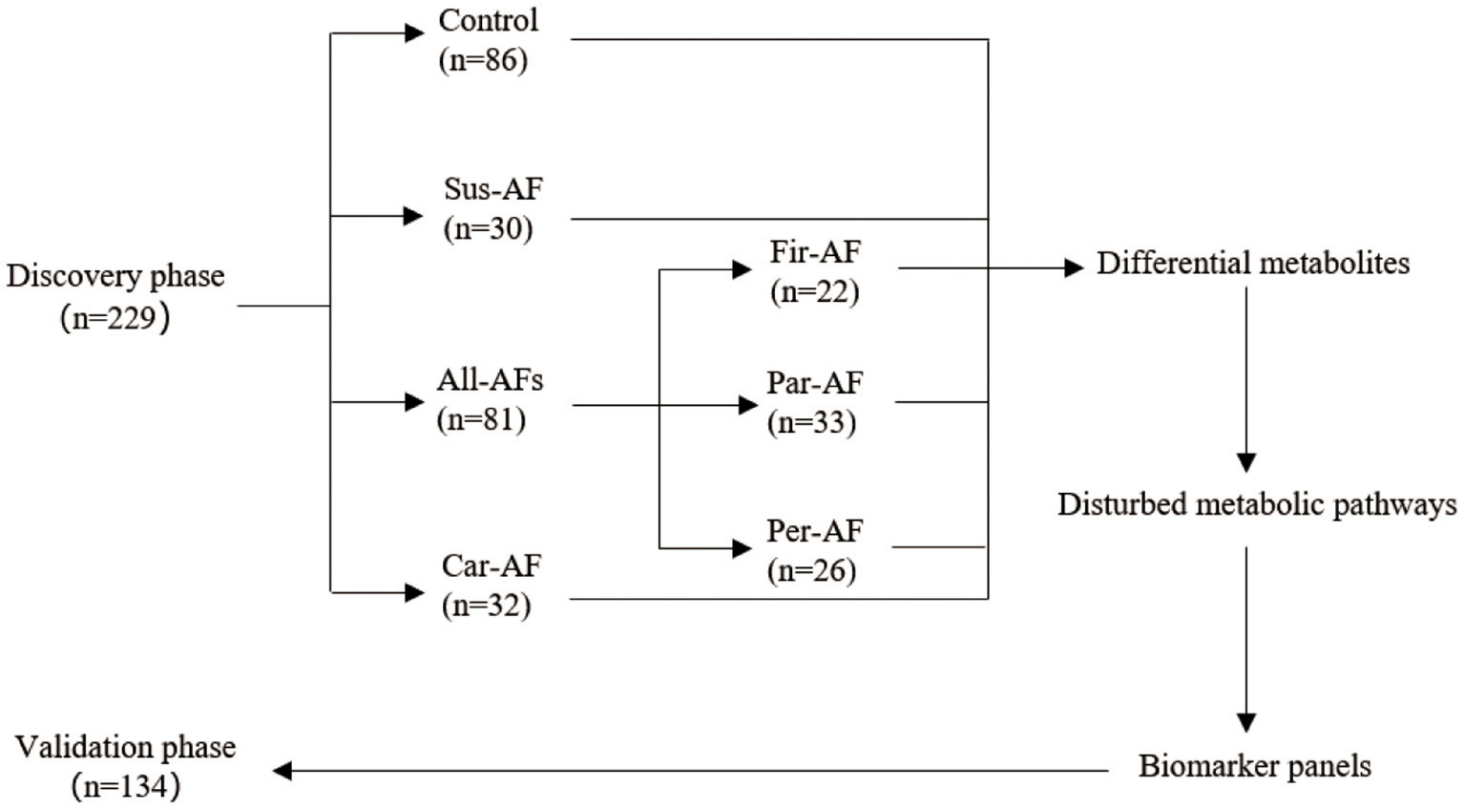
Study design.

A total of 363 participants were enrolled (Figure 1). The discovery phase enrolled 229 volunteers, namely, 86 Control, 30 Sus-AF, 32 Car-AF, and 81 All-AFs (22 Fir-AF, 33 Par-AF, and 26 Per-AF) cases. We present baseline characteristics and laboratory data in Table 2 and Supplementary Tables 1, 2. The validation phase included 134 participants, namely, 30 Control, 17 Fir-AF, 36 Par-AF, 29 Per-AF, and 22 Car-AF cases. Their baseline characteristics are shown in Supplementary Tables 3, 4.

**Table 2:** Baseline characteristics of discovery phase participants.

	**Variable**	**Controls** **(*n* = 86)**	**Sus-AF** **(*n* = 30)**	**All-AFs plus Car-AF** **(*n* = 113)**	***P***
Demographics	Male	38 (43.7)	14 (46.7)	73 (64.6)	–
	Age	57.47 ± 6.58	54.83 ± 9.57	65.59 ± 11.66	^*^
	Weight	61.66 ± 8.62	65.68 ± 9.13	69.99 ± 11.91	^*^
	Height	1.65 ± 0.08	1.67 ± 0.08	1.68 ± 0.08	^*^
	BMI	22.62 ± 1.77	23.62 ± 2.36	24.70 ± 3.31	^*^
	BSA	1.76 ± 0.15	1.82 ± 0.15	1.88 ± 0.18	^*^
	SBP	121.01 ± 10.79	128.6 ± 16.36	136.91 ± 20.33	^*^
	DBP	74.15 ± 7.92	81.73 ± 11.74	82.59 ± 12.49	^#*^
	Crcl	91.92 ± 15.33	106.74 ± 31.19	86.96 ± 36.92	^–^
	HbAlc	5.6 (5.3, 5.9)	5.6 (5.4, 5.8)	5.9 (5.5, 6.3)	^*^
Liver function	ALT	14.8 (11.5, 19.4)	19.9 (14.0, 26.0)	16 (13, 22.6)	^#^
	AST	17.8 (15.9, 20.7)	21.35 (17.7, 23.3)	19.1 (15.1, 22.3)	^#^
	ALP	57.1 (48.2, 70.9)	67.45 (56.3, 78.8)	68.9 (58.0, 83.4)	^*^
	γ-GT	18.0 (14.9, 25.8)	25.5 (18.7, 55.3)	25.0 (18.1, 46.8)	^#*^
	LDH	180 (162, 201)	182 (155, 216)	188 (166, 224)	^–^
	TBiL	11.50 ± 4.73	11.93 ± 3.68	13.29 ± 6.91	^–^
	TP	70.42 ± 3.71	65.52 ± 4.78	65.33 ± 5.07	^#*^
	Alb	43.74 ± 1.45	40.65 ± 2.10	39.84 ± 2.93	^#*^
	Glo	26.68 ± 3.27	24.87 ± 3.89	25.49 ± 3.86	^–^
	AG-ratio	1.66 ± 0.21	1.67 ± 0.26	1.60 ± 0.25	^–^
Kidney function	GLU	5.18 (4.81, 5.46)	4.75 (4.51, 5.06)	4.96 (4.56, 5.66)	^#^
	BUN	5.17 ± 1.05	4.97 ± 1.46	5.84 ± 1.75	^*^
	CREA	62.75 ± 11.08	61.70 ± 12.65	68.63 ± 18.67	^*^
	URIC	319.53 ± 59.61	344.10 ± 87.23	359.31 ± 101.75	^*^
	T-CO2	25.60 ± 1.53	24.89 ± 1.25	25.26 ± 2.22	^–^
	eGFR	105.67 ± 16.55	111.97 ± 23.83	102.95 ± 24.17	^–^
Blood lipids	TG	0.94 (0.74, 1.33)	1.29 (1.05, 1.61)	1.14 (0.87, 1.63)	^#*^
	CHOL	4.89 ± 0.75	4.32 ± 0.91	4.31 ± 0.84	^#*^
	HDL	1.57 ± 0.39	1.22 ± 0.44	1.21 ± 0.36	^#*^
	LDL	2.76 ± 0.61	2.45 ± 0.71	2.47 ± 0.73	^*^
White blood	WBC	5.59 ± 1.27	5.65 ± 1.66	6.60 ± 2.43	^*^
cells items	NEUT%	57.84 ± 8.10	60.24 ± 9.36	62.97 ± 12.40	^*^
	LYMPH%	33.71 ± 7.41	30.76 ± 8.68	27.54 ± 10.78	^*^
	MONO%	5.71 ± 1.36	6.34 ± 1.48	6.90 ± 2.17	^*^
	EOS%	2.24 ± 1.36	2.24 ± 1.50	2.13 ± 2.00	^–^
	BASO%	0.50 ± 0.26	0.42 ± 0.19	0.45 ± 0.30	^–^
	NEUT#	3.26 ± 0.98	3.45 ± 1.30	4.36 ± 2.39	^*^
	LYMPH#	1.87 ± 0.52	1.67 ± 0.54	1.65 ± 0.66	^*^
	MONO#	0.32 ± 0.12	0.36 ± 0.15	0.45 ± 0.20	^*^
	EOS#	0.11 (0.06, 0.16)	0.11 (0.05, 0.20)	0.10 (0.05, 0.16)	^–^
	BASO#	0.03 ± 0.01	0.02 ± 0.01	0.03 ± 0.02	^–^
Red blood	RBC	4.62 ± 0.38	4.35 ± 0.40	4.49 ± 0.57	^#^
cells items	HGB	142.15 ± 11.94	134.43 ± 14.65	141.73 ± 17.25	^–^
	HCT	42.54 ± 3.29	39.11 ± 3.48	41.34 ± 4.82	^–^
	MCV	92.26 ± 4.88	90.08 ± 4.31	92.24 ± 4.95	^–^
	MCH	30.83 ± 1.82	30.92 ± 1.94	31.63 ± 1.92	^*^
	MCHC	334.07 ± 6.35	343.23 ± 10.62	342.84 ± 10.96	^#*^
	RDW	12.8 (12.5, 13.2)	12.8 (12.3, 13.2)	12.6 (12.2, 13.1)	^–^
Platelets	PLT	203.40 ± 46.01	208.47 ± 69.77	180.82 ± 59.02	^*^
items	PCT	0.21 ± 0.04	0.21 ± 0.06	0.20 ± 0.05	^–^
	PDW	16.3 (16.0, 16.4)	16.2 (15.7, 16.6)	16.2 (16.0, 16.4)	^–^
	MPV	10.20 ± 1.25	10.75 ± 1.83	10.96 ± 1.40	^*^

# and * indicate significant differences in groups Sus-AF and All-AFs plus Car-AF compared with the Control, respectively. BMI, body mass index; BSA, body surface area; SBP, systolic blood pressure; DBP, diastolic blood pressure; Crcl, creatinine clearance; HbAlc, glycated hemoglobin; ALT, alanine aminotransferase; AST, aspartate aminotransferase; ALP, alkaline phosphatase; γ-GT, γ-glutamyltransferase; LDH, lactate dehydrogenase; TBiL, total bilirubin; TP, total protein; Alb, albumin; Glo, globulin; AG-ratio, the ratio of albumin to globulin; GLU, glucose; BUN, blood urea nitrogen; CREA, Creatinine; UA, uric acid; T-CO2, total carbon dioxide; eGFR, endogenous glomerular filtration rate; TG, triglyceride; CHOL, total cholesterol; HDL, high density lipoprotein; LDL, low density lipoprotein; WBC, number of white blood cells; NEUT%, percentage of neutrophils; LYMPH%, percentage of lymphocytes; MONO%, percentage of monocytes; EOS%, percentage of eosinophils; BASO%, percentage of basophils; NEUT#, number of neutrophils; LYMPH#, number of lymphocytes; MONO#, number of monocytes; EOS#, number of eosinophils; BASO#, number of basophils; RBC, number of red blood cells; HGB, hemoglobin content; HCT, hematocrit; MCV, mean red blood cell volume; MCH, mean red blood cell hemoglobin content; MCHC, mean red blood cell hemoglobin concentration; RDW, red blood cell distribution width; PLT, number of platelet; PCT, platelet hematocrit; PDW, platelet distribution width; MPV, mean platelet cell volume.

Compared to the Control, we found that Age, Weight, Height, BMI, BSA, SBP, HbA1c, AKP, UREA, CREA, URIC, LDLC, WBC, NEUTP, LYMPHP, MONOP, NEUT#, LYMPH#, MONO#, MCH, PLT, and MPV were significantly changed in the All-AFs plus Car-AF, not in Sus-AF (Table 2). Moreover, All-AFs plus Car-AF had an increased history of thromboembolism, the use of anticoagulants, and postoperative gastric care drugs considerably compared to Sus-AF. In addition, AF patients tend to have lower EF values and greater LAD values, closely related to the decline of cardiac function (Supplementary Table 1). The development of AF is accompanied by abnormally elevated concentrations of several cardiac markers, such as D2, BNP, TNT, CK-MB, and CRP. Besides, the development of AF is also associated with the presence of other structural heart diseases, such as CAD and cardiac insufficiency. In addition, the existence of factors such as hyperglycemia or bleeding history should not be ignored, which also results in the increase in CHA_2_DS_2_-VASc and HAS-BLED scores. Notably, patients with more severe AF had higher DBIL and lower ALB. Finally, treatment strategies in the late stages of AF focus more on anticoagulation, resulting in fewer antiarrhythmic drugs (Supplementary Table 2).

Relying on the difference analysis, medical routine, and collinear diagnosis (variance inflation factor <10), multivariate logistic regression was performed on the healthy and AF groups. The risk factor information was obtained by the two-way stepwise method. The results are shown in Table 3. Except for albumin, other disease-related factors found in the discovery set were challenging to reproduce in the validation set. As one of the evaluation indicators of liver function, albumin is not specific and sensitive enough for the diagnosis of AF. Abnormally low albumin levels were also found in the Sus-AF vs. Control cases, and they were not statistically different between the All-AFs plus Car-AF and Sus-AF (Table 2, Supplementary Table 1). Other multivariate logistic regression results between different groups are shown in Supplementary Table 5.

**Table 3:** Risk factor analysis of AF and Control in the discovery and validation cohort.

	**Independent variables**	**OR**	**95% CI**		***P***
Discovery cohort	SBP	1.104	1.043	1.168	0.0006
	ALB	0.187	0.094	0.370	<0.0001
	HDLC	0.173	0.022	1.326	0.0913
	LDLC	3.890	1.076	14.07	0.0383
	MCHC	1.295	1.121	1.497	0.0004
	MPV	2.337	1.232	4.432	0.0093
	GGT	1.070	1.025	1.118	0.0020
Validation cohort	DBP	1.205	1.084	1.339	0.0010
	ALB	0.290	0.166	0.507	<0.0001

**Figure 2:**
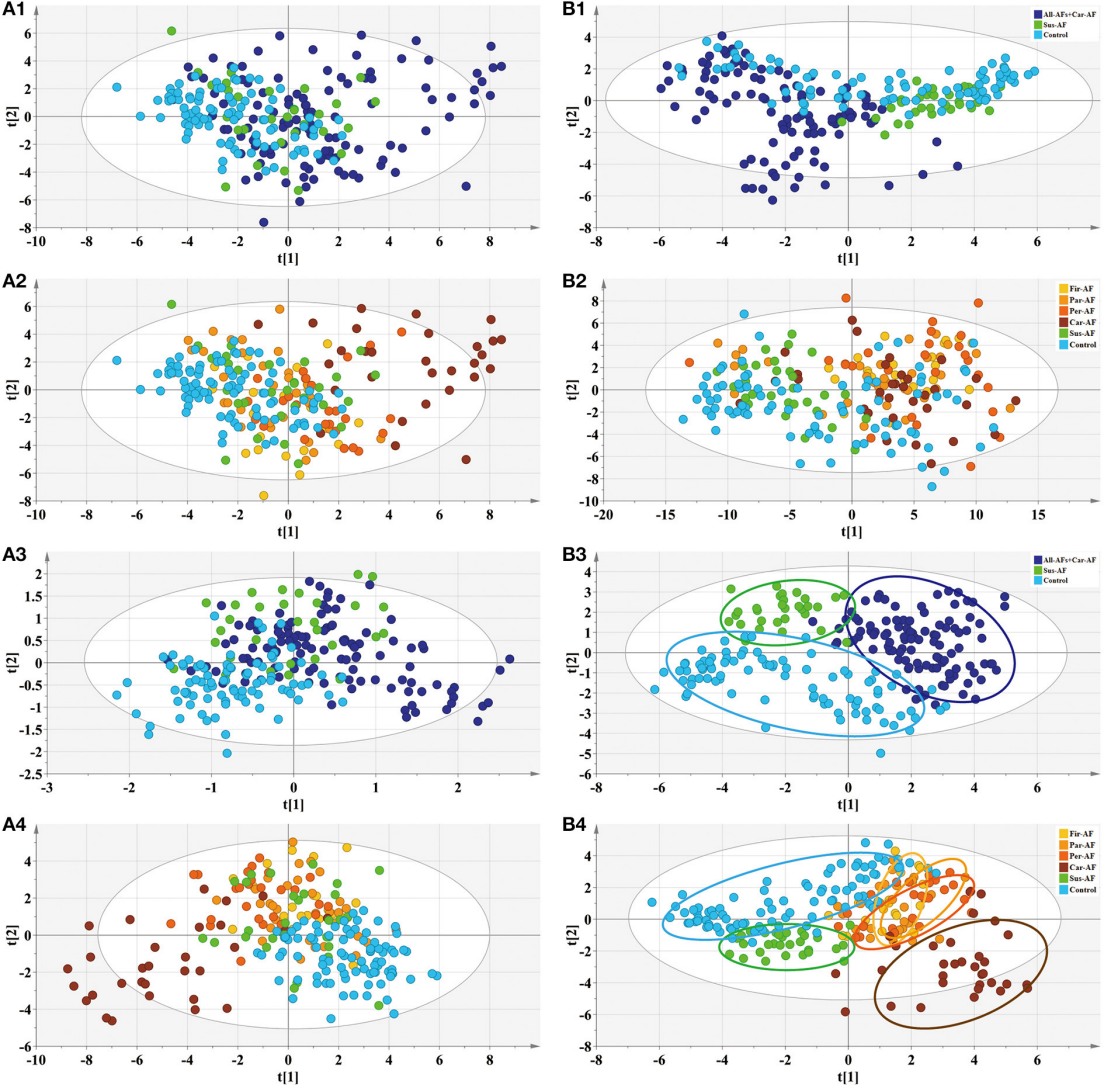
Multivariate statistical analysis differentiates the groups of Control and Experimental groups based on clinical information **(A)** and metabolomic data **(B)**, respectively. (1) PCA modeling displays the original similarity of the three groups. (2) PCA modeling displays the original similarity of the six groups. **(A1,A2)** R2X (cum) = 0.499, Q2 (cum) = 0.204; R2X (cum) = 0.499, Q2 (cum) = 0.204. **(B1,B2)** R2X (cum) = 0.504, Q2X (cum) = 0.324; R2X (cum) = 0.508, Q2X (cum) = 0.369. (3) PLS-DA modeling with the three groups. **(A3)** R2X (cum) = 0.364, R2Y (cum) = 0.463, Q2X (cum) = 0.288. Permutation tests with the intercepts of R2 < 0.125, Q2 < −0.338. **(B3)** R2X (cum) = 0.337, R2Y (cum) = 0.687, Q2X (cum) = 0.59. Permutation tests with the intercepts of R2 < 0.252, Q2 < −0.312. (4) PLS-DA modeling with the six groups. **(A4)** R2X (cum) = 0.326, R2Y (cum) = 0.342, Q2X (cum) = 0.26. Permutation tests with the intercepts of R2 < 0.079, Q2 < −0.134. **(B4)** R2X (cum) = 0.383, R2Y (cum) = 0.43, Q2X (cum) = 0.329. Permutation tests with the intercepts of R2 < 0.125, Q2 < −0.178.

Based on clinical parameters, including “demographics,” “comorbidity and medication,” “biochemical items,” “blood items,” “cardiac risk factors,” and other laboratory data, we created an unsupervised PCA score plot. When we divided the samples into three or six groups, the model showed some outliers, and each group usually overlapped with the others (Figures 2A1,A2). However, when we split the samples into three groups and ran a supervised PLS-DA model, a visible separation could be seen, with a slight overlap between the groups. When the samples were divided into six groups, the Control and Car-AF were well-separated, but the other groups showed overlap (Figures 2A3,A4). In short, the models were not powerful enough to distinguish AF from Control and Sus-AF cases based on clinical data.

### Metabolomics data quality assessment

A PCA model was established for the QC samples and the serum samples to be tested simultaneously in the platforms of GC-MS and positive and negative ion modes in LC-MS. In the discovery and validation phase, the aggregation of the QC samples in this study was good (Supplementary Figures 2A1–A4). When the QC samples were subjected to PCA alone, those samples were within the 95% confidence interval, revealing that the systematic error in sample processing and detection was small (Supplementary Figures 2B1–B4). The various ES and IS fluctuations for GC-MS and IS fluctuation for LC-MS were 5.46, 6.14, and 9.03%, respectively. The serum samples' GC/MS and LC/MS analyses aligned the metabolites with typical chromatograms (Supplementary Figure 1). After deconvolution of the GC/MS chromatograms, 175 independent peaks were identified from the serum samples, 117 of which were authenticated as metabolites; LC/MS identified 94 compounds from 303 produced peaks (Supplementary Tables 6, 7).

### Serum metabolomic description by PCA and PLS-DA plots

Metabolomics analysis revealed a few outliers in the PCA score plot when the samples were divided into three or six groups. When six groups were defined, the separation trend was still not significant, which may be attributed to specific individual differences in each group (Figures 2B1,B2). However, the PCA model showed that most All-AFs plus Car-AF deviated from the Control and Sus-AF when the three groups were defined, suggesting that the identified serum metabolites can naturally detect the difference among the three groups. When the samples were divided into three groups, the PLS-DA model revealed that samples were clustered closely and kept apart from the others (Figure 2B3). When we divided the samples into six groups, Control and Sus-AF clustered separately, while Fir-AF, Par-AF, and Per-AF primarily overlapped with one another, with a minority overlapping with the Control (Figure 2B4). As the disease progressed, the metabolic trajectories of AF patients increasingly deviated from the Control. The distant separation of Car-AF from the other groups revealed significant differences in metabolic patterns. At the same time, the overlapping of varying AF subgroups suggested similar serum metabolic ways among the Fir-AF, Par-AF, and Per-AF groups. In general, metabolomic results were better at differentiating the diagnosis of the Control, Sus-AF, and AFs (including Car-AF cases) than clinical data (Figure 2).

### Pathway analysis of differential metabolites

**Figure 3:**
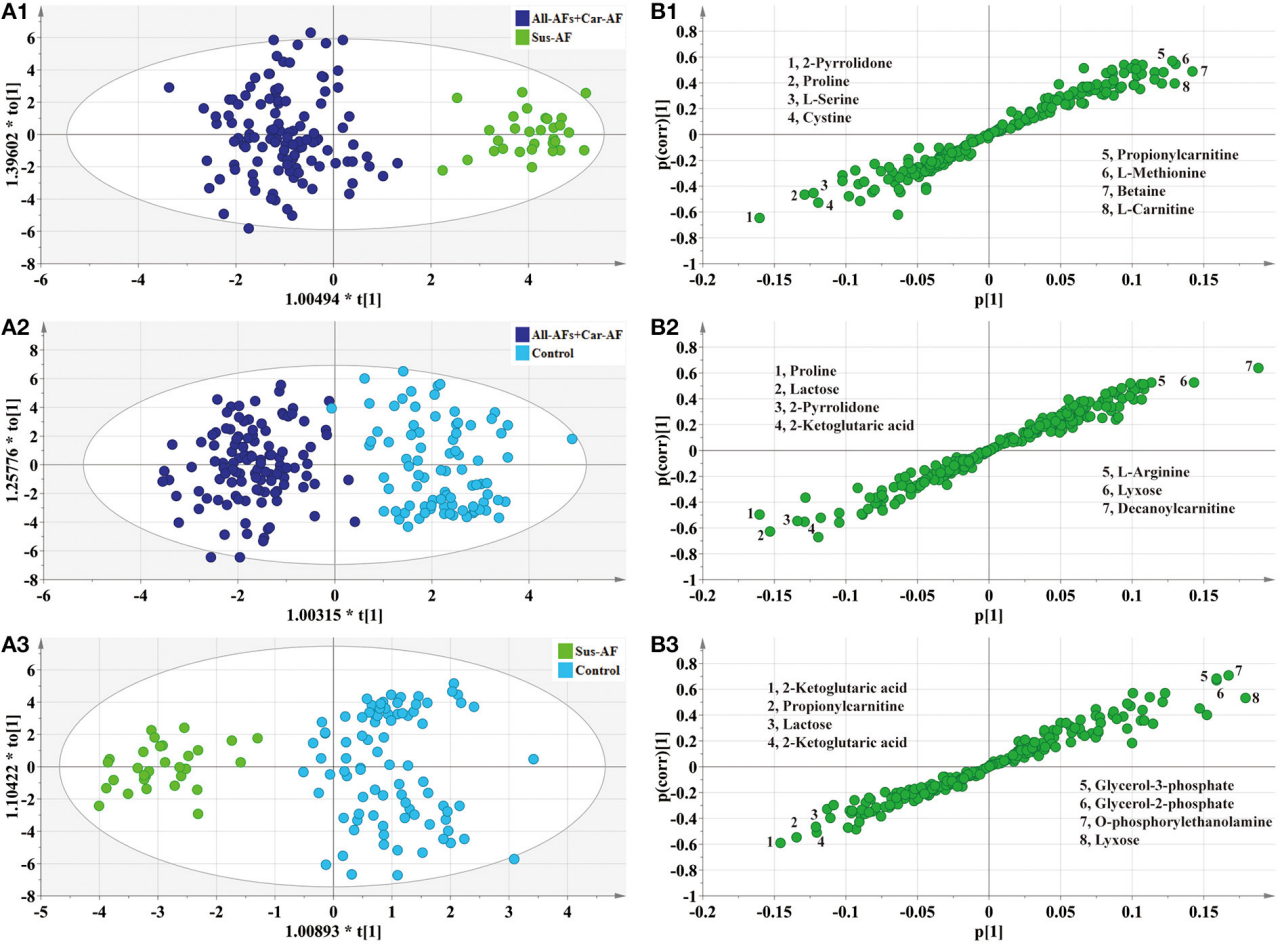
OPLS-DA modeling **(A)** and S-plots **(B)** delineate different metabolic phenotypes and potential markers of Control, Sus-AF, and All-AFs plus Car-AF cardiac cases. **(A1)** OPLS-DA model differentiating All-AFs plus Car-AF cases from the Sus-AF cases. R2X (cum) = 0.336, R2Y (cum) = 0.863, Q2X (cum) = 0.68. Permutation tests with the intercepts of R2 < 0.468, Q2 < −0.563. **(B1)** S-plot highlights the potential markers of the All-AFs plus Car-AF versus Sus-AF cases. **(A2)** OPLS-DA model differentiating All-AFs plus Car-AF cardiac cases from the Control. R2X (cum) = 0.339, R2Y (cum) = 0.848, Q2X (cum) = 0.736. Permutation tests with the intercepts of R2 < 0.368, Q2 < −0.454. **(B2)** S-plot highlights the potential markers of the All-AFs plus Car-AF versus Control cases. **(A3)** OPLS-DA model differentiating Sus-AF cases from Control. R2X (cum) = 0.301, R2Y (cum) = 0.842, Q2X (cum) = 0.704. Permutation tests with the intercepts of R2 < 0.452, Q2 < −0.475. **(B3)** S-plot highlights the potential markers of the Sus-AF versus Control cases.

**Figure 4:**
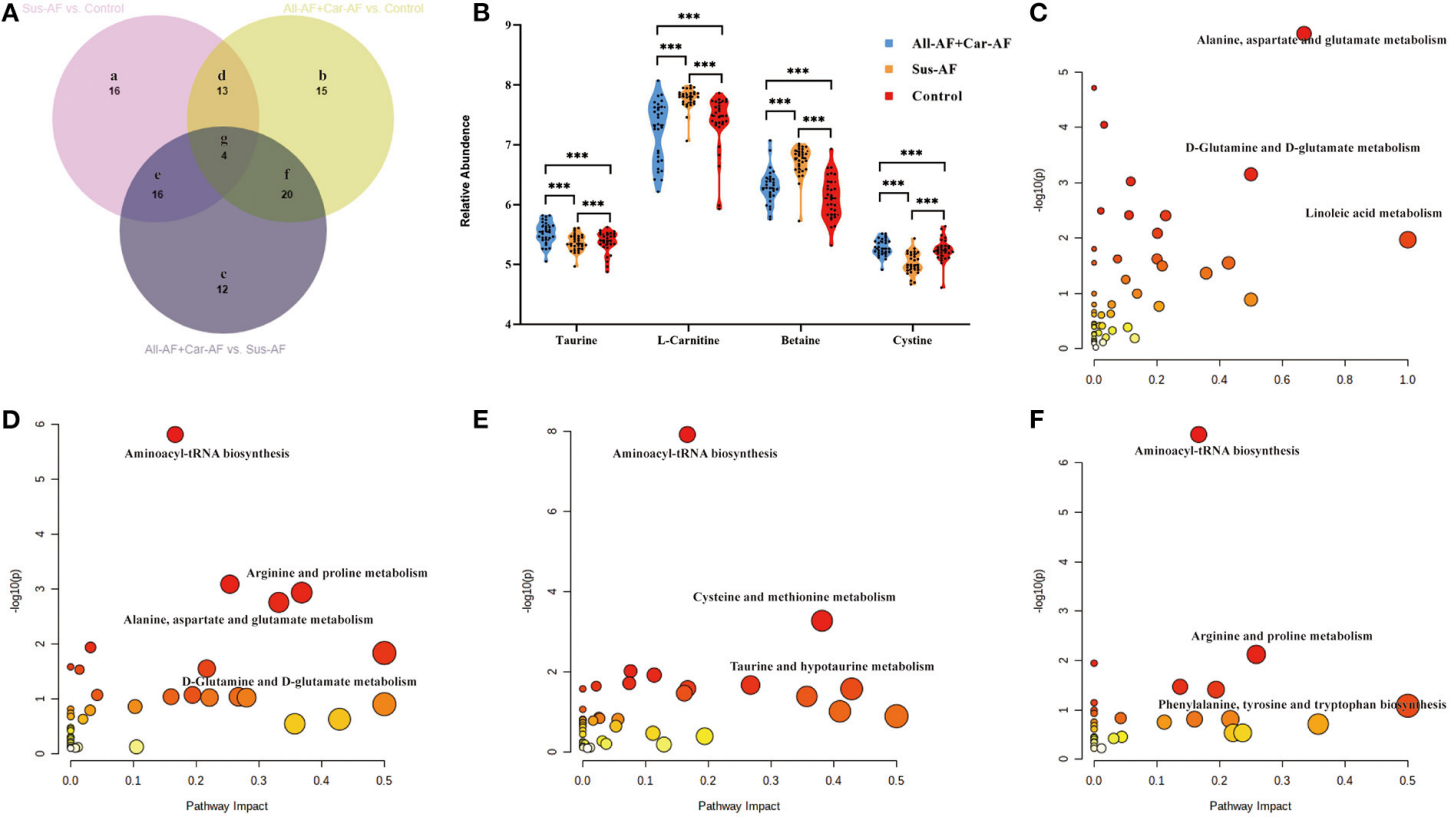
Differential metabolites and pathways involved in the experimental groups. **(A)** Venn diagram shows discriminant metabolites can be classified into regions a, b, c, d, e, f, and g. **(B)** Serum taurine, L-carnitine, betaine, and cystine levels change in the three comparisons. **(C)** Pathway analysis of differential metabolites in Venn a+d+e+g region. **(D)** Pathway analysis of differential metabolites in Venn b+d+f+g region. **(E)** Pathway analysis of differential metabolites in Venn c+e+f+g region. **(F)** Pathway analysis of differential metabolites in Venn b+g. ^***^p < 0.01.

First, OPLS-DA analysis revealed that Sus-AF, All-AFs plus Car-AF, and Control had different metabolic patterns (Figures 3A1–A3, S-plot: Figures 3B1–B3). According to the statistical analysis, 52, 52, and 49 differential metabolites were identified from the All-AFs plus Car-AF vs. Sus-AF, Control vs. All-AFs plus Car-AF, and Control vs. Sus-AF participants, respectively (Supplementary Table 8). We then created a Venn diagram to show the discriminant metabolites among the three groups compared with each other (Figure 4A). As shown, 15 metabolites in the region (b) exist independently of the different substances obtained by comparing other groups, suggesting that they are the most likely risk markers for the onset and development of AF. Notably, the overlapping region (g) lists that four metabolites, i.e., taurine, L-carnitine, betaine, and cystine, were simultaneously significant in different comparisons (Figure 4B). Figures 4C–E shows the pathway analysis of the differential metabolites. Compared with Control, All-AFs plus Car-AF and Sus-AF had similar pathways, such as alanine, aspartate, and glutamate metabolism, D-glutamine, and D-glutamate metabolism, etc. (Figures 4C,D). Enrichment and pathway analysis of the All-AFs plus Car-AF vs. Sus-AF showed that cysteine and methionine metabolism, and taurine and hypotaurine metabolism were the most altered metabolic pathways (Figure 4E). The pathway of phenylalanine, tyrosine, and tryptophan biosynthesis was deranged in the All-AFs plus Car-AF cases (Figure 4F), as shown by abnormal changes in AF-specific metabolites.

### Cross comparisons to and within subgroups

**Figure 5:**
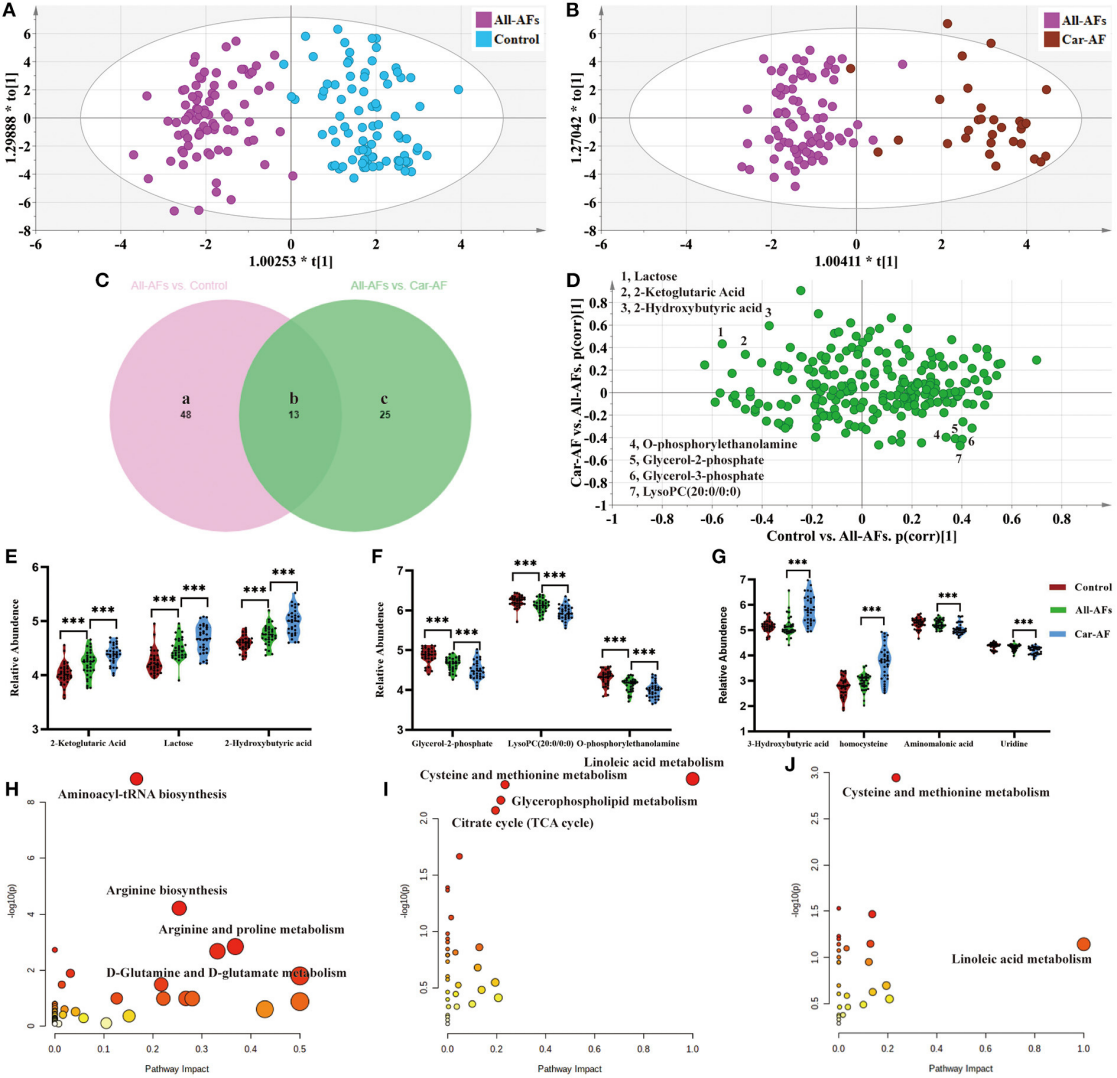
Differential metabolites and pathways involved in different groups. **(A)** OPLS-DA modeling delineates different metabolic phenotypes of Control and All-AFs cardiac cases. R2X(cum) = 0.343, R2Y(cum) = 0.861, Q2X(cum) = 0.712. Permutation tests with the intercepts of R2 <0.454, Q2 < −0.542. **(B)** OPLS-DA model differentiating Car-AF patients from the All-AFs cases. R2X(cum) = 0.302, R2Y(cum) = 0.851, Q2X(cum) = 0.74. Permutation tests with the intercepts of R2 <0.445, Q2 < −0.515. **(C)** Venn diagram shows discriminant metabolites can be classified into regions a, b, and c. **(D–G)** SUS-plots and violin diagram delineate potential markers of Control, Car-AF, and All-AFs cardiac cases. SUS-plot highlights the potential markers of the All-AFs, Car-AF, and Control cases. "* * *" means p?0.01. **(H)** Pathway analysis of differential metabolites in Venn a and b regions. **(I)** Pathway analysis of differential metabolites in Venn b and c. **(J)** Pathway analysis of differential metabolites in Venn c. ^***^p < 0.01.

We first focused on All-AFs vs. Car-AF for cardiac stroke and Control vs. All-AFs for the development of AF. The OPLS-DA model revealed 61 differential metabolites between Controls and All-AFs (Figure 5A) (Supplementary Table 8). Similarly, Car-AF cases primarily showed different metabolomic patterns from All-AFs (Figure 5B), and 38 discriminant metabolites were identified (Supplementary Table 8). We also created a Venn diagram to show the discriminant metabolites between the two comparisons (Figure 5C). The SUS-plot also delineates the potential markers among the Control, Car-AF, and All-AFs cases (Figure 5D). Of the metabolites differentiating All-AFs from the Control, the levels of 2-ketoglutaric acid, lactose, and 2-hydroxybutyric acid were higher in All-AFs, glycerol-2-phosphate, O-phosphorylethanolamine, and LysoPC (20:0/0:0) were lower. All of the above metabolites deviated further in Car-AF (Figures 5E,F). These findings indicate that the above metabolites are involved in the development of AF. Moreover, different levels of compounds, including 3-hydroxybutyric acid, homocysteine, aminomalonic acid, and uridine, were only observed in Car-AF (vs. All-AFs), which indicated their association with the development of cardiogenic cerebral embolism caused by AF (Figure 5G). Figures 5H,I illustrate the pathway analysis of the metabolites in Control vs. All-AFs and All-AFs vs. Car-AF. Generally, aminoacyl-tRNA biosynthesis, arginine biosynthesis, and arginine and proline metabolism were the most significantly altered metabolic pathways in All-AFs compared with the Control (Figure 5H). The enrichment and pathway analysis for the metabolites of the Venn b and c area showed that glycerophospholipid metabolism and citrate cycle were the most altered metabolic pathway (Figure 5I). Cysteine and methionine metabolism and linoleic acid metabolism also deserve attention in Car-AF (Figure 5J).

**Figure 6:**
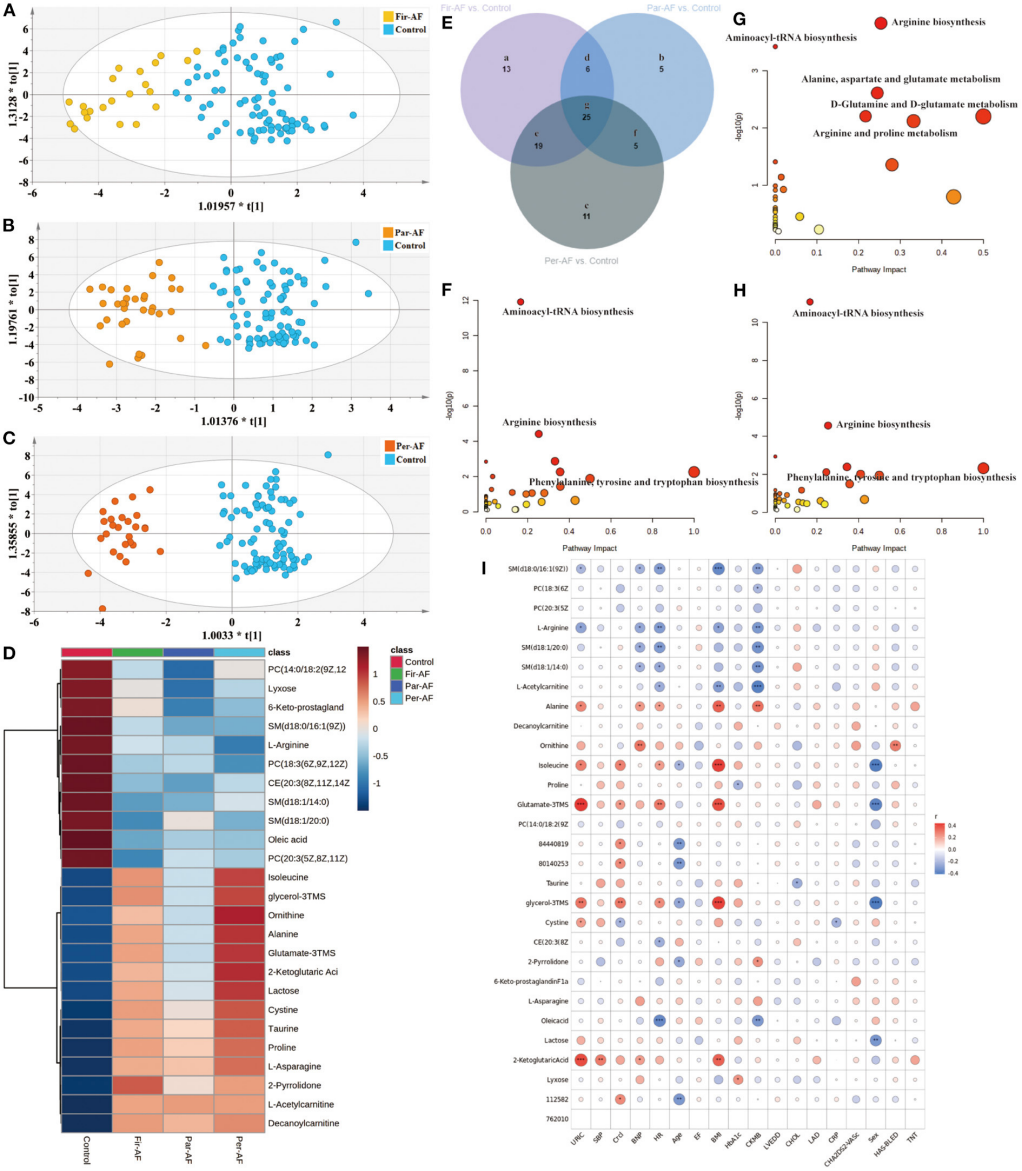
Differential metabolites and pathways involved in the AF groups. **(A–C)** OPLS-DA modeling delineates different metabolic phenotypes of Fir-AF, Par-AF, and Per-AF cardiac cases from Control. R2X (cum) = 0.272, R2Y (cum) = 0.679, Q2X (cum) = 0.536; Permutation tests with the intercepts of R2 < 0.325, Q2 < -0.34. R2X (cum) = 0.315, R2Y (cum) = 0.812, Q2X (cum) = 0.633; Permutation tests with the intercepts of R2 < 0.458, Q2 < -0.473. R2X (cum) = 0.351, R2Y (cum) = 0.893, Q2X (cum) = 0.706; Permutation tests with the intercepts of R2 < 0.323, Q2 < -0.312. **(D)** Heatmap of the 25 differential metabolites in Control and all kinds of AFs. The colors changing from blue to red indicate high metabolites. **(E)** Venn diagram shows discriminant metabolites can be classified into 7 regions. **(F)** Pathway analysis of differential metabolites in Venn a, d, e, and g regions. **(G)** Pathway analysis of differential metabolites in Venn b, d, f and g. **(H)** Pathway analysis of differential metabolites in Venn c, e, f, and g regions. **(I)** The correlation coefficients between 25 potential biomarker contents and clinical characteristics. Blue represents negative correlation, and red represents positive correlation. Circle size represents the *r*-value of the metabolites and clinical characteristics. The symbol from ^*^ to ^**^ and ^***^ indicate the *P*-values between metabolites and clinical characteristics. ^*^p < 0.05, ^**^p < 0.01, and ^***^p < 0.001.

Next, we separately analyzed the differences between Control and the three AF subtypes (Figures 6A–C). The metabolites for each comparison appear in Supplementary Table 8. Figure 6E is a Venn diagram showing the discriminant metabolites between different kinds of AF and Control. We also made a heat map to show the average normalized quantities of the 25 differential metabolites (Venn g region) in the above groups (Figure 6D). Among them, 12 metabolites were elevated, and 13 metabolites decreased in order according to the following groups: Control, Par-AF, and Per-AF. A correlation analysis between clinical characteristics and 25 potential biomarkers is shown in Figure 6I. Figures 6F–H represent the perturbed pathways. For Fir-AF and Per-AF vs. Control, metabolism changed for aminoacyl-tRNA biosynthesis, arginine biosynthesis, phenylalanine, tyrosine, and tryptophan biosynthesis; for Par-AF vs. Control, alanine, aspartate, and glutamate metabolism, D-glutamine and D-glutamate metabolism, as well as arginine and proline metabolism, also changed significantly. We also found that the metabolomics profiles and metabolic pathways could distinguish cardiovascular patients (CP, including Sus-AF, all kinds of AF, and Car-AF) from Control and different subtypes of AFs from each other (Supplementary Table 8; Supplementary Figures 3, 4).

### Differential diagnosis of metabolic biomarkers

Early recognition and accurate diagnosis of different subtypes are fundamental for personalized treatment of AF. We summarized six specific metabolic biomarkers for distinguishing All-AFs add Car-AF vs. Control, five for All-AFs versus Car-AF, seven for Par-AF vs. Control, and six for Par-AF vs. Per-AF (Table 4). Odds ratios for the biomarker panels were obtained after adjustment for possible confounders in all cross-comparisons. Additional metabolomics-based biomarker comparisons are provided in Supplementary Table 9.

**Table 4:** Statistical analysis of diagnostic biomarkers: Discovery phase.

**Differential metabolites**	**AUROC**	**95% CI**		**Sensitivity** **(%)**	**Specificity** **(%)**	**LogOR**	**95%CI**	
		**Lower**	**Upper**				**Lower**	**Upper**
**Comparison I: all-AFs plus car-AF (*****n*** **=** **113) vs. control (*****n*** **=** **87)**								
Lactate	0.8684	0.8176	0.9191	82.30%	80.23%	6.41	4.60	8.22
D-glutamic acid	0.8241	0.7660	0.8822	86.73%	67.44%	4.62	3.22	6.03
Decanoylcarnitine	0.8725	0.8207	0.9243	86.05%	80.53%	2.37	1.71	3.03
SM (d18:1/14:0)	0.7985	0.7346	0.8625	86.05%	64.60%	−2.85	−3.76	−1.93
LysoPC (P-18:0)	0.7883	0.7236	0.8530	73.26%	72.57%	−3.39	−4.60	−2.18
2-pyrrolidone	0.8209	0.7596	0.8823	86.73%	72.94%	2.50	1.78	3.23
Lactate, decanoylcarnitine, lysoPC(P-18:0), and 2-Pyrrolidone	0.9426	0.9111	0.9741	94.69%	80.00%	2.64	2.03	3.26
Adjust panel	0.9535	0.9248	0.9822	86.67%	91.76%	2.72	2.09	3.36
**Comparison II: all-AFs (*****n*** **=** **81) vs. car-AF (*****n*** **=** **32)**								
3-hydroxybutyric acid	0.8642	0.7918	0.9366	78.13%	82.72%	−1.34	−1.86	−0.82
Homocysteine	0.8473	0.7551	0.9395	65.63%	97.47%	−1.49	−2.09	−0.90
Ribitol	0.8777	0.8026	0.9528	87.50%	82.72%	−3.53	−4.96	−2.10
Methyl galactoside	0.8106	0.7231	0.8980	71.88%	81.48%	−2.99	−4.45	−1.54
Citrate	0.7986	0.7078	0.8894	75.00%	76.54%	−3.23	−4.73	−1.74
Citrate, ribitol, and homocysteine	0.9351	0.8896	0.9807	97.47%	71.88%	−2.70	−3.66	−1.74
Adjust panel	0.9739	0.9395	1.0000	97.47%	87.50%	−3.61	−5.30	−1.91
**Comparison III: par-AF (*****n*** **=** **33) vs. control (*****n*** **=** **87)**								
D-glutamic acid	0.7572	0.6585	0.8559	81.82%	67.44%	3.35	1.66	5.05
Lyxose	0.8161	0.7396	0.8925	60.47%	90.91%	−2.21	−3.25	−1.17
Lactose	0.7703	0.6778	0.8627	87.88%	62.79%	2.04	0.98	3.10
CE [20:3(8Z,11Z,14Z)]	0.8055	0.7157	0.8953	74.42%	81.82%	−2.00	−2.93	−1.06
Decanoylcarnitine	0.9070	0.8493	0.9647	89.53%	84.85%	2.49	1.58	3.41
SM (d18:1/14:0)	0.7907	0.6970	0.8844	86.05%	66.67%	−2.61	−3.83	−1.39
2-pyrrolidone	0.8000	0.7162	0.8838	87.88%	67.06%	2.34	1.31	3.36
Lyxose, lactose, and decanoylcarnitine	0.9648	0.9342	0.9954	93.94%	88.37%	2.88	1.98	3.78
Adjust panel	0.9891	0.9765	1.0000	100.00%	90.70%	3.38	2.22	4.54
**Comparison IV: par-AF (*****n*** **=** **33) vs. per-AF (*****n*** **=** **26)**								
Glycolic acid	0.7541	0.6281	0.8801	88.46%	60.61%	3.67	1.28	6.05
Alanine	0.7832	0.6618	0.9046	76.92%	78.79%	3.42	1.13	5.71
D-malic acid	0.8275	0.7232	0.9318	92.31%	66.67%	4.24	2.00	6.47
Citrate	0.7634	0.6349	0.8919	76.92%	69.70%	3.39	1.28	5.51
Tyrosine	0.7914	0.6703	0.9124	76.92%	78.79%	5.30	1.99	8.62
2-hydroxy-3-methylbutyric acid	0.7517	0.6262	0.8773	61.54%	81.82%	2.34	0.86	3.82
D-malic acid, tyrosine, and 2-hydroxy-3-methylbutyric acid	0.9114	0.8404	0.9824	80.77%	90.91%	2.47	1.36	3.58
Adjust panel	0.9347	0.8720	0.9975	88.46%	87.88%	2.50	1.45	3.55

**Figure 7:**
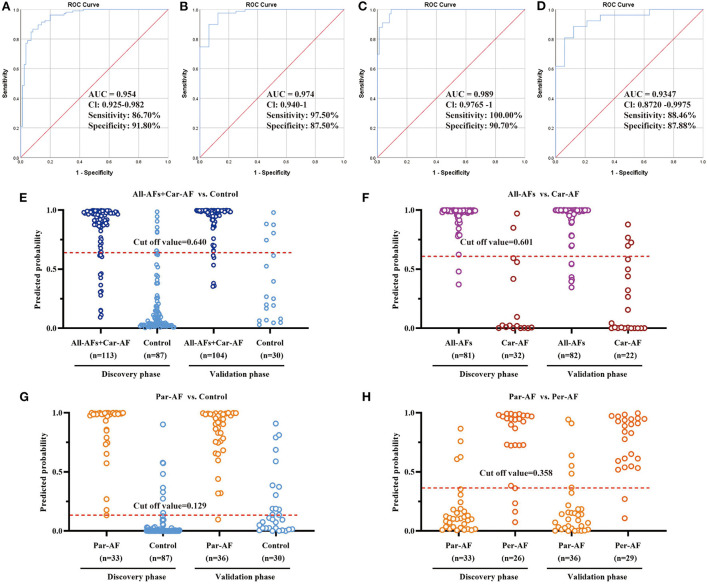
Diagnostic Outcomes and Prediction Accuracies. The diagnostic outcomes in the discovery phase are shown via the ROC curves for comparison between **(A)** All-AFs plus Car-AF versus Control, **(B)** All-AFs versus Car-AF, **(C)** Par-AF versus Control, and **(D)** Par-AF versus Per-AF. The prediction accuracies by the biomarkers in the validation set were compared between **(E)** All-AFs plus Car-AF versus Control, **(F)** All-AFs versus Car-AF, **(G)** Par-AF versus Control, and **(H)** Par-AF versus Per-AF. AUC, area under the curve; CI, confidence interval.

Based on the logistic regression for each biomarker panel, we created the ROC presentations in the discovery phase (Figure 7). The AUC, sensitivity, and specificity were 0.954, 86.7, and 91.8% for All-AFs plus Car-AF vs. Control (*n* = 200) (Figure 7A); 0.974, 97.5, and 87.5% for All-AFs vs. Car-AF (*n* = 113) (Figure 7B); 0.989, 100, and 90.7% for Par-AF vs. Control (*n* = 120) (Figure 7C); and 0.935, 88.5, and 87.9% for Par-AF vs. Per-AF (*n* = 59) (Figure 7D), respectively. For additional cross-comparisons, AUCs ranged from 0.8237 to 0.9844, sensitivities ranged from 87.88 to 100%, and specificities ranged from 63.64 to 100% (Supplementary Figure 5). ROC curves with AUC, sensitivity, and specificity values for the four cross-comparisons in the validation phases are shown in Supplementary Figure 6.

According to the highest prediction sensitivity and specificity of the ROC in the discovery phase, the optimal cut-off values were 0.640 for All-AFs plus Car-AF vs. Control, 0.601 for All-AFs vs. Car-AF, 0.129 for Par-AF vs. Control, and 0.358 for Par-AF vs. Per-AF. We then used the cut-off values to predict the different groups of AFs in the validation phase. Predictive values were 90.2% for All-AFs plus Car-AF vs. Control (Figure 7E); 90.1% for All-AFs vs. Car-AF (Figure 7F); 78.8% for Par-AF vs. Control (Figure 7G); and 86.2% for Par-AF vs. Per-AF (Figure 7H).

## Discussion

This work described a comprehensive clinical metabolomic evaluation for 363 participants who underwent ECG in the discovery and validation phases. First, we collected a large amount of clinical data and analyzed the risk factors. Known risk factors contributing to the onset and progression of AF include obesity, obstructive sleep apnea, hyperlipidemia, smoking, alcohol, physical inactivity, genetics, aortic stiffness, and some traditional factors, such as age, hypertension, heart failure, diabetes, and valvular heart disease (36). There are still some potential removable causes of AF, such as: early onset of unknown AF in channelopathies like Brugada (37) and long QT syndrome (38), hyperthyroidism (39), and also lifestyle factors such a regular practice of endurance sports (40). In addition, some biomarkers have also been identified as potential AF risk factors, such as myocardial injury (troponin), cardiovascular stress and dysfunction (natriuretic peptides, growth differentiation factor 15), myocardial fibrosis (galectin-3), renal dysfunction (creatinine, cystatin C), inflammation (C-reactive protein, cytokines), and coagulation activity (D-dimer). However, none of those are used in clinical practice (41). Regrettably, the results we obtained also have poor reproducibility, sensitivity, and specificity. Usually, prediction models based solely on clinical factors require a large sample size per group, which was limited in our study by the complexity, integrity, and high costs of patient data collection (42, 43). Metabolic phenotypes revealed significant pattern differences between patients with AF, no AF, and within AF subtypes. We found a set of diagnostic markers unique to patients with AF compared to healthy individuals who are not affected by suspected AF. Then, we found some metabolic features that were unique to AF-induced stroke. Lastly, 25 common significantly regulated metabolites in serum from different subtypes of AF patients, suggesting that AF may involve a universal metabolic disturbance. Combinations of metabolic biomarkers offered excellent predictive values for AF onset, progression, and differential diagnosis.

AF has traditionally been considered a cardiac arrhythmia caused by disorganized electrical impulses, usually originating at the root of the pulmonary veins (44). However, accumulating evidence suggests a correlation between metabolism disorders and the occurrence of AF (45). Compared with healthy participants, patients with AF had down-regulated oleic acid and upregulated D-glutamic acid and uric acid. In a long-term cohort study, oleic acid intake significantly decreased the risk of cardiovascular disease (46). Oleic acid prevents coronary heart disease by suppressing oxidative stress and mitigating myocardial cell damage (47). Serum uric acid is also a surrogate indicator of oxidative stress (48). D-glutamic acid might activate transporter-related Cl–conductance and regulate neuronal transmission (49), affecting the autonomic nervous system and involving AF's pathogenesis (50). In addition, we identified a panel of discriminant metabolites also considered as potential markers of AF in previous reports, such as L-lysine (51), valine (52), creatinine (53), tyrosine (54), stearic acid (55), methionine (56), and alanine (57). Moreover, the changes in L-acetylcarnitine and decanoylcarnitine suggest that carnitine metabolism dysfunction also contributes to the occurrence of AF (58, 59). Stressed myocardial cells can transform from fatty acid oxidation to glycolysis in the mitochondria (60). The long-chain acylcarnitine then accumulates in the cytoplasm, contributing to membrane instability by inhibiting the exchange of sodium and calcium ions in the sarcolemma, ultimately leading to arrhythmias (61). Decenoylcarnitine is an oxidative metabolite that is derived from a fatty acid belonging to the acylcarnitines (ACs) family (62). Carnitine molecules shuttle across the membrane, while acyl-CoA participates in the production of ATP through the β-oxidation pathway. ACs play an essential role in the daily energy production of the heart. It is of particular interest that decanoylcarnitine may be associated with AF-induced cardioembolic stroke (63).

Notably, several lines of evidence suggest that rhythm alteration alone may not fully account for the risk of stroke attributed to AF (64). The causal relationship between AF and stroke may be more complex than anticipated initially. Compared with the All-AFs, the Car-AF had upregulation of lactose and homocysteine. Usually, energy metabolism dysfunction is closely related to cerebral ischemia-reperfusion injury (65). Lactate may arise from the transition of potentially viable cells to anaerobic glycolysis, which continues to metabolize glucose under localized hypoxia (66). As one of the proposed risk factors for stroke, total serum homocysteine is an intermediate product of one-carbon-cycle metabolites. The increased level responds to the acute phase of cerebral ischemia (67). In addition, Nelson et al. (68) used LC/MS to determine 144 metabolites from 367 acute stroke patients, compared cardiac and non-cardiogenic samples, and they found that the tricarboxylic acid metabolite α-ketoglutarate and malate were associated with cardiogenic stroke, which is encouragingly consistent with our findings. Meanwhile, other metabolites we found have also been widely reported by researchers, for example, the changing content of uridine (69), 3-hydroxybutyric acid (70), L-cysteine (71), and asymmetric dimethylarginine (72).

Early detection of paroxysmal AF allows one to take precautions against or reduce undesirable disease outcomes through appropriate treatment strategies (73). In recent years, it has been widely theorized that released cytokines may lead to inflammation, which alters the function and expression of cardiac ion channels, ultimately affecting the atrial structure and electrophysiological remodeling (74). In this study, we found four low levels of LysoPCs in AF patients. Accumulated evidence indicates that LysoPCs species are protective in anti-inflammatory responses and metabolic disease progression (75, 76). Sphingolipids and glycerophospholipids, which are major cellular membrane components, have been reported to influence cell membranes' physical properties and protein function (77). According to previous studies, low levels of SMs are associated with the insufficient capacity of the organism to cope with oxidative stress, showing high levels of peroxidation and proinflammatory precursors (78). In addition, we also found higher levels of three SMs in the Control compared to AF patients. However, the molecular mechanism of lipid alterations associated with AF requires further study in more detailed experiments. Interestingly, Alvaro Alonso et al. performed a metabolomic analysis in the combined sample of 3,922 participants from the Atherosclerosis Risk in Communities Study. The author replicated a prospective association among a previously identified secondary bile acid, glycocholenate sulfate, and AF incidence and identified new metabolites involved in nucleoside and polyamine metabolism as markers of AF risk (79). Unfortunately, our study did not replicate Alvaro Alonso's results well-due to differences in ethnicity and lifestyle or insufficient types of metabolite detection that warrant further exploration.

Accurate diagnosis of different subtypes of AF facilitates precise treatment by physicians. We conducted extensive comparisons of different subtypes of AF with each other, Control and Car-AF. First, we found that the metabolic trajectory of Fir-AF was challenging to separate from paroxysmal and persistent AF (Figure 2B4, Supplementary Figures 3B,C), as many people might be diagnosed with one of the two types for the first time. Second, the metabolic differences between the three types of AF and Car-AF were huge (Figure 2B4, Supplementary Figures 3F–3H), which may support that anticoagulation in AF patients depends only on the CHA_2_DS_2_-VASc score, not different subtypes (80). Interestingly, some potential metabolic markers and altered metabolic pathways were found to exist between Par-AF and Per-AF (Supplementary Figures 3D, 4D; Supplementary Table 8). With the aggravation of the disease, the content of the common differential metabolites between the two groups also further deviated from the Control (Figure 6D), which may provide suggestions for different ablation surgical strategies or individualized use of antiarrhythmic drugs. Finally, we found that potential metabolic markers associated with AF were highly correlated with clinically recognized risk factors (Figure 6I), further supporting our conclusion.

## Conclusions

We reported a comprehensive metabolomic evaluation for identifying clinically relevant perturbations in circulating metabolites in AF. This evaluation improves the understanding of AF pathogenesis and facilitates target screening for therapeutic intervention. Novel biomarkers predict and differentiate between AF types. The metabolic biomarkers' sensitivity, specificity, and predictive value align with expectations and can complement existing diagnostic methods. The potential applications in clinical diagnosis include: (1) differential diagnosis of AF vs. Sus-AF; (2) if AF is not significant, a differential diagnosis of Par-AF vs. Control is required; and (3) a differential diagnosis of Per-AF vs. Par-AF is also vital for individualized treatment. These biomarkers separate individuals who may benefit from additional testing with ECG from individuals who will not.

## Study limitations

First, the inclusion criteria for asymptomatic AF paroxysmal patients might be potentially biased since the ECG Holter monitor could not determine an AF diagnosis. Second, the sample size was small, lack of participation in multiple clinical centers, and no other types of samples were collected, limiting the results' reliability. Third, we did not use other metabolomics platforms to detect more metabolites, and we did not use LC/MS-MS methods for the absolute quantification of potential markers, which was not conducive to clinical application. Fourth, our study population consisted of middle-aged to elderly Chinese patients. Future studies could broaden the scope to include other ethnicities within Asia and other races such as Caucasians and Africans. Lastly, the metabolic mechanisms that lead to disease occurrence and progression remain unclear owing to the lack of in-depth exploration in our study through technical means such as molecular biology, cell biology, and pharmacology.

## Data availability statement

The original contributions presented in the study are included in the article/Supplementary material, further inquiries can be directed to the corresponding authors.

## Ethics statement

The studies involving human participants were reviewed and approved by the Ethics Committee of the Nanjing Drum Tower Hospital, The Affiliated Hospital of Nanjing University Medical School. The patients/participants provided their written informed consent to participate in this study.

## Author contributions

WX, YP, and JS were responsible for the study's design. The LC/MS and GC/MS platforms were provided by Jiye A and WG. JB and WX confirmed the diagnosis of AFs and Control cases. CheL, ChuL, and DM recorded the medical history of the volunteers and collected the serum samples. CheL and XY performed the metabolomics. CheL analyzed the data, produced the figures and tables, and wrote the manuscript. MY, XB, MW, and KF gave valuable statistics and data processing suggestions. All authors contributed to the article and approved the submitted version.

## Funding

This work was supported by the Key Project of Jiangsu Provincial Health Committee (HA093-2021).

## Conflict of interest

The authors declare that the research was conducted in the absence of any commercial or financial relationships that could be construed as a potential conflict of interest.

## Publisher's note

All claims expressed in this article are solely those of the authors and do not necessarily represent those of their affiliated organizations, or those of the publisher, the editors and the reviewers. Any product that may be evaluated in this article, or claim that may be made by its manufacturer, is not guaranteed or endorsed by the publisher.

## Abbreviations

AF: atrial fibrillation
ECG: electrocardiogram
Fir-AF: first diagnosed AF
Par-AF: paroxysmal AF
Per-AF: persistent AF
AUC: areas under the curve
OR: odd ratio
QC: quality control
CV: correlation of variation
LysoPC: lysophosphatidylcholine
MG: monoglyceride
PC: phosphatidylcholine
SM: sphingomyelin
PE: phosphatidylethanolamine
CE: cholesteryl esters
PI: phosphatidylinositol.
