# Supplementary material for: Comprehensive metabolomic characterization of atrial fibrillation
Source: Front Cardiovasc Med. 2022 Aug 8;9:911845. doi: 10.3389/fcvm.2022.911845 (PMC9393302; doi:10.3389/fcvm.2022.911845)
Supplement: Supplementary file 1 [file Table_1.DOCX]

**Supplemental Table 1.** Baseline Characteristics of Discovery Phase Patients

|  | Variable | Sus-AF (n=30) | All-AFs plus Car-AF (n=113) | Effect size;95%CI | t/Z/χ2-value | P |
| --- | --- | --- | --- | --- | --- | --- |
| Demogra-phics | Male | 14 (46.7) | 73 (64.6) | 17.9 (-37.8-2.0) | 3.20 | 0.074 |
|  | Age | 54.83 ± 9.57 | 65.59 ± 11.66 | 10.76 (6.19-15.33) | 4.65 | ＜0.001 |
|  | Weight | 65.68 ± 9.13 | 69.99 ± 11.91 | 4.19 (-0.46-8.85) | 1.78 | 0.077 |
|  | Height | 1.67 ± 0.08 | 1.68 ± 0.08 | 0.01 (-0.02-0.04) | 0.69 | 0.492 |
|  | BMI | 23.62 ± 2.36 | 24.7 ± 3.31 | 1.09 (-0.21-2.38) | 1.66 | 0.100 |
|  | BSA | 1.82 ± 0.15 | 1.88 ± 0.18 | 0.06 (-0.01-0.13) | 1.63 | 0.105 |
|  | Smoking | 4 (13.3) | 28 (24.8) | 11.5 (-26.0-3.0) | 1.79 | 0.181 |
|  | Drinking | 3 (10.0) | 21 (18.6) | 8.6 (-21.5-4.3) | 1.25 | 0.263 |
|  | Family | 5 (16.7) | 8 (7.1) | -9.6 (-4.6-23.8) | 1.60 | 0.205 |
|  | Day | 4.2 ± 1.7 | 9.4 ± 6.0 | 5.2 (3.9-6.4) | 8.10 | ＜0.001 |
|  | SBP | 128.60 ± 16.36 | 136.91 ± 20.33 | 8.31 (0.36-16.26) | 2.07 | 0.041 |
|  | DBP | 81.73 ± 11.74 | 82.59 ± 12.49 | 0.86 (-4.15-5.87) | 0.34 | 0.735 |
|  | HR | 71.4 ± 11.3 | 76.5 ± 17.0 | 5.1 (-0.1-10.3) | 1.95 | 0.123 |
|  | HbAlc | 5.6 (5.4, 5.8) | 5.9 (5.5, 6.3) | 0.3 (0.1-0.5) | -3.00 | 0.003 |
|  | CRP | 5.6 (3.6, 6.6) | 3.1 (2.2, 4.7) | -1.7 (-2.7--0.6) | -2.79 | 0.005 |
|  | Crcl | 106.74 ± 31.19 | 86.96 ± 36.92 | -16.30 (-30.22--2.38) | -2.32 | 0.022 |
| Comorbidity | Bleeding | 0 (0.0) | 12 (10.6) | 10.6 (-16.3--4.9) | 2.23 | 0.135 |
|  | Embolism | 1 (3.3) | 42 (37.2) | 33.9 (-44.9--22.9) | 12.91 | ＜0.001 |
|  | T2DM | 3 (10.0) | 18 (15.9) | 5.9 (-18.6-6.8) | 0.28 | 0.599 |
|  | Hyperlipidemia | 5 (16.7) | 18 (15.9) | -0.8 (-14.2-15.8) | 0.00 | 1.000 |
|  | Hepatopathy | 2 (6.7) | 11 (9.7) | 3.0 (-13.5-7.5) | 0.03 | 0.871 |
|  | Nephropathy | 4 (13.3) | 11 (9.7) | -3.6 (-9.7-16.9) | 0.06 | 0.813 |
|  | Pulmonaryhypertension | 0 (0.0) | 15.0 (13.3) | 13.3 (-19.6--7.0) | 3.15 | 0.076 |
|  | Pulmonary | 4 (13.3) | 46.0 (40.7) | 27.4 (-42.6--12.2) | 7.81 | 0.005 |
|  | Blood | 1 (3.3) | 28 (24.8) | 21.5 (-31.7--11.3) | 6.74 | 0.009 |
|  | HFNYHA | 0 (0.0) | 13 (11.5) | 11.5 (-17.4--5.6) | 2.53 | 0.112 |
|  | Hypertension | 15 (50.0) | 54 (47.8) | -2.2 (-17.9-22.3) | 0.05 | 0.829 |
|  | CAD | 2 (6.7) | 12 (10.6) | 3.9 (-14.5-6.7) | 0.09 | 0.763 |
|  | Heart | 20 (66.7) | 38 (33.6) | -33.1 (14.1-52.1) | 10.73 | 0.001 |
| Medication | Anticoagulants | 0 (0.0) | 34 (30.1) | 30.1 (-38.6--21.6) | 11.84 | 0.001 |
|  | Amiodarone | 0 (0.0) | 29 (25.7) | 25.7 (-33.8--17.6) | 9.66 | 0.002 |
|  | Propafenone | 2 (6.7) | 22 (19.5) | 12.8 (-24.4--1.3) | 2.78 | 0.095 |
|  | Digoxin | 0 (0.0) | 5 (4.4) | 4.4 (-8.2--0.6) | 1.38 | 0.241 |
|  | Antihypertensivedrugs | 7 (23.3) | 28 (24.8) | 1.5 (-18.6-15.6) | 0.03 | 0.870 |
|  | NSAIDs | 0 (0.0) | 7 (6.2) | 6.2 (-10.7--1.8) | 0.85 | 0.357 |
|  | Antiplatelet | 20 (66.7) | 54 (47.8) | -18.9 (-0.3-38.1) | 3.38 | 0.066 |
|  | StomachProtect | 5 (16.7) | 54 (47.8) | 31.1 (-47.3--14.9) | 9.47 | 0.002 |
|  | Lipidlowering | 10 (33.3) | 64 (57.1) | 23.8 (-43.0--4.6) | 5.38 | 0.020 |
|  | Diuretics | 1 (3.3) | 11 (9.8) | 6.5 (-14.9-1.9) | 0.59 | 0.444 |
| Five Coagulation Items | PT | 11.01 ± 0.52 | 11.23 ± 0.77 | 0.22 (-0.07-0.51) | 1.48 | 0.140 |
|  | INR | 0.97 ± 0.05 | 0.98 ± 0.07 | 0.02 (0.00-0.04) | 1.53 | 0.130 |
|  | APTT | 25.98 ± 1.65 | 27.24 ± 2.12 | 1.26 (0.43-2.09) | 3.02 | 0.003 |
|  | TT | 17.38 ± 0.89 | 18.27 ± 1.35 | 0.89 (0.37-1.40) | 3.40 | 0.001 |
|  | FIB | 2.61 ± 0.69 | 2.75 ± 0.80 | 0.12 (-0.20-0.44) | 0.72 | 0.474 |
|  | D2 | 0.20 (0.12, 0.33) | 0.32 (0.20, 0.92) | 0.13 (0.05-0.28) | -3.23 | 0.001 |
| Echocardio-graphy | EF | 60.20 ± 3.08 | 57.71 ± 4.45 | -2.51 (-4.23--0.79) | -2.88 | 0.005 |
|  | LAD | 3.65 ± 0.41 | 4.35 ± 0.62 | 0.67 (0.48-0.85) | 7.02 | ＜0.001 |
|  | LVEDD | 4.93 ± 0.40 | 4.96 ± 0.48 | 0.00 (-0.19-0.19) | 0.00 | 0.998 |
| Liver Function | ALT | 19.9 (13.9, 26.1) | 16 (13.0, 22.7) | -2.3 (-5.8-1.2) | -1.24 | 0.214 |
|  | AST | 21.4 (17.7, 23.3) | 19.1 (15.1, 22.3) | -1.9 (-3.9-0.4) | -1.65 | 0.100 |
|  | ALP | 67.5 (56.3, 78.8) | 68.9 (58.0, 83.4) | 1.6 (-5.9-9.5) | -0.47 | 0.639 |
|  | GGT | 25.5 (18.7, 55.3) | 25.0 (18.1, 46.8) | -1.0 (-6.7-4.5) | -0.30 | 0.760 |
|  | LDH | 182 (155, 216) | 188 (166, 224) | 11 (-6-27) | -1.26 | 0.206 |
|  | TBIL | 11.8 (8.8,14.7) | 10.7 (8.7,16.3) | 0.2 (-1.5-1.9) | -0.25 | 0.800 |
|  | DBIL | 2.99 ± 1.16 | 3.62 ± 2.44 | 0.63 (0.01-1.25) | 2.02 | 0.046 |
|  | CHE | 8.58 ± 2.70 | 7.33 ± 1.77 | -1.25 (-2.06--0.43) | -3.03 | 0.003 |
|  | TP | 65.52 ± 4.78 | 65.33 ± 5.07 | -0.19 (-2.22-1.85) | -0.18 | 0.856 |
|  | ALB | 40.65 ± 2.10 | 39.84 ± 2.93 | -0.81 (-1.94-0.32) | -1.42 | 0.159 |
|  | GLO | 24.87 ± 3.89 | 25.49 ± 3.86 | 0.62 (-0.95-2.19) | 0.78 | 0.436 |
|  | AGratio | 1.67 ± 0.26 | 1.60 ± 0.25 | -0.07 (-0.18-0.03) | -1.42 | 0.159 |
|  | TBA | 4.5 (3.0, 7.6) | 3.6 (2.1, 5.4) | -1.1 (-2.2--0.1) | -2.06 | 0.039 |
|  | LAP | 46.6 (43.8, 50.7) | 47.5 (42.8, 56.7) | 0.8 (-2.4-4.7) | -0.48 | 0.634 |
|  | ADA | 10.25 ± 3.37 | 11.81 ± 3.88 | 1.57 (0.03-3.10) | 2.02 | 0.046 |
| Kidney Function | GLU | 4.75 (4.51, 5.06) | 4.96 (4.56, 5.66) | 0.22 (-0.02-0.51) | -1.77 | 0.077 |
|  | UREA | 4.97 ± 1.46 | 5.84 ± 1.75 | 0.87 (0.18-1.56) | 2.50 | 0.013 |
|  | CREA | 61.70 ± 12.65 | 68.63 ± 18.67 | 6.93 (-0.22-14.08) | 1.92 | 0.057 |
|  | URIC | 344.10 ± 87.23 | 359.31 ± 101.75 | 15.21 (-24.96-55.38) | 0.75 | 0.455 |
|  | TCO2 | 24.89 ± 1.25 | 25.26 ± 2.22 | 0.37 (-0.25-0.98) | 1.19 | 0.238 |
|  | eGFR | 111.97 ± 23.83 | 102.95 ± 24.17 | -9.02 (-18.80-0.77) | -1.82 | 0.071 |
| Blood Lipids | TRIG | 1.29 (1.05,1.61) | 1.14 (0.87,1.63) | -0.12 (-0.31-0.09) | -1.15 | 0.251 |
|  | CHOL | 4.32 ± 0.91 | 4.31 ± 0.84 | -0.01 (-0.35-0.34) | -0.03 | 0.976 |
|  | HDLC | 1.22 ± 0.44 | 1.21 ± 0.36 | -0.02 (-0.17-0.14) | -0.20 | 0.842 |
|  | LDLC | 2.45 ± 0.71 | 2.47 ± 0.73 | 0.02 (-0.27-0.32) | 0.14 | 0.888 |
|  | APOAⅠ | 1.06 ± 0.23 | 1.01 ± 0.21 | -0.05 (-0.14-0.04) | -1.13 | 0.260 |
|  | APOB | 0.70 ± 0.19 | 0.73 ± 0.19 | 0.03 (-0.05-0.11) | 0.75 | 0.454 |
| Electrolytes | Ca | 2.34 ± 0.09 | 2.31 ± 0.12 | -0.04 (-0.08-0.01) | -1.52 | 0.131 |
|  | PHOS | 1.05 ± 0.14 | 1.01 ± 0.16 | -0.04 (-0.10-0.02) | -1.31 | 0.191 |
|  | K | 3.90 ± 0.36 | 3.93 ± 0.31 | 0.03 (-0.10-0.16) | 0.48 | 0.630 |
|  | Na | 140.46 ± 1.32 | 141.41 ± 2.39 | 0.95 (0.30-1.61) | 2.89 | 0.005 |
|  | Cl | 105.62 ± 2.14 | 104.96 ± 2.75 | -0.66 (-1.73-0.41) | -1.22 | 0.225 |
| White Blood Cells | WBC | 5.65 ± 1.66 | 6.60 ± 2.43 | 0.95 (0.19-1.71) | 2.50 | 0.015 |
|  | NEUTP | 60.24 ± 9.36 | 62.97 ± 12.4 | 2.73 (-2.07-7.54) | 1.12 | 0.263 |
|  | LYMPHP | 30.76 ± 8.68 | 27.54 ± 10.78 | -3.22 (-7.43-0.99) | -1.51 | 0.133 |
|  | MONOP | 6.34 ± 1.48 | 6.90 ± 2.17 | 0.56 (-0.27-1.39) | 1.33 | 0.186 |
|  | EOSP | 2.24 ± 1.50 | 2.13 ± 2.00 | -0.11 (-0.88-0.67) | -0.27 | 0.788 |
|  | BASOP | 0.42 ± 0.19 | 0.45 ± 0.30 | 0.03 (-0.08-0.15) | 0.59 | 0.553 |
|  | NEUT# | 3.45 ± 1.30 | 4.36 ± 2.39 | 0.90 (0.25-1.55) | 2.76 | 0.007 |
|  | LYMPH# | 1.67 ± 0.54 | 1.65 ± 0.66 | -0.03 (-0.29-0.23) | -0.20 | 0.841 |
|  | MONO# | 0.36 ± 0.15 | 0.45 ± 0.20 | 0.09 (0.01-0.17) | 2.27 | 0.025 |
|  | EOS# | 0.11 (0.05, 0.20) | 0.1 (0.05, 0.16) | -0.01 (-0.04-0.02) | -0.65 | 0.519 |
|  | BASO# | 0.02 ± 0.01 | 0.03 ± 0.02 | 0.00 (0.00-0.01) | 2.08 | 0.041 |
| Red Blood Cells | RBC | 4.35 ± 0.40 | 4.49 ± 0.57 | 0.15 (-0.07-0.36) | 1.32 | 0.188 |
|  | HGB | 134.43 ± 14.65 | 141.73 ± 17.25 | 7.29 (0.49-14.09) | 2.12 | 0.036 |
|  | HCT | 39.11 ± 3.48 | 41.34 ± 4.82 | 2.23 (0.37-4.09) | 2.37 | 0.019 |
|  | MCV | 90.08 ± 4.31 | 92.24 ± 4.95 | 2.15 (0.20-4.11) | 2.17 | 0.031 |
|  | MCH | 30.92 ± 1.94 | 31.63 ± 1.92 | 0.70 (-0.08-1.48) | 1.78 | 0.077 |
|  | MCHC | 343.23 ± 10.62 | 342.84 ± 10.96 | -0.39 (-4.81-4.03) | -0.18 | 0.861 |
|  | RDW | 12.8 (12.3, 13.2) | 12.6 (12.2, 13.1) | -0.1 (-0.3-0.2) | -0.48 | 0.634 |
| Platelets | PLT | 208.47 ± 69.77 | 180.82 ± 59.02 | -27.64 (-52.57--2.72) | -2.19 | 0.030 |
|  | PCT | 0.21 ± 0.06 | 0.20 ± 0.05 | -0.01 (-0.03-0.01) | -1.18 | 0.240 |
|  | PDW | 16.2 (15.7, 16.6) | 16.2 (16.0, 16.4) | 0.0 (-0.2-0.2) | -0.09 | 0.925 |
|  | MPV | 10.75 ± 1.83 | 10.96 ± 1.40 | 0.21 (-0.4-0.82) | 0.69 | 0.494 |

NOTE. Day, the number of days in hospital; HR, heart rate; PT, prothrombin time; INR, international normalized ratio; APTT, activated partial thromboplastin time; TT, thrombin time; FIB, fibrinogen quantification; BNP, brain natriuretic peptide; TNT, cardiac troponin T; CK-MB, creatine kinase isoenzyme MB; EF, ejection fraction; LAD, left atrium diameter; LVEDD, left ventricular end-diastolic diameter; CRP, C reactive protein; Smoking, smoking history; Drinking, drinking history; Family, family genetic history; Bleeding, bleeding history; Embolism, history of the thromboembolic disease; PAH, pulmonary hypertension; Pulmonary, pulmonary disease; Blood, blood disease; HFNYHA, cardiac insufficiency; CAD, coronary artery disease; Heart, other heart diseases, such as arrhythmia or mild heart valve disease; Anticoagulants, injectable anticoagulants; ApoA 1, apolipoprotein A1; ApoB, apolipoprotein B.
