# Supplementary material for: Comprehensive metabolomic characterization of atrial fibrillation
Source: Front Cardiovasc Med. 2022 Aug 8;9:911845. doi: 10.3389/fcvm.2022.911845 (PMC9393302; doi:10.3389/fcvm.2022.911845)
Supplement: Supplementary file 2 [file Table_2.DOCX]

**Supplemental Table 2.** Baseline Characteristics of Discovery Phase AFs

|  | Variable | Fir-AF (n=22) | Par-AF (n=33) | Per-AF (n=26) | Car-AF (n=32) | p Value for Trend |  |
| --- | --- | --- | --- | --- | --- | --- | --- |
|  |  |  |  |  |  |  |  |
| Demographics | Male | 16 (76.2) | 17 (51.5) | 21 (77.8) | 19 (59.4) | 0.105 |  |
|  | Day | 7.48 ± 3.17 | 7.33 ± 3.18 | 6.30 ± 2.92 | 15.34 ± 7.34 | ＜0.001 |  |
|  | Age | 53.81 ± 11.67 | 62.36 ± 8.40 | 68.33 ± 8.40 | 74.34 ± 8.96 | ＜0.001 |  |
|  | Weight | 72.94 ± 12.85 | 66.05 ± 11.29 | 73.70 ± 11.15 | 68.22 ± 11.50 | 0.043 |  |
|  | Height | 1.73 ± 0.09 | 1.63 ± 0.09 | 1.70 ± 0.05 | 1.67 ± 0.07 | ＜0.001 |  |
|  | BMI | 24.33 ± 3.39 | 24.78 ± 3.38 | 25.59 ± 3.67 | 23.95 ± 2.83 | 0.338 |  |
|  | BSA | 1.95 ± 0.19 | 1.80 ± 0.18 | 1.94 ± 0.15 | 1.86 ± 0.18 | 0.006 |  |
|  | SBP | 135.33 ± 20.1 | 135.52 ± 18.51 | 133.41 ± 17.51 | 142.34 ± 24.03 | 0.342 |  |
|  | DBP | 84.43 ± 10.56 | 80.52 ± 12.44 | 82.96 ± 11.91 | 83.22 ± 14.34 | 0.695 |  |
|  | HR | 69.38 ± 9.87 | 71.88 ± 14.54 | 77.67 ± 15.17 | 85.00 ± 21.04 | 0.003 |  |
|  | CHA2DS2-VASc | 0.95 ± 0.80 | 1.52 ± 1.12 | 2.44 ± 1.48 | 4.97 ± 1.15 | ＜0.001 |  |
|  | HAS-BLED | 0.33 ± 0.58 | 0.64 ± 0.65 | 1.04 ± 0.94 | 2.06 ± 0.88 | ＜0.001 |  |
|  | Crcl | 106.86 ± 34.29 | 87.54 ± 37.56 | 88.19 ± 30.66 | 79.99 ± 30.09 | 0.078 |  |
| Five Coagulation Items | PT | 10.95 ± 0.63 | 10.93 ± 0.52 | 11.16 ± 0.77 | 11.78 ± 0.79 | ＜0.001 |  |
|  | INR | 0.96 ± 0.06 | 0.96 ± 0.05 | 0.97 ± 0.07 | 1.03 ± 0.07 | ＜0.001 |  |
|  | APTT | 27.76 ± 2.54 | 27.32 ± 2.19 | 26.92 ± 2.07 | 27.07 ± 1.81 | 0.549 |  |
|  | TT | 18.28 ± 1.20 | 18.3 ± 0.93 | 18.55 ± 0.98 | 17.98 ± 1.95 | 0.463 |  |
|  | FIB | 2.79 ± 0.84 | 2.45 ± 0.40 | 2.59 ± 0.62 | 3.18 ± 1.01 | 0.003 |  |
|  | D-Dimer | 0.13 (0.20, 0.27) | 0.17 (0.22, 0.39) | 0.23 (0.30, 0.54) | 0.57 (1.06, 1.84) | ＜0.001 |  |
| Cardiac  Markers | BNP | 21.5 (36.9, 59.8) | 29.5 (63.6, 116.3) | 90.58 (260.5, 325.3) | 153.5 (237.5, 366.3) | ＜0.001 |  |
|  | TNT | 0.006 (0.007, 0.009) | 0.006 (0.009, 0.011) | 0.008 (0.010, 0.015) | 0.011 (0.014, 0.028) | ＜0.001 |  |
|  | CKMB | 6.3 (9.0, 17.3) | 7.0 (9.0, 10.0) | 7.8 (10.0, 14.3) | 8.8 (13.0, 14.8) | 0.011 |  |
|  | EF | 57.5 (60.0, 60.8) | 57.5 (59.0, 61.0) | 54.8 (57.0, 59.3) | 48.3 (57.0, 59.5) | ＜0.001 |  |
|  | LAD | 4.10 ± 0.52 | 3.97 ± 0.35 | 4.61 ± 0.56 | 4.73 ± 0.73 | ＜0.001 |  |
|  | LVEDD | 5.12 ± 0.36 | 4.96 ± 0.38 | 5.01 ± 0.63 | 4.85 ± 0.50 | 0.275 |  |
|  | HbAlc | 5.2 (5.8, 6.0) | 5.4 (5.6, 6.3) | 5.7 (6.1, 6.8) | 5.4 (5.9, 6.4) | 0.098 |  |
|  | CRP | 2.15 (2.75, 4.38) | 2.40 (3.10, 3.75) | 2.68 (3.20, 4.5) | 2.48 (3.40, 7.88) | ＜0.001 |  |
|  | Smoking | 4 (19.0) | 5 (15.2) | 11 (40.7) | 8 (25.0) | 0.127 |  |
|  | Drinking | 3 (14.3) | 5 (15.2) | 8 (29.6) | 5 (15.6) | 0.411 |  |
|  | Family | 2 (9.5) | 2 (6.1) | 1 (3.7) | 3 (9.4) | 0.794 |  |
| Comorbidity | Bleeding | 0 (0.0) | 1 (3.0) | 1 (3.7) | 10 (31.3) | ＜0.001 |  |
|  | Embolism | 1 (4.8) | 1 (3.0) | 8 (29.6) | 32 (100.0) | ＜0.001 |  |
|  | T2DM | 3 (14.3) | 1 (3.0) | 4 (14.8) | 10 (31.3) | 0.017 |  |
|  | Hyperlipidemia | 7 (33.3) | 2 (6.1) | 4 (14.8) | 5 (15.6) | 0.076 |  |
|  | Hepatopathy | 1 (4.8) | 0 (0.0) | 2 (7.4) | 8 (25.0) | 0.004 |  |
|  | Nephropathy | 3 (14.3) | 0 (0.0) | 1 (3.7) | 7 (21.9) | 0.007 |  |
|  | PAH | 2 (9.5) | 9 (9.1) | 3 (11.1) | 7 (21.9) | 0.471 |  |
|  | Pulmonary | 6 (28.6) | 8 (24.2) | 6 (22.2) | 26 (81.3) | ＜0.001 |  |
|  | Blood | 3 (14.3) | 5 (15.2) | 2 (7.4) | 18 (56.3) | ＜0.001 |  |
|  | HFNYHA | 1 (4.8) | 0 (0.0) | 5 (18.5) | 7 (21.9) | 0.007 |  |
|  | Hypertension | 8 (38.1) | 13 (39.4) | 12 (44.4) | 21 (65.6) | 0.115 |  |
|  | CAD | 0 (0.0) | 2 (6.1) | 2 (7.4) | 8 (25.0) | 0.020 |  |
|  | Heart | 10 (47.6) | 11 (33.3) | 8 (29.6) | 9 (28.1) | 0.481 |  |
| Medication | Anticoagulants | 3 (14.3) | 7 (21.2) | 7 (25.9) | 17 (53.1) | 0.007 |  |
|  | Amiodarone | 11 (52.4) | 12 (36.4) | 5 (18.5) | 1 (3.1) | ＜0.001 |  |
|  | Propafenone | 7 (33.3) | 15 (45.5) | 0 (0.0) | 0 (0.0) | ＜0.001 |  |
|  | Digoxin | 0 (0.0) | 0 (0.0) | 4 (14.8) | 1 (3.1) | 0.018 |  |
|  | Antihypertensive | 0 (0.0) | 0 (0.0) | 4 (14.8) | 1 (3.1) | 0.018 |  |
|  | NSAIDs | 1 (4.8) | 0 (0.0) | 0 (0.0) | 6 (18.8) | 0.004 |  |
|  | Antiplatelet | 6 (28.6) | 11 (33.3) | 11 (40.7) | 26 (81.3) | ＜0.001 |  |
|  | Stomach-protect | 10 (47.6) | 14 (42.4) | 13 (48.1) | 17 (53.1) | 0.862 |  |
|  | Lipid-lowering | 9 (42.9) | 15 (46.9) | 10 (37.0) | 30 (93.8) | ＜0.001 |  |
|  | Diuretics | 1 (4.8) | 0 (0.0) | 5 (19.2) | 5 (15.6) | 0.024 |  |
| Liver Function | ALT | 12.98 (16.55,22.53) | 13.40 (15.60,20.55) | 13.33 (17.25,34.03) | 6.58 (10.95,19.78) | 0.420 |  |
|  | AST | 18.59 ± 3.92 | 18.48 ± 5.44 | 19.64 ± 5.78 | 24.81 ± 16.15 | 0.192 |  |
|  | AKP | 52.8 (70.6, 75.7) | 62.7 (77.6, 88.8) | 61.3 (67.4, 79.6) | 62.3 (77.4, 103.3) | 0.535 |  |
|  | GGT | 22.18 (25.15, 59.58) | 17.30 (21.70, 31.45) | 21.38 (29.15, 52.73) | 17.83 (28.40, 49.83) | 0.072 |  |
|  | LDH | 187.76 ± 29.21 | 173.21 ± 31.43 | 198.11 ± 46.90 | 246.69 ± 80.21 | ＜0.001 |  |
|  | TBIL | 7.83 (9.80, 16.7) | 9.30 (10.60, 13.65) | 9.90 (13.65, 20.3) | 9.35 (13.00, 22.30) | 0.008 |  |
|  | DBIL | 2.10 (2.55, 3.45) | 2.00 (2.80, 3.85) | 2.15 (3.25, 5.33) | 2.63 (3.00, 6.63) | ＜0.001 |  |
|  | CHE | 8.00 ± 1.33 | 7.81 ± 1.81 | 7.11 ± 1.42 | 6.58 ± 1.99 | 0.007 |  |
|  | TP | 67.05 ± 3.76 | 65.27 ± 4.80 | 65.68 ± 5.10 | 63.96 ± 5.83 | 0.180 |  |
|  | ALB | 41.66 ± 2.18 | 40.38 ± 1.95 | 39.74 ± 3.25 | 38.17 ± 3.12 | ＜0.001 |  |
|  | GLO | 25.40 ± 3.29 | 24.89 ± 4.14 | 25.94 ± 3.50 | 25.79 ± 4.28 | 0.718 |  |
|  | AG-ratio | 1.67 ± 0.24 | 1.66 ± 0.25 | 1.56 ± 0.26 | 1.51 ± 0.24 | 0.046 |  |
|  | TBA | 2.15 (2.60, 4.18) | 1.65 (2.10, 4.60) | 1.58 (3.85, 5.45) | 3.88 (5.35, 7.60) | 0.407 |  |
|  | LAP | 58.59 ± 15.71 | 46.29 ± 8.57 | 53.40 ± 12.41 | 53.98 ± 35.64 | 0.209 |  |
|  | ADA | 11.27 ± 2.22 | 10.25 ± 3.42 | 12.07 ± 3.13 | 13.57 ± 5.00 | 0.018 |  |
| Kidney Function | GLU | 4.3 (4.7, 5.3) | 4.5 (4.9, 5.0) | 4.5 (5.7, 6.4) | 4.6 (5.5, 6.1) | ＜0.001 |  |
|  | UREA | 5.47 ± 1.67 | 5.45 ± 1.25 | 6.23 ± 1.56 | 6.17 ± 2.25 | 0.172 |  |
|  | CREA | 70.24 ± 18.26 | 62.61 ± 13.58 | 72.19 ± 16.33 | 70.79 ± 23.96 | 0.172 |  |
|  | URIC | 382.10 ± 86.77 | 339.58 ± 97.65 | 391.78 ± 87.76 | 337.31 ± 118.58 | 0.086 |  |
|  | TCO2 | 26.27 ± 1.23 | 25.55 ± 1.66 | 26.03 ± 2.07 | 23.65 ± 2.54 | ＜0.001 |  |
|  | eGFR | 106.86 ± 21.24 | 108.20 ± 19.13 | 99.20 ± 25.03 | 98.13 ± 28.98 | 0.260 |  |
| Blood Lipids | TRIG | 1.47 ± 0.65 | 1.42 ± 1.33 | 1.42 ± 0.62 | 1.16 ± 0.61 | 0.543 |  |
|  | CHOL | 4.68 ± 0.86 | 4.30 ± 0.72 | 4.32 ± 0.74 | 4.08 ± 0.96 | 0.086 |  |
|  | HDL | 1.21 ± 0.36 | 1.25 ± 0.34 | 1.18 ± 0.32 | 1.18 ± 0.42 | 0.873 |  |
|  | LDL | 2.79 ± 0.67 | 2.42 ± 0.68 | 2.50 ± 0.66 | 2.28 ± 0.84 | 0.095 |  |
|  | ApoA 1 | 1.00 ± 0.23 | 1.05 ± 0.16 | 1.03 ± 0.20 | 0.96 ± 0.25 | 0.367 |  |
|  | ApoB | 0.80 ± 0.17 | 0.70 ± 0.19 | 0.74 ± 0.20 | 0.69 ± 0.19 | 0.174 |  |
| Electrolytes | Ca | 2.33 ± 0.09 | 2.32 ± 0.10 | 2.32 ± 0.11 | 2.26 ± 0.16 | 0.268 |  |
|  | PHOS | 1.01 ± 0.13 | 1.07 ± 0.15 | 0.99 ± 0.13 | 0.97 ± 0.18 | 0.059 |  |
|  | K | 3.97 ± 0.33 | 3.91 ± 0.19 | 3.95 ± 0.36 | 3.92 ± 0.35 | 0.859 |  |
|  | Na | 141.95 ± 1.76 | 141.67 ± 1.82 | 141.49 ± 1.97 | 140.72 ± 3.33 | 0.383 |  |
|  | Cl | 104.92 ± 2.37 | 105.63 ± 2.19 | 104.1 ± 2.78 | 105.03 ± 3.34 | 0.199 |  |
| White Blood Cells Items | WBC | 5.89 ± 1.85 | 5.52 ± 1.62 | 5.83 ± 1.67 | 8.83 ± 2.63 | ＜0.001 |  |
|  | NEUTP | 57.97 ± 8.83 | 57.16 ± 7.96 | 58.05 ± 6.69 | 76.41 ± 11.90 | ＜0.001 |  |
|  | LYMPHP | 31.64 ± 8.48 | 32.92 ± 7.26 | 31.82 ± 5.21 | 15.69 ± 9.62 | ＜0.001 |  |
|  | MONOP | 7.48 ± 3.02 | 6.77 ± 1.43 | 7.09 ± 2.15 | 6.49 ± 2.17 | 0.406 |  |
|  | EOSP | 1.20 (1.85, 2.55) | 1.55 (2.10, 2.85) | 1.30 (1.85, 2.50) | 0.23 (0.85, 1.85) | ＜0.001 |  |
|  | BASOP | 0.45 ± 0.33 | 0.55 ± 0.33 | 0.54 ± 0.27 | 0.28 ± 0.21 | 0.001 |  |
|  | NEUT# | 3.46 ± 1.35 | 3.18 ± 1.12 | 3.42 ± 1.18 | 6.95 ± 2.70 | ＜0.001 |  |
|  | LYMPH# | 1.40 (1.75, 2.38) | 1.30 (1.80, 2.10) | 1.40 (1.70, 1.90) | 1.00 (1.05, 1.53) | ＜0.001 |  |
|  | MONO# | 0.43 ± 0.17 | 0.38 ± 0.14 | 0.40 ± 0.15 | 0.56 ± 0.26 | 0.011 |  |
|  | EOS# | 0.14 ± 0.12 | 0.15 ± 0.13 | 0.14 ± 0.11 | 0.07 ± 0.09 | 0.028 |  |
|  | BASO# | 0.01 (0.02, 0.05) | 0.02 (0.02, 0.04) | 0.02 (0.03, 0.04) | 0.01 (0.02, 0.04) | 0.048 |  |
| Red Blood Cells Items | RBC | 4.75 ± 0.59 | 4.44 ± 0.48 | 4.56 ± 0.40 | 4.32 ± 0.70 | 0.041 |  |
|  | HGB | 145.24 ± 15.10 | 138.30 ± 17.32 | 147.70 ± 11.74 | 137.91 ± 20.88 | 0.071 |  |
|  | HCT | 42.67 ± 3.82 | 40.40 ± 4.74 | 42.94 ± 3.27 | 40.08 ± 6.05 | 0.030 |  |
|  | MCV | 90.34 ± 6.79 | 90.94 ± 4.91 | 94.39 ± 3.60 | 93.01 ± 3.74 | 0.009 |  |
|  | MCH | 30.75 ± 2.47 | 31.13 ± 2.15 | 32.47 ± 1.40 | 31.99 ± 1.18 | 0.004 |  |
|  | MCHC | 340.19 ± 10.55 | 342.18 ± 12.62 | 344.33 ± 12.14 | 344 ± 8.10 | 0.536 |  |
|  | RDW | 12.71 ± 0.77 | 17.68 ± 29.69 | 16.54 ± 20.09 | 12.96 ± 0.54 | 0.677 |  |
| Platelets Items | PLT | 180 ± 49.08 | 184.91 ± 55.79 | 163.75 ± 62.6 | 191.56 ± 64.35 | 0.326 |  |
|  | PCT | 0.20 ± 0.04 | 0.21 ± 0.04 | 0.19 ± 0.05 | 0.19 ± 0.06 | 0.557 |  |
|  | PDW | 16.02 ± 0.73 | 15.75 ± 1.60 | 15.86 ± 1.49 | 16.21 ± 0.36 | 0.226 |  |
|  | MPV | 11.03 ± 1.32 | 11.15 ± 1.35 | 11.46 ± 1.58 | 10.29 ± 1.13 | 0.009 |  |
