# Supplementary material for: Comprehensive metabolomic characterization of atrial fibrillation
Source: Front Cardiovasc Med. 2022 Aug 8;9:911845. doi: 10.3389/fcvm.2022.911845 (PMC9393302; doi:10.3389/fcvm.2022.911845)
Supplement: Supplementary file 3 [file Table_3.DOCX]

**Supplemental Table 3.** Baseline Characteristics of Validation Phase Participants

|  | Variable | Controls  (n=30) | All-AFs plus Car-AF  (n=104) | Effect size;  95%CI | t/Z/χ2-value | P |
| --- | --- | --- | --- | --- | --- | --- |
| Demographics | Sex | 17 (43.33) | 37 (64.42) | 0.21 (-0.41--0.01) | 4.30 | 0.038 |
|  | Age | 56.53 ± 5.75 | 64.22 ± 12.28 | -7.69 (-10.86--4.52) | -4.81 | ＜0.001 |
|  | Weight | 61.41 ± 8.08 | 69.48 ± 12.31 | -8.07 (-11.96--4.19) | -4.14 | ＜0.001 |
|  | Height | 1.64 ± 0.08 | 1.68 ± 0.08 | -0.04 (-0.07-0.00) | -2.18 | 0.031 |
|  | BMI | 22.69 ± 1.40 | 24.58 ± 3.55 | -1.89 (-2.78--1.00) | -4.22 | ＜0.001 |
|  | BSA | 1.75 ± 0.14 | 1.88 ± 0.19 | -0.12 (-0.20--0.05) | -3.30 | 0.001 |
|  | SBP | 121.47 ± 10.10 | 135.56 ± 19.97 | -14.09 (-19.43--8.75) | -5.24 | ＜0.001 |
|  | DBP | 77.5 (67.3, 81.0) | 83.0 (76.0, 93.0) | -8.0 (-13.0--3.0) | -3.43 | ＜0.001 |
|  | HbAlc | 5.7 (5.3, 6.0) | 5.9 (5.5, 6.3) | -0.3 (-0.5-0.0) | -2.14 | 0.032 |
| Biochemical Items | ALT | 14.5 (11.7, 20.1) | 16.6 (13.0, 23.9) | -2.0 (-4.6-0.4) | -1.66 | 0.098 |
|  | AST | 18.0 (16.4, 21.2) | 19.1 (15.6, 22.3) | -0.1 (-1.9-1.6) | -0.11 | 0.918 |
|  | AKP | 72.7 (57.3, 80.3) | 70.6 (59.8, 84.9) | 1.2 (-5.7-7.7) | -0.32 | 0.753 |
|  | GGT | 21.5 (16.5, 28.7) | 25.6 (18.5, 50.4) | -4.7 (-10.2--0.6) | -2.30 | 0.021 |
|  | LDH | 175.0 (152.8, 186.5) | 182.5 (164.0, 220.0) | -17.0 (-33.0--4.0) | -2.63 | 0.008 |
|  | TBIL | 10.5 (7.8, 12.2) | 10.6 (8.5, 14.4) | -1.8 (-3.8-0.0) | -1.98 | 0.048 |
|  | DBIL | 2.3 (2.0, 2.9) | 2.6 (2.0, 3.9) | -0.4 (-0.9-0.1) | -1.57 | 0.118 |
|  | CHE | 8.82 ± 1.74 | 7.24 ± 1.80 | 1.58 (0.79-2.37) | 3.96 | ＜0.001 |
|  | TP | 71.6 (69.9, 74.2) | 65.5 (62.5, 69.2) | 6.5 (4.7-8.3) | -5.65 | ＜0.001 |
|  | ALB | 43.86 ± 1.33 | 40.13 ± 2.61 | 3.73 (3.03-4.43) | 10.54 | ＜0.001 |
|  | GLO | 28.0 (26.0, 29.4) | 25.3 (23.1, 28.5) | 2.7 (1.4-4.1) | -3.57 | ＜0.001 |
|  | AGratio | 1.57 ± 0.15 | 1.60 ± 0.24 | -0.03 (-0.10-0.04) | -0.80 | 0.425 |
|  | TBA | 1.95 (1.23, 3.55) | 3.60 (2.08, 5.40) | -1.30 (-2.20--0.60) | -3.78 | ＜0.001 |
|  | LAP | 47.85 (46.05, 49.40) | 50.15 (44.05, 57.40) | -1.30 (-4.90-2.30) | -0.73 | 0.466 |
|  | GLU | 5.23 (4.90, 5.60) | 4.83 (4.49, 5.48) | 0.14 (-0.18-0.39) | -0.87 | 0.385 |
|  | UREA | 5.40 (4.63, 6.15) | 5.20 (4.48, 6.63) | -0.29 (-0.80-0.30) | -0.99 | 0.327 |
|  | CREA | 59.5 (53.8, 68.8) | 65.0 (55.0, 75.3) | -5.0 (-11.0-1.0) | -1.62 | 0.106 |
|  | URIC | 318.5 (291.8, 369.5) | 349.0 (285.0, 432.3) | -35.0 (-68.0--2.0) | -2.04 | 0.041 |
|  | TCO2 | 25.83 ± 1.54 | 25.50 ± 2.08 | 0.33 (-0.48-1.14) | 0.81 | 0.420 |
|  | TRIG | 0.88 (0.71, 1.03) | 1.10 (0.84, 1.73) | -0.26 (-0.47--0.09) | -3.04 | 0.002 |
|  | CHOL | 4.93 ± 0.87 | 4.26 ± 0.77 | 0.68 (0.35-1.00) | 4.09 | ＜0.001 |
|  | HDLC | 1.60 (1.37,1.81) | 1.13 (0.98,1.41) | 0.47 (0.32-0.63) | -5.06 | ＜0.001 |
|  | LDLC | 2.68 ± 0.60 | 2.42 ± 0.69 | 0.26 (-0.01-0.54) | 1.89 | 0.061 |
|  | eGFR | 104.3 (99.1, 111.9) | 107.2 (90.9, 121.2) | 2.0 (-7.1-10.3) | -0.38 | 0.705 |
| Blood Items | WBC | 5.2 (4.4, 6.0) | 5.7 (4.6, 7.0) | -0.7 (-1.4--0.1) | -2.30 | 0.021 |
|  | NEUTP | 57.93 ± 8.29 | 62.49 ± 10.68 | -4.56 (-8.74--0.38) | -2.16 | 0.033 |
|  | LYMPHP | 33.70 ± 8.04 | 28.13 ± 9.43 | 5.57 (1.82-9.32) | 2.94 | 0.004 |
|  | MONOP | 5.30 (4.83, 6.18) | 6.30 (5.58, 7.93) | -1.00 (-1.70--0.50) | -3.57 | ＜0.001 |
|  | EOSP | 2.40 (1.60, 2.98) | 1.70 (1.10, 2.65) | 0.40 (-0.10-1.00) | -1.58 | 0.115 |
|  | BASOP | 0.5 (0.3, 0.6) | 0.4 (0.3, 0.6) | 0.0 (0.0-0.1) | -1.04 | 0.299 |
|  | NEUT# | 2.8 (2.5, 3.3) | 3.6 (2.7, 4.4) | -0.7 (-1.2--0.2) | -2.70 | 0.006 |
|  | LYMPH# | 1.65 (1.5, 1.8) | 1.6 (1.3, 2.1) | 0.1 (-0.1-0.3) | -1.12 | 0.263 |
|  | MONO# | 0.3 (0.2, 0.4) | 0.4 (0.3, 0.5) | -0.1 (-0.2--0.1) | -4.18 | ＜0.001 |
|  | EOS# | 0.13 (0.08, 0.16) | 0.13 (0.06, 0.18) | 0.00 (-0.03-0.03) | -0.13 | 0.895 |
|  | BASO# | 0.02 (0.02, 0.03) | 0.02 (0.02, 0.03) | 0.00 (0.00-0.01) | -0.22 | 0.830 |
|  | RBC | 4.64 (4.40, 4.82) | 4.51 (4.19, 4.92) | 0.12 (-0.05-0.29) | -1.40 | 0.163 |
|  | HGB | 140.03 ± 9.97 | 141.91 ± 17.49 | -1.88 (-6.85-3.09) | -0.75 | 0.454 |
|  | HCT | 41.76 ± 2.52 | 41.14 ± 4.84 | 0.61 (-0.70-1.93) | 0.93 | 0.356 |
|  | MCV | 90.95 (88.85, 93.55) | 91.45 (88.88, 94.40) | -0.70 (-2.30-0.90) | -0.88 | 0.383 |
|  | MCH | 30.8 (30.2, 31.6) | 31.8 (30.3, 32.7) | -1.1 (-1.7--0.5) | -3.20 | 0.001 |
|  | MCHC | 335.33 ± 6.85 | 344.83 ± 11.97 | -9.49 (-12.90--6.08) | -5.54 | ＜0.001 |
|  | RDW | 12.8 (12.4, 13.2) | 12.6 (12.2, 13.1) | 0.2 (-0.1-0.4) | -1.33 | 0.186 |
|  | PLT | 204.0 (188.3, 227.5) | 181.0 (139.8, 213.5) | 27.0 (7.0-46.0) | -2.62 | 0.008 |
