# Supplementary material for: Comprehensive metabolomic characterization of atrial fibrillation
Source: Front Cardiovasc Med. 2022 Aug 8;9:911845. doi: 10.3389/fcvm.2022.911845 (PMC9393302; doi:10.3389/fcvm.2022.911845)
Supplement: Supplementary file 4 [file Table_4.DOCX]

**Supplemental Table 4.** Baseline Characteristics of Validation Phase AFs

|  | Variable | Fir-AF  (n=17) | Par-AF  (n=36) | Per-AF  (n=29) | Car-AF  (n=22) | p Value for Trend |
| --- | --- | --- | --- | --- | --- | --- |
|  |  |  |  |  |  |  |
| Demographics | Male | 11 (64.7) | 20 (55.6) | 24 (82.8) | 12 (54.5) | 0.093 |
|  | Age | 54.29 ± 11.11 | 58.58 ± 10.60 | 67.90 ± 8.38 | 76.27 ± 7.70 | ＜0.001 |
|  | Weight | 71.47 ± 12.53 | 67.85 ± 11.83 | 72.18 ± 12.41 | 64.61 ± 12.56 | 0.242 |
|  | Height | 1.71 ± 0.08 | 1.65 ± 0.08 | 1.71 ± 0.08 | 1.66 ± 0.06 | 0.006 |
|  | BMI | 24.32 ± 3.53 | 24.97 ± 3.40 | 24.78 ± 3.86 | 23.23 ± 3.38 | 0.539 |
|  | BSA | 1.92 ± 0.19 | 1.84 ± 0.18 | 1.93 ± 0.18 | 1.81 ± 0.19 | 0.093 |
|  | SBP | 132.2 ± 18.7 | 133.5 ± 18.6 | 132.2 ± 15.8 | 146.0 ± 25.1 | 0.050 |
|  | DBP | 82.5 ± 10.9 | 83.3 ± 12.6 | 82.6 ± 11.6 | 85.0 ± 15.9 | 0.911 |
|  | HR | 71.0 (62.0, 78.0) | 71.0 (64.8, 75.3) | 77.0 (66.0, 86.0) | 62.0 (58.0, 95.0) | 0.155 |
| Comorbidity | Smoking | 2 (11.8) | 7 (19.4) | 11 (37.9) | 5 (22.7) | 0.179 |
|  | Drinking | 1 (5.9) | 5 (13.9) | 8 (27.6) | 3 (13.6) | 0.280 |
|  | Family | 1 (5.9) | 2 (5.6) | 3 (10.3) | 3 (13.6) | 0.735 |
|  | Bleeding | 0 (0.0) | 0 (0.0) | 0 (0.0) | 7 (31.8) | ＜0.001 |
|  | Thromboembolism | 1 (5.9) | 2 (5.6) | 7 (24.1) | 19 (86.4) | ＜0.001 |
|  | HFNYHA | 0 (0.0) | 0 (0.0) | 7 (25.0) | 0 (0.0) | ＜0.001 |
|  | Hypertension | 6 (35.3) | 13 (36.1) | 12 (41.4) | 15 (68.2) | 0.091 |
|  | T2DM | 3 (17.6) | 2 (5.6) | 5 (17.2) | 9 (40.9) | 0.010 |
|  | CAD | 0 (0.0) | 1 (2.8) | 3 (10.3) | 5 (22.7) | 0.037 |
|  | Hyperlipidemia | 6 (35.3) | 3 (8.6) | 4 (13.8) | 4 (18.2) | 0.128 |
|  | Heart | 6 (35.3) | 14 (38.9) | 10 (34.5) | 4 (18.2) | 0.419 |
|  | Thyroid | 0 (0.0) | 1 (2.8) | 1 (3.4) | 2 (9.1) | 0.581 |
|  | Pulmonaryhypertension | 1 (5.9) | 3 (8.3) | 4 (13.8) | 5 (22.7) | 0.384 |
|  | Blood | 1 (5.9) | 8 (22.2) | 5 (17.2) | 13 (59.1) | 0.001 |
|  | Cancer | 0 (0.0) | 1 (2.8) | 0 (0.0) | 1 (4.5) | 0.805 |
