# Supplementary material for: Comprehensive metabolomic characterization of atrial fibrillation
Source: Front Cardiovasc Med. 2022 Aug 8;9:911845. doi: 10.3389/fcvm.2022.911845 (PMC9393302; doi:10.3389/fcvm.2022.911845)
Supplement: Supplementary file 5 [file Table_5.DOCX]

**Supplemental Table 5.** Risk factor analysis of Sus-AF VS. All-AFs plus Car-AF and Control VS. Sus-AF in the discovery cohort

|  | Independent variables | OR | 95%CI | | P |
| --- | --- | --- | --- | --- | --- |
| Sus-AF VS All-AFs plus Car-AF | Na | 1.721 | 1.128 | 2.626 | 0.0118 |
|  | Day | 2.134 | 1.432 | 3.18 | 0.0002 |
|  | TT | 4.054 | 1.755 | 9.365 | 0.0011 |
|  | LAD | 25.573 | 3.798 | 172.172 | 0.0009 |
| Control VS Sus-AF | DBP | 1.189 | 1.076 | 1.314 | 0.0007 |
|  | ALB | 0.179 | 0.071 | 0.452 | 0.0003 |
|  | HCT | 0.568 | 0.393 | 0.822 | 0.0027 |
|  | Crcl | 1.079 | 1.029 | 1.133 | 0.0019 |
