# Supplementary material for: Comprehensive metabolomic characterization of atrial fibrillation
Source: Front Cardiovasc Med. 2022 Aug 8;9:911845. doi: 10.3389/fcvm.2022.911845 (PMC9393302; doi:10.3389/fcvm.2022.911845)
Supplement: Supplementary file 6 [file Table_6.DOCX]

**Supplemental Table 6.** The identified endogenous compounds in serum using GC/MS system

| Number | Query | Rt | m/z |
| --- | --- | --- | --- |
| 1 | 3-Hydroxypyridine | 4.600 | 152 |
| 2 | Hexanoic acid | 4.680 | 207 |
| 3 | Pyruvate | 4.775 | 174 |
| 4 | Lactate | 4.910 | 191 |
| 5 | 2-Hydroxyisobutyric acid | 4.960 | 131 |
| 6 | glycolic acid | 5.070 | 177 |
| 7 | Alanine | 5.380 | 116 |
| 8 | 2-Hydroxybutyric acid | 5.650 | 205 |
| 9 | Oxalic acid | 5.785 | 220 |
| 10 | 3-Hydroxyisobutyric acid | 5.970 | 177 |
| 11 | 3-Hydroxybutyric acid | 5.970 | 191 |
| 12 | 2-Hydroxy-3-methylbutyric acid | 6.030 | 219 |
| 13 | D-2-Aminobutyric acid | 6.095 | 130 |
| 14 | Dodecamethylpentasiloxane | 6.140 | 281 |
| 15 | Monomethylphosphate | 6.165 | 241 |
| 16 | Urea-3TMS | 6.210 | 261 |
| 17 | 2-keto-3-methylvaleric acid | 6.365 | 200 |
| 18 | Acetamide | 6.450 | 184 |
| 19 | Valine | 6.510 | 218 |
| 20 | 2,2-Dimethylsuccinic acid | 6.565 | 231 |
| 21 | r-GABA | 6.650 | 233 |
| 22 | Urea-2TMS | 6.670 | 189 |
| 23 | Serine-2TMS | 6.855 | 116 |
| 24 | 4-Pentenoic acid | 6.870 | 157 |
| 25 | Leucine | 6.985 | 158 |
| 26 | Phosphate | 7.020 | 314 |
| 27 | Isoleucine | 7.170 | 158 |
| 28 | glycerol-3TMS | 7.170 | 218 |
| 29 | Proline | 7.200 | 142 |
| 30 | Glycine-2TMS | 7.280 | 174 |
| 31 | glyceric acid | 7.470 | 292 |
| 32 | Fumarate | 7.535 | 245 |
| 33 | Nonanoic acid | 7.630 | 215 |
| 34 | 2,3-Dihydroxybutanoic acid | 7.660 | 292 |
| 35 | Serine-3TMS | 7.695 | 204 |
| 36 | Threonine | 7.900 | 218 |
| 37 | 3-Aminoisobutyric acid-3TMS | 7.960 | 174 |
| 38 | cystathionine-2TMS | 8.035 | 104 |
| 39 | homocysteine | 8.055 | 234 |
| 40 | Aspartate-2TMS | 8.120 | 160 |
| 41 | methylcysteine | 8.140 | 218 |
| 42 | Mannoic acid | 8.145 | 217 |
| 43 | beta-Alanine-3TMS | 8.165 | 248 |
| 44 | 2,5-Diaminovalerolactam | 8.375 | 243 |
| 45 | Aminomalonic acid | 8.480 | 218 |
| 46 | Malate-3TMS | 8.580 | 233 |
| 47 | Dihydrouracil | 8.665 | 243 |
| 48 | meso-Erythritol | 8.745 | 217 |
| 49 | Methionine | 8.795 | 176 |
| 50 | 1H-Indole-Acetic Acid | 8.795 | 202 |
| 51 | Aspartate-3TMS | 8.795 | 232 |
| 52 | Pyroglutamate-TMS | 8.825 | 156 |
| 53 | γ-Aminobutyric acid | 8.835 | 174 |
| 54 | Cysteine | 9.030 | 220 |
| 55 | Creatinine | 9.060 | 329 |
| 56 | L-Threonic acid | 9.080 | 292 |
| 57 | 2-Ketoglutaric Acid | 9.130 | 198 |
| 58 | Glutamine-4TMS | 9.285 | 227 |
| 59 | Glutamate-3TMS | 9.400 | 246 |
| 60 | Phenylalanine | 9.485 | 218 |
| 61 | lauric acid | 9.540 | 257 |
| 62 | Asparagine | 9.720 | 231 |
| 63 | Pyrophosphoric acid-4TMS | 9.730 | 451 |
| 64 | Taurine | 9.745 | 326 |
| 65 | Aminosuccinate-3TMS | 9.785 | 232 |
| 66 | Lyxose | 9.810 | 307 |
| 67 | Mannonic acid | 9.880 | 275 |
| 68 | Methyl tetradecanoate (ES) | 9.960 | 199 |
| 69 | Methyl galactoside | 10.165 | 193 |
| 70 | O-Phosphoethanolamine | 10.260 | 315 |
| 71 | Glycerol-2-phosphate | 10.265 | 299 |
| 72 | Glycerol-3-phosphate | 10.265 | 357 |
| 73 | Glutamine-3TMS | 10.295 | 156 |
| 74 | D-Galactose-5TMS | 10.465 | 204 |
| 75 | Hippuric Acid | 10.485 | 206 |
| 76 | Hypoxanthine | 10.490 | 265 |
| 77 | Ornithine | 10.565 | 142 |
| 78 | Citrate | 10.580 | 273 |
| 79 | Myristic-1,2-13C2 acid (IS) | 10.630 | 287 |
| 80 | Myristic Acid | 10.635 | 285 |
| 81 | 2-Deoxygalactose | 10.770 | 217 |
| 82 | Fructose | 10.910 | 307 |
| 83 | Ribitol | 10.990 | 319 |
| 84 | Lysine | 11.095 | 317 |
| 85 | Histidine | 11.115 | 154 |
| 86 | Glucose | 11.160 | 319 |
| 87 | Tyrosine | 11.195 | 218 |
| 88 | Glucitol | 11.230 | 319 |
| 89 | D-Trehalose | 11.325 | 361 |
| 90 | Anthranilic acid | 11.375 | 266 |
| 91 | Beta-Methylglucopyranoside | 11.415 | 217 |
| 92 | 9-Hexadecenoic acid | 11.540 | 311 |
| 93 | Xanthine | 11.600 | 353 |
| 94 | Palmitic acid | 11.610 | 313 |
| 95 | Uric acid | 12.005 | 441 |
| 96 | Myo-Inositol | 12.020 | 318 |
| 97 | Heptadecanoic acid | 12.090 | 327 |
| 98 | Arachidic acid | 12.195 | 369 |
| 99 | Linoleic acid | 12.415 | 337 |
| 100 | Oleic acid | 12.430 | 339 |
| 101 | Stearic acid | 12.530 | 341 |
| 102 | Tryptophan | 12.565 | 202 |
| 103 | Cystine | 12.875 | 218 |
| 104 | Arachidonic acid | 13.115 | 117 |
| 105 | Fructose-6-Phosphate | 13.155 | 315 |
| 106 | 11-Elcosaenoic acid | 13.285 | 367 |
| 107 | Myo-Inositol-2-phosphate | 13.500 | 318 |
| 108 | Uridine | 13.530 | 217 |
| 109 | 5-Hydroxytryptamine | 13.570 | 174 |
| 110 | Docosahexaenoic acid | 13.895 | 117 |
| 111 | cis-5,8,11-Eicosatrienoic acid | 13.895 | 117 |
| 112 | Monopalmitin | 13.985 | 371 |
| 113 | 1-Monooleoylglycerol | 14.700 | 397 |
| 114 | 1-Monostearin | 14.795 | 399 |
| 115 | Lactose | 14.925 | 361 |
| 116 | Alpha-Tocopherol | 17.145 | 502 |
| 117 | Cholesterol-TMS | 17.375 | 458 |
