# Supplementary material for: Comprehensive metabolomic characterization of atrial fibrillation
Source: Front Cardiovasc Med. 2022 Aug 8;9:911845. doi: 10.3389/fcvm.2022.911845 (PMC9393302; doi:10.3389/fcvm.2022.911845)
Supplement: Supplementary file 7 [file Table_7.DOCX]

**Supplemental Table 7.** The identified endogenous compounds in serum using LC/MS system

|  | Description | Adducts | m/z | Retention time (min) |
| --- | --- | --- | --- | --- |
| 1 | Acetoacetic acid | M+H | 103.0463 | 3.187 |
| 2 | L-Serine | M+H | 106.0502 | 4.008 |
| 3 | Histamine | M+H | 112.0879 | 4.990 |
| 4 | Creatinine | M+H | 114.0674 | 2.675 |
| 5 | Dihydrouracil | M+H | 115.0552 | 2.990 |
| 6 | L-Proline | M+H | 116.0716 | 4.977 |
| 7 | L-Valine | M+H | 118.0872 | 3.125 |
| 8 | Betaine | M+H | 118.0873 | 2.814 |
| 9 | Succinic acid | M+H | 119.0368 | 2.258 |
| 10 | L-Threonine | M+H | 120.0610 | 2.757 |
| 11 | L-Cysteine | M+H | 122.0283 | 4.915 |
| 12 | Niacinamide | M+H | 123.0565 | 1.423 |
| 13 | Nicotinic acid | M+H | 124.0400 | 1.196 |
| 14 | Taurine | M+H | 126.0302 | 3.169 |
| 15 | L-Pyroglutamic acid | M+H | 130.0509 | 3.973 |
| 16 | Agmatine | M+H | 131.0898 | 4.788 |
| 17 | Creatine | M+H | 132.0778 | 3.379 |
| 18 | L-Leucine | M+H | 132.1030 | 4.388 |
| 19 | L-Asparagine | M+H | 133.0655 | 0.726 |
| 20 | S-Methyl-L-cysteine | M+H | 136.0405 | 1.695 |
| 21 | Hypoxanthine | M+H | 137.0474 | 2.258 |
| 22 | 4-Hydroxybenzoic acid | M+H | 139.0394 | 9.169 |
| 23 | L-Lysine | M+H | 147.1139 | 4.933 |
| 24 | L-Methionine | M+H | 150.0597 | 3.187 |
| 25 | Guanine | M+H | 152.0566 | 3.968 |
| 26 | L-Histidine | M+H | 156.0777 | 4.889 |
| 27 | 3-Hydroxyoctanoic acid | M+H | 161.1295 | 4.792 |
| 28 | L-Carnitine | M+H | 162.1135 | 3.432 |
| 29 | L-Phenylalanine | M+H | 166.0849 | 3.099 |
| 30 | Uric Acid | M+H | 169.0365 | 3.161 |
| 31 | 1-Methylhistidine | M+H | 170.0933 | 4.840 |
| 32 | L-Arginine | M+H | 175.1198 | 4.889 |
| 33 | Citrulline | M+H | 176.1040 | 4.108 |
| 34 | Serotonin | M+H | 177.1035 | 1.631 |
| 35 | Myo-inositol | M+H | 181.0602 | 4.108 |
| 36 | D-Glucose | M+H | 181.0731 | 1.284 |
| 37 | L-Tyrosine | M+H | 182.0812 | 3.306 |
| 38 | L-Cl-Phe (IS) | M+H | 200.0489 | 2.600 |
| 39 | Asymmetric dimethylarginine | M+H | 203.1511 | 4.613 |
| 40 | L-Acetylcarnitine | M+H | 204.1245 | 2.542 |
| 41 | L-Tryptophan | M+H | 205.0867 | 9.319 |
| 42 | L-Cystine | M+H | 241.0321 | 4.915 |
| 43 | Glucose 6-phosphate | M+H | 261.0445 | 3.796 |
| 44 | 5'-Methylthioadenosine | M+H | 298.1020 | 4.235 |
| 45 | dUMP | M+H | 309.0482 | 3.271 |
| 46 | 3'-AMP | M+H | 348.0810 | 4.059 |
| 47 | dTDP | M+H | 403.0109 | 3.161 |
| 48 | PC (16:0/16:0) | M+H | 734.5718 | 1.959 |
| 49 | PE (22:6 (4Z,7Z,10Z,13Z,16Z,19Z)/P-16:0) | M+H | 748.5373 | 0.695 |
| 50 | Acetamide | M+H | 60.0446 | 0.657 |
| 51 | 2-Pyrrolidone | M+H | 86.0611 | 1.103 |
| 52 | 2-Piperidinone | M+H | 100.0767 | 1.156 |
| 53 | L-Glutamine | M+H | 147.0774 | 3.973 |
| 54 | Diethylphosphate | M+H | 155.0453 | 2.428 |
| 55 | O-Desmethylnaproxen | M+H | 217.0833 | 4.253 |
| 56 | Propionylcarnitine | M+H | 218.1402 | 2.472 |
| 57 | Carnosine | M+H | 227.1150 | 4.951 |
| 58 | Oleic acid | M+H | 283.2644 | 1.437 |
| 59 | Sphingosine | M+H | 300.2922 | 2.125 |
| 60 | Eicosadienoic acid | M+H | 309.2802 | 9.174 |
| 61 | Decanoylcarnitine | M+H | 316.2502 | 2.152 |
| 62 | Monooleoylglycerol | M+H | 357.3027 | 2.156 |
| 63 | 6-Keto-prostaglandin F1a | M+H | 371.2429 | 0.635 |
| 64 | Solanidine | M+H | 398.3410 | 2.117 |
| 65 | LysoPC (16:1 (9Z)/0:0) | M+H | 494.3268 | 2.375 |
| 66 | LysoPC (P-18:0) | M+H | 508.3766 | 2.249 |
| 67 | LysoPC (18:3 (6Z,9Z,12Z)) | M+H | 518.3232 | 3.310 |
| 68 | LysoPC (20:5 (5Z,8Z,11Z,14Z,17Z)) | M+H | 542.3270 | 2.324 |
| 69 | LysoPC (20:2 (11Z,14Z)) | M+H | 548.3714 | 2.302 |
| 70 | LysoPC (20:1 (11Z)) | M+H | 550.3881 | 2.288 |
| 71 | LysoPC (20:0/0:0) | M+H | 552.4044 | 2.280 |
| 72 | SM (d18:1/14:0) | M+H | 675.5457 | 2.205 |
| 73 | CE (20:3 (8Z,11Z,14Z)) | M+H | 675.6074 | 2.209 |
| 74 | SM (d18:0/16:1 (9Z)) | M+H | 703.5761 | 2.183 |
| 75 | PC (14:0/16:0) | M+H | 706.5433 | 1.968 |
| 76 | PC (14:0/18:2 (9Z,12Z)) | M+H | 730.5344 | 0.700 |
| 77 | PC (14:0/18:1 (11Z)) | M+H | 732.5549 | 1.704 |
| 78 | SM (d18:0/20:2 (11Z,14Z)) | M+H | 755.6079 | 2.147 |
| 79 | SM (d18:1/20:0) | M+H | 759.6334 | 2.152 |
| 80 | PC (14:0/20:1 (11Z)) | M+H | 760.5821 | 1.704 |
| 81 | PS (16:0/18:0) | M+H | 764.5370 | 0.617 |
| 82 | PC (18:3 (6Z,9Z,12Z)/P-18:1 (11Z)) | M+H | 766.5730 | 1.876 |
| 83 | PC (18:1 (9Z)/18:1 (9Z)) | M+H | 786.6012 | 1.907 |
| 84 | PS (18:0/18:0) | M+H | 792.5674 | 0.700 |
| 85 | PC (20:4 (5Z,8Z,11Z,14Z)/P-18:0) | M+H | 794.6036 | 0.748 |
| 86 | PC (20:3 (5Z,8Z,11Z)/P-18:1 (11Z)) | M+H | 794.6047 | 1.876 |
| 87 | PC (18:4 (6Z,9Z,12Z,15Z)/20:0) | M+H | 810.6001 | 1.880 |
| 88 | SM (d18:1/24:1 (15Z)) | M+H | 813.6836 | 2.125 |
| 89 | PC (18:1 (11Z)/22:5 (4Z,7Z,10Z,13Z,16Z)) | M+H | 834.6006 | 0.748 |
| 90 | PI (16:0/18:2 (9Z,12Z)) | M+H | 835.5308 | 2.893 |
| 91 | PI (16:0/20:4 (5Z,8Z,11Z,14Z)) | M+H | 859.5330 | 2.578 |
| 92 | PI (18:1 (11Z)/18:1 (11Z)) | M+H | 863.5642 | 2.582 |
| 93 | PI (20:4 (8Z,11Z,14Z,17Z)/18:0) | M+H | 887.5619 | 2.902 |
| 94 | PI (18:1 (11Z)/20:3 (5Z,8Z,11Z)) | M+H | 887.5638 | 2.573 |
