# Supplementary material for: Comprehensive metabolomic characterization of atrial fibrillation
Source: Front Cardiovasc Med. 2022 Aug 8;9:911845. doi: 10.3389/fcvm.2022.911845 (PMC9393302; doi:10.3389/fcvm.2022.911845)
Supplement: Supplementary file 8 [file Table_8.DOCX]

**Supplemental Table 8.** Statistical analysis of differential metabolites for each cross-comparisons in the discovery phase

| Differential metabolites | Mean1 | SE1 | Mean2 | SE2 | VIP | P | FDR | FC |
| --- | --- | --- | --- | --- | --- | --- | --- | --- |
| Comparison I: CP (n=143) vs. Control (n=86) | | | | | | | | |
| 2-Ketoglutaric Acid | 20947 | 832 | 11862 | 584 | 2.19 | ＜0.01 | ＜0.01 | 1.77 |
| 2-Hydroxy-3-methylbutyric acid | 15336 | 861 | 10292 | 571 | 1.47 | ＜0.01 | ＜0.01 | 1.49 |
| 2-Hydroxybutyric acid | 68994 | 3612 | 44946 | 1497 | 1.27 | ＜0.01 | ＜0.01 | 1.54 |
| 2-Pyrrolidone | 118751 | 4959 | 72715 | 4637 | 1.36 | ＜0.01 | ＜0.01 | 1.63 |
| 5-Hydroxytryptamine | 8861 | 356 | 12544 | 509 | 1.66 | ＜0.01 | ＜0.01 | 0.71 |
| 6-Keto-prostaglandin F1a | 81612 | 4365 | 111724 | 6273 | 1.50 | ＜0.01 | ＜0.01 | 0.73 |
| Alanine | 6277550 | 136857 | 5149829 | 210430 | 1.26 | ＜0.01 | ＜0.01 | 1.22 |
| Alpha-Tocopherol | 176880 | 4097 | 222379 | 7627 | 1.28 | ＜0.01 | ＜0.01 | 0.80 |
| Carnosine | 84879 | 7200 | 80283 | 14304 | 1.03 | 0.01 | 0.02 | 1.06 |
| CE (20:3 (8Z,11Z,14Z)) | 230998 | 10970 | 363891 | 21293 | 1.88 | ＜0.01 | ＜0.01 | 0.63 |
| Creatine | 1124692 | 71608 | 1429802 | 110829 | 1.03 | 0.01 | 0.02 | 0.79 |
| Diethylphosphate | 4938 | 295 | 3603 | 300 | 1.12 | ＜0.01 | ＜0.01 | 1.37 |
| Decanoylcarnitine | 7647463 | 246641 | 2601178 | 166348 | 3.09 | ＜0.01 | ＜0.01 | 2.94 |
| glycerol | 226468 | 5001 | 173971 | 4773 | 1.38 | ＜0.01 | ＜0.01 | 1.30 |
| Glycerol-2-phosphate | 44662 | 1571 | 66437 | 2712 | 1.94 | ＜0.01 | ＜0.01 | 0.67 |
| Glycerol-3-phosphate | 43421 | 1512 | 65642 | 2631 | 1.94 | ＜0.01 | ＜0.01 | 0.66 |
| Heptadecanoic acid | 14363 | 454 | 16348 | 463 | 1.00 | ＜0.01 | ＜0.01 | 0.88 |
| homocysteine | 4053 | 1014 | 676 | 61 | 1.71 | ＜0.01 | ＜0.01 | 6.00 |
| Isoleucine | 1361815 | 29689 | 1055790 | 29878 | 1.37 | ＜0.01 | ＜0.01 | 1.29 |
| Lactate | 4433192 | 86308 | 3118334 | 96023 | 1.93 | ＜0.01 | ＜0.01 | 1.42 |
| L-Arginine | 62162851 | 2152475 | 90069678 | 3954779 | 1.49 | ＜0.01 | ＜0.01 | 0.69 |
| L-Aspartic acid | 9833 | 281 | 7709 | 327 | 1.27 | ＜0.01 | ＜0.01 | 1.28 |
| lauric acid | 11827 | 411 | 15817 | 616 | 1.50 | ＜0.01 | ＜0.01 | 0.75 |
| Leucine | 2930900 | 59965 | 2405948 | 67504 | 1.12 | ＜0.01 | ＜0.01 | 1.22 |
| D-Glutamic acid | 853408 | 24851 | 600163 | 18867 | 1.65 | ＜0.01 | ＜0.01 | 1.42 |
| L-Serine | 13639 | 971 | 10313 | 1162 | 1.09 | 0.01 | 0.02 | 1.32 |
| LysoPC (20:0/0:0) | 1440490 | 72856 | 1843338 | 73065 | 1.09 | ＜0.01 | ＜0.01 | 0.78 |
| LysoPC (P-18:0) | 3124512 | 80280 | 4153637 | 123248 | 1.41 | ＜0.01 | ＜0.01 | 0.75 |
| Lyxose | 11575 | 452 | 34286 | 3647 | 2.59 | ＜0.01 | ＜0.01 | 0.34 |
| Niacinamide | 1032909 | 65466 | 1444240 | 121504 | 1.29 | ＜0.01 | ＜0.01 | 0.72 |
| Nicotinic acid | 2848 | 382 | 4155 | 604 | 1.67 | ＜0.01 | ＜0.01 | 0.69 |
| Oleic acid | 85254 | 4373 | 108220 | 4342 | 1.37 | ＜0.01 | ＜0.01 | 0.79 |
| O-PHOSPHORYLETHANOLAMINE | 13116 | 455 | 18905 | 774 | 1.77 | ＜0.01 | ＜0.01 | 0.69 |
| Ornithine | 1443660 | 34324 | 1184312 | 57622 | 1.32 | ＜0.01 | ＜0.01 | 1.22 |
| Oxalic acid | 1707364 | 52480 | 2249688 | 98346 | 1.16 | ＜0.01 | ＜0.01 | 0.76 |
| PC (14:0/18:2 (9Z,12Z)) | 845573 | 27662 | 1270283 | 79421 | 1.47 | ＜0.01 | ＜0.01 | 0.67 |
| PC (18:1 (9Z)/18:1 (9Z)) | 1189205646 | 30901531 | 1468007323 | 49770108 | 1.02 | ＜0.01 | ＜0.01 | 0.81 |
| PC (18:3 (6Z,9Z,12Z)/P-18:1 (11Z)) | 84503750 | 2395674 | 110005732 | 3840936 | 1.48 | ＜0.01 | ＜0.01 | 0.77 |
| PC (20:3 (5Z,8Z,11Z)/P-18:1 (11Z)) | 74677005 | 1911634 | 93050965 | 2939626 | 1.27 | ＜0.01 | ＜0.01 | 0.80 |
| PE (22:6 (4Z,7Z,10Z,13Z,16Z,19Z)/P-16:0) | 8822033 | 172339 | 10513499 | 238343 | 1.03 | ＜0.01 | ＜0.01 | 0.84 |
| Phenylalanine | 905472 | 18728 | 758047 | 18186 | 1.04 | ＜0.01 | ＜0.01 | 1.19 |
| Proline | 969389 | 51638 | 638123 | 83726 | 2.37 | ＜0.01 | ＜0.01 | 1.52 |
| SM (d18:0/16:1 (9Z)) | 911588296 | 26833526 | 1161876895 | 42397735 | 1.20 | ＜0.01 | ＜0.01 | 0.78 |
| SM (d18:1/14:0) | 39059758 | 1349235 | 55850403 | 2393231 | 1.74 | ＜0.01 | ＜0.01 | 0.70 |
| SM (d18:1/20:0) | 50090100 | 2016724 | 68534409 | 3098192 | 1.46 | ＜0.01 | ＜0.01 | 0.73 |
| Stearic acid | 613249 | 28511 | 727709 | 18625 | 1.20 | ＜0.01 | ＜0.01 | 0.84 |
| Taurine | 303159 | 10604 | 195506 | 8935 | 1.77 | ＜0.01 | ＜0.01 | 1.55 |
| Threonic acid | 85908 | 3527 | 115875 | 4379 | 1.55 | ＜0.01 | ＜0.01 | 0.74 |
| Tyrosine | 1465008 | 28464 | 1228253 | 30421 | 1.04 | ＜0.01 | ＜0.01 | 1.19 |
| γ-Aminobutyric acid | 62263 | 2175 | 49137 | 3819 | 1.25 | ＜0.01 | ＜0.01 | 1.27 |
| Comparison II: All-AFs plus Car-AF (n=113) vs. Sus- AF (n=30) | | | | | | | | |
| 2,3-Dihydroxybutanoic acid | 16167 | 1291 | 8604 | 733 | 1.31 | ＜0.01 | ＜0.01 | 1.88 |
| 2-Pyrrolidone | 135690 | 4950 | 54944 | 6303 | 2.40 | ＜0.01 | ＜0.01 | 2.47 |
| 3-Hydroxybutyric acid | 561497 | 116928 | 116521 | 15352 | 1.54 | ＜0.01 | ＜0.01 | 4.82 |
| 3-Hydroxyisobutyric acid | 58097 | 951 | 41364 | 2894 | 1.46 | ＜0.01 | 0.01 | 1.40 |
| 5'-Methylthioadenosine | 984441 | 45609 | 1339595 | 81122 | 1.04 | ＜0.01 | ＜0.01 | 0.73 |
| Agmatine | 11931 | 1300 | 4888 | 2286 | 2.23 | ＜0.01 | ＜0.01 | 2.44 |
| Betaine | 2742350 | 297059 | 5939769 | 444264 | 2.13 | ＜0.01 | ＜0.01 | 0.46 |
| CE (20:3 (8Z,11Z,14Z)) | 206552 | 10753 | 323079 | 27514 | 1.11 | ＜0.01 | ＜0.01 | 0.64 |
| Citrate | 521517 | 20979 | 357473 | 17298 | 1.20 | ＜0.01 | ＜0.01 | 1.46 |
| Creatine | 962219 | 66502 | 1736672 | 197493 | 1.52 | ＜0.01 | ＜0.01 | 0.55 |
| Creatinine | 3615156 | 174774 | 5172162 | 280029 | 1.07 | ＜0.01 | ＜0.01 | 0.70 |
| Cysteine | 77578 | 2060 | 123347 | 6030 | 1.41 | ＜0.01 | ＜0.01 | 0.63 |
| Cystine | 228569 | 9242 | 113661 | 8874 | 1.79 | ＜0.01 | ＜0.01 | 2.01 |
| D-Galactose | 16630 | 845 | 11647 | 1323 | 1.02 | ＜0.01 | ＜0.01 | 1.43 |
| Dihydrouracil | 189366 | 9409 | 273731 | 9137 | 1.28 | ＜0.01 | ＜0.01 | 0.69 |
| dTDP | 741722 | 56059 | 1329584 | 178950 | 1.30 | ＜0.01 | ＜0.01 | 0.56 |
| Epinephrine | 2712906 | 132386 | 3906403 | 169989 | 1.18 | ＜0.01 | ＜0.01 | 0.69 |
| Glutamine | 1759092 | 48739 | 1132343 | 44700 | 1.35 | ＜0.01 | ＜0.01 | 1.55 |
| Hippuric Acid | 10534 | 541 | 7040 | 562 | 1.37 | 0.01 | 0.01 | 1.50 |
| Histamine | 184522 | 12624 | 340217 | 38038 | 1.58 | ＜0.01 | ＜0.01 | 0.54 |
| homocysteine | 4916 | 1272 | 798 | 93 | 1.20 | ＜0.01 | ＜0.01 | 6.16 |
| Hypoxanthine | 3150712 | 194228 | 5890736 | 378981 | 1.83 | ＜0.01 | ＜0.01 | 0.53 |
| L-Arginine | 55876768 | 2125675 | 85840428 | 4235952 | 1.53 | ＜0.01 | ＜0.01 | 0.65 |
| L-Asparagine | 66767 | 2281 | 45332 | 3325 | 1.05 | ＜0.01 | ＜0.01 | 1.47 |
| L-Carnitine | 33279982 | 2480508 | 63213955 | 3426614 | 1.95 | ＜0.01 | ＜0.01 | 0.53 |
| L-Leucine | 32687 | 2669 | 24634 | 7074 | 1.05 | ＜0.01 | ＜0.01 | 1.33 |
| L-Lysine | 1406373 | 46959 | 1916096 | 61146 | 1.24 | ＜0.01 | ＜0.01 | 0.73 |
| L-Serine | 15881 | 1131 | 5194 | 527 | 1.84 | ＜0.01 | ＜0.01 | 3.06 |
| LysoPC (16:1 (9Z)/0:0) | 23896790 | 1263926 | 37186300 | 1545857 | 1.54 | ＜0.01 | ＜0.01 | 0.64 |
| LysoPC (20:0/0:0) | 1277796 | 49865 | 2053307 | 267078 | 1.30 | ＜0.01 | ＜0.01 | 0.62 |
| Malate | 13036 | 459 | 8859 | 342 | 1.23 | ＜0.01 | ＜0.01 | 1.47 |
| myo-Inositol | 110911 | 5692 | 189905 | 11883 | 1.55 | ＜0.01 | ＜0.01 | 0.58 |
| Niacinamide | 881856 | 55543 | 1601878 | 202551 | 1.39 | ＜0.01 | ＜0.01 | 0.55 |
| Oleic acid | 74483 | 4709 | 125825 | 7205 | 1.73 | ＜0.01 | ＜0.01 | 0.59 |
| PC (14:0/16:0) | 2107149 | 150653 | 3545766 | 420218 | 1.23 | ＜0.01 | ＜0.01 | 0.59 |
| PC (16:0/16:0) | 27434370 | 1076558 | 36609986 | 1049159 | 1.13 | ＜0.01 | ＜0.01 | 0.75 |
| PC (18:1 (9Z)/18:1 (9Z)) | 1104621966 | 33348959 | 1507804173 | 40983399 | 1.29 | ＜0.01 | ＜0.01 | 0.73 |
| PC (18:4 (6Z,9Z,12Z,15Z)/20:0) | 407924841 | 14097089 | 559383626 | 25425694 | 1.05 | ＜0.01 | ＜0.01 | 0.73 |
| PI (16:0/18:2 (9Z,12Z)) | 147991 | 5785 | 225139 | 15744 | 1.41 | ＜0.01 | ＜0.01 | 0.66 |
| PI (20:4 (8Z,11Z,14Z,17Z)/18:0) | 2628427 | 73073 | 3666724 | 154870 | 1.23 | ＜0.01 | ＜0.01 | 0.72 |
| Proline | 1091351 | 56693 | 509996 | 79186 | 1.93 | ＜0.01 | ＜0.01 | 2.14 |
| SM (d18:0/16:1 (9Z)) | 844928156 | 29646433 | 1162674824 | 35624793 | 1.25 | ＜0.01 | ＜0.01 | 0.73 |
| SM (d18:1/14:0) | 36360530 | 1470924 | 49226851 | 2550323 | 1.02 | ＜0.01 | ＜0.01 | 0.74 |
| SM (d18:1/20:0) | 44112644 | 2086733 | 72605181 | 3076190 | 1.61 | ＜0.01 | ＜0.01 | 0.61 |
| SM (d18:1/24:1 (15Z)) | 426777313 | 14439871 | 614781433 | 21689932 | 1.30 | ＜0.01 | ＜0.01 | 0.69 |
| S-Methyl-L-cysteine | 226885 | 22131 | 335507 | 31995 | 1.20 | ＜0.01 | ＜0.01 | 0.68 |
| Succinic acid | 36445 | 2161 | 61570 | 5034 | 1.52 | ＜0.01 | ＜0.01 | 0.59 |
| Taurine | 27614 | 3033 | 41929 | 3269 | 1.37 | ＜0.01 | ＜0.01 | 0.66 |
| Urea | 21031 | 1201 | 13443 | 1460 | 1.13 | ＜0.01 | ＜0.01 | 1.56 |
| Comparison III: All-AFs plus Car-AF (n=113) vs. Control (n=87) | | | | | | | | |
| 2,3-Dihydroxybutanoic acid | 16167 | 1291 | 10477 | 477 | 1.12 | ＜0.01 | ＜0.01 | 1.54 |
| 2-Hydroxy-3-methylbutyric acid | 15609 | 1026 | 10292 | 571 | 1.25 | ＜0.01 | ＜0.01 | 1.52 |
| 2-Hydroxybutyric acid | 73971 | 4373 | 44946 | 1497 | 1.57 | ＜0.01 | ＜0.01 | 1.65 |
| 2-Ketoglutaric Acid | 21106 | 958 | 11862 | 584 | 1.94 | ＜0.01 | ＜0.01 | 1.78 |
| 2-Pyrrolidone | 135690 | 4950 | 72715 | 4637 | 2.01 | ＜0.01 | ＜0.01 | 1.87 |
| 5-Hydroxytryptamine | 8740 | 395 | 12544 | 509 | 1.63 | ＜0.01 | ＜0.01 | 0.70 |
| 6-Keto-prostaglandin F1a | 80747 | 5097 | 111724 | 6273 | 1.28 | ＜0.01 | ＜0.01 | 0.72 |
| Alanine | 6481111 | 149511 | 5149829 | 210430 | 1.11 | ＜0.01 | ＜0.01 | 1.26 |
| Betaine | 2742350 | 297059 | 4490586 | 383615 | 1.19 | ＜0.01 | ＜0.01 | 0.61 |
| CE (20:3 (8Z,11Z,14Z)) | 206552 | 10753 | 363891 | 21293 | 1.79 | ＜0.01 | ＜0.01 | 0.57 |
| Creatine | 962219 | 66502 | 1429802 | 110829 | 1.28 | ＜0.01 | ＜0.01 | 0.67 |
| Cystine | 228569 | 9242 | 158188 | 6675 | 1.19 | ＜0.01 | ＜0.01 | 1.44 |
| Decanoylcarnitine | 8896029 | 82885 | 2601178 | 166348 | 2.83 | ＜0.01 | ＜0.01 | 3.42 |
| Glutamate | 881388 | 28352 | 600163 | 18867 | 1.57 | ＜0.01 | ＜0.01 | 1.47 |
| glycerol | 235555 | 5713 | 173971 | 4773 | 1.34 | ＜0.01 | ＜0.01 | 1.35 |
| Glycerol-2-phosphate | 46487 | 1867 | 66437 | 2712 | 1.50 | ＜0.01 | ＜0.01 | 0.70 |
| Glycerol-3-phosphate | 45210 | 1815 | 65642 | 2631 | 1.54 | ＜0.01 | ＜0.01 | 0.69 |
| homocysteine | 4916 | 1272 | 676 | 61 | 1.93 | ＜0.01 | ＜0.01 | 7.27 |
| Isoleucine | 1415739 | 33936 | 1055790 | 29878 | 1.33 | ＜0.01 | ＜0.01 | 1.34 |
| L-Acetylcarnitine | 38959634 | 1902429 | 24049157 | 1521654 | 1.52 | ＜0.01 | ＜0.01 | 1.62 |
| Lactose | 37593 | 2125 | 18375 | 1232 | 2.30 | ＜0.01 | ＜0.01 | 2.05 |
| L-Arginine | 55876768 | 2125675 | 90069678 | 3954779 | 1.70 | ＜0.01 | ＜0.01 | 0.62 |
| L-Asparagine | 66767 | 2281 | 51177 | 2557 | 1.12 | ＜0.01 | ＜0.01 | 1.30 |
| lauric acid | 11877 | 494 | 15817 | 616 | 1.19 | ＜0.01 | ＜0.01 | 0.75 |
| L-Carnitine | 33279982 | 2480508 | 50520116 | 2979143 | 1.36 | ＜0.01 | ＜0.01 | 0.66 |
| Leucine | 3043677 | 67302 | 2405948 | 67504 | 1.13 | ＜0.01 | ＜0.01 | 1.27 |
| L-Serine | 15881 | 1131 | 10313 | 1162 | 1.38 | ＜0.01 | ＜0.01 | 1.54 |
| LysoPC (20:0/0:0) | 1277796 | 49865 | 1843338 | 73065 | 1.62 | ＜0.01 | ＜0.01 | 0.69 |
| LysoPC (P-18:0) | 3056099 | 92629 | 4153637 | 123248 | 1.48 | ＜0.01 | ＜0.01 | 0.74 |
| Lyxose | 12182 | 527 | 34286 | 3647 | 2.15 | ＜0.01 | ＜0.01 | 0.36 |
| Malate | 13036 | 459 | 9685 | 315 | 1.18 | ＜0.01 | ＜0.01 | 1.35 |
| Niacinamide | 881856 | 55543 | 1444240 | 121504 | 1.37 | ＜0.01 | ＜0.01 | 0.61 |
| Nicotinic acid | 2771 | 421 | 4155 | 604 | 1.34 | ＜0.01 | ＜0.01 | 0.67 |
| Oleic acid | 74483 | 4709 | 108220 | 4342 | 1.60 | ＜0.01 | ＜0.01 | 0.69 |
| O-PHOSPHORYLETHANOLAMINE | 13916 | 531 | 18905 | 774 | 1.35 | ＜0.01 | ＜0.01 | 0.74 |
| Ornithine | 1517093 | 38052 | 1184312 | 57622 | 1.26 | ＜0.01 | ＜0.01 | 1.28 |
| PC (14:0/18:2 (9Z,12Z)) | 801936 | 30712 | 1270283 | 79421 | 1.52 | ＜0.01 | ＜0.01 | 0.63 |
| PC (18:1 (9Z)/18:1 (9Z)) | 1104621966 | 33348959 | 1468007323 | 49770108 | 1.29 | ＜0.01 | ＜0.01 | 0.75 |
| PC (18:3 (6Z,9Z,12Z)/P-18:1 (11Z)) | 80415870 | 2631566 | 110005732 | 3840936 | 1.39 | ＜0.01 | ＜0.01 | 0.73 |
| PC (20:3 (5Z,8Z,11Z)/P-18:1 (11Z)) | 71450278 | 2163184 | 93050965 | 2939626 | 1.22 | ＜0.01 | ＜0.01 | 0.77 |
| Phenylalanine | 940971 | 20647 | 758047 | 18186 | 1.11 | ＜0.01 | ＜0.01 | 1.24 |
| PI (20:4 (8Z,11Z,14Z,17Z)/18:0) | 2628427 | 73073 | 3371141 | 117359 | 1.06 | ＜0.01 | ＜0.01 | 0.78 |
| Proline | 1091351 | 56693 | 638123 | 83726 | 2.41 | ＜0.01 | ＜0.01 | 1.71 |
| SM (d18:0/16:1 (9Z)) | 844928156 | 29646433 | 1161876895 | 42397735 | 1.32 | ＜0.01 | ＜0.01 | 0.73 |
| SM (d18:1/14:0) | 36360530 | 1470924 | 55850403 | 2393231 | 1.59 | ＜0.01 | ＜0.01 | 0.65 |
| SM (d18:1/20:0) | 44112644 | 2086733 | 68534409 | 3098192 | 1.57 | ＜0.01 | ＜0.01 | 0.64 |
| SM (d18:1/24:1 (15Z)) | 426777313 | 14439871 | 558824606 | 21645057 | 1.02 | ＜0.01 | ＜0.01 | 0.76 |
| S-Methyl-L-cysteine | 226885 | 22131 | 291710 | 17802 | 1.04 | ＜0.01 | ＜0.01 | 0.78 |
| Stearic acid | 596359 | 35564 | 727709 | 18625 | 1.13 | ＜0.01 | ＜0.01 | 0.82 |
| Taurine | 319131 | 12514 | 195506 | 8935 | 1.76 | ＜0.01 | ＜0.01 | 1.63 |
| Threonic acid | 85582 | 4057 | 115875 | 4379 | 1.40 | ＜0.01 | ＜0.01 | 0.74 |
| γ-Aminobutyric acid | 62589 | 2135 | 49137 | 3819 | 1.19 | ＜0.01 | ＜0.01 | 1.27 |
| Comparison IV: Sus-AF (n=30) vs. Control (n=87) | | | | | | | | |
| 2-Hydroxy-3-methylbutyric acid | 14309 | 1385 | 10292 | 5295 | 1.29 | ＜0.01 | ＜0.01 | 1.39 |
| 2-Ketoglutaric Acid | 20347 | 1666 | 11862 | 5416 | 2.18 | ＜0.01 | ＜0.01 | 1.72 |
| 3-Hydroxybutyric acid | 116521 | 15352 | 200015 | 159809 | 1.58 | ＜0.01 | ＜0.01 | 0.58 |
| [5,8,11-Eicosatrienoic acid](https://hmdb.ca/metabolites/HMDB0010378) | 12715 | 620 | 16707 | 5152 | 1.30 | ＜0.01 | ＜0.01 | 0.76 |
| 5-Hydroxytryptamine | 9317 | 827 | 12544 | 4719 | 1.40 | ＜0.01 | 0.01 | 0.74 |
| Agmatine | 4888 | 2286 | 9727 | 14503 | 2.29 | ＜0.01 | ＜0.01 | 0.50 |
| Alpha-Tocopherol | 166483 | 10781 | 222379 | 70730 | 1.47 | ＜0.01 | ＜0.01 | 0.75 |
| Aminomalonic acid | 120937 | 7628 | 199269 | 87386 | 1.81 | ＜0.01 | ＜0.01 | 0.61 |
| Aminosuccinate | 11718 | 598 | 7709 | 3033 | 1.80 | ＜0.01 | ＜0.01 | 1.52 |
| Anthranilic acid | 50170 | 9305 | 80284 | 60628 | 1.44 | ＜0.01 | 0.01 | 0.62 |
| Asymmetric dimethylarginine | 2266918 | 130496 | 1784820 | 676170 | 1.08 | ＜0.01 | ＜0.01 | 1.27 |
| Betaine | 5939769 | 444264 | 4490586 | 3557498 | 1.62 | ＜0.01 | ＜0.01 | 1.32 |
| Cholesterol | 909433 | 55672 | 1213987 | 309470 | 1.44 | ＜0.01 | ＜0.01 | 0.75 |
| Citrate | 357473 | 17298 | 445763 | 117289 | 1.12 | ＜0.01 | ＜0.01 | 0.80 |
| Cysteine | 123347 | 6030 | 84739 | 23482 | 1.36 | ＜0.01 | ＜0.01 | 1.46 |
| Cystine | 113661 | 8874 | 158188 | 61900 | 1.17 | ＜0.01 | ＜0.01 | 0.72 |
| D-Galactose | 11647 | 1323 | 15970 | 9469 | 1.07 | 0.01 | 0.02 | 0.73 |
| Dihydrouracil | 273731 | 9137 | 224070 | 98794 | 1.18 | ＜0.01 | ＜0.01 | 1.22 |
| [D-Mannonic acid](https://hmdb.ca/metabolites/HMDB0242119) | 15444 | 506 | 20601 | 4631 | 1.50 | ＜0.01 | ＜0.01 | 0.75 |
| [Docosahexaenoic acid](https://hmdb.ca/metabolites/HMDB0002183) | 12715 | 620 | 16707 | 5152 | 1.30 | ＜0.01 | ＜0.01 | 0.76 |
| Glutamate | 748019 | 47287 | 600163 | 174965 | 1.13 | ＜0.01 | ＜0.01 | 1.25 |
| Glutamine | 1132343 | 44700 | 1823341 | 540200 | 1.84 | ＜0.01 | ＜0.01 | 0.62 |
| glyceric acid | 25690 | 1444 | 38162 | 12219 | 1.68 | ＜0.01 | ＜0.01 | 0.67 |
| Glycerol-2-phosphate | 37789 | 2205 | 66437 | 25147 | 2.38 | ＜0.01 | ＜0.01 | 0.57 |
| Glycerol-3-phosphate | 36685 | 1854 | 65642 | 24399 | 2.37 | ＜0.01 | ＜0.01 | 0.56 |
| Hippuric Acid | 7040 | 562 | 10414 | 4942 | 1.62 | 0.01 | 0.03 | 0.68 |
| Hypoxanthine | 5890736 | 378981 | 4112228 | 2432950 | 1.43 | ＜0.01 | ＜0.01 | 1.43 |
| Lactose | 32113 | 3654 | 18375 | 11424 | 1.81 | ＜0.01 | ＜0.01 | 1.75 |
| lauric acid | 11640 | 629 | 15817 | 5714 | 1.29 | ＜0.01 | ＜0.01 | 0.74 |
| L-Carnitine | 63213955 | 3426614 | 50520116 | 27627435 | 1.41 | ＜0.01 | ＜0.01 | 1.25 |
| Linoleic acid | 263440 | 16125 | 341701 | 101211 | 1.15 | ＜0.01 | ＜0.01 | 0.77 |
| L-Leucine | 24634 | 7074 | 33682 | 35067 | 1.12 | 0.01 | 0.04 | 0.73 |
| LysoPC (16:1 (9Z)/0:0) | 37186300 | 1545857 | 29794688 | 12352501 | 1.03 | ＜0.01 | ＜0.01 | 1.25 |
| Lyxose | 9289 | 709 | 34286 | 33822 | 2.68 | ＜0.01 | ＜0.01 | 0.27 |
| myo-Inositol | 189905 | 11883 | 131068 | 62538 | 1.43 | ＜0.01 | ＜0.01 | 1.45 |
| Myo-Inositol-2-phosphate | 45891 | 2583 | 35484 | 15161 | 1.02 | ＜0.01 | ＜0.01 | 1.29 |
| O-PHOSPHORYLETHANOLAMINE | 10101 | 570 | 18905 | 7174 | 2.50 | ＜0.01 | ＜0.01 | 0.53 |
| Oxalic acid | 1427304 | 85823 | 2249688 | 912019 | 1.60 | ＜0.01 | ＜0.01 | 0.63 |
| PC (14:0/18:1 (11Z)) | 20081694 | 1840227 | 13485924 | 6617430 | 1.32 | ＜0.01 | ＜0.01 | 1.49 |
| Phosphate | 309193 | 8799 | 381588 | 85724 | 1.11 | ＜0.01 | ＜0.01 | 0.81 |
| PI (16:0/18:2 (9Z,12Z)) | 225139 | 15744 | 153428 | 65778 | 1.25 | ＜0.01 | ＜0.01 | 1.47 |
| Pyruvate | 191314 | 12016 | 148366 | 68008 | 1.34 | ＜0.01 | 0.01 | 1.29 |
| Succinic acid | 61570 | 5034 | 42550 | 27339 | 1.29 | ＜0.01 | ＜0.01 | 1.45 |
| Taurine | 242998 | 13721 | 195506 | 82860 | 1.22 | ＜0.01 | ＜0.01 | 1.24 |
| Threonic acid | 87135 | 7134 | 115875 | 40611 | 1.33 | ＜0.01 | ＜0.01 | 0.75 |
| γ-Aminobutyric acid | 61036 | 6636 | 49137 | 35417 | 1.05 | 0.01 | 0.03 | 1.24 |
| Comparison V: All-AFs (n=81) vs. Control (n=87) | | | | | | | | |
| 2-Ketoglutaric Acid | 19119 | 1042 | 11862 | 584 | 1.67 | ＜0.01 | ＜0.01 | 1.61 |
| 2-Hydroxy-3-methylbutyric acid | 13572 | 685 | 10292 | 571 | 1.03 | ＜0.01 | ＜0.01 | 1.32 |
| 2-Hydroxybutyric acid | 58924 | 2517 | 44946 | 1497 | 1.11 | ＜0.01 | ＜0.01 | 1.31 |
| 2-Pyrrolidone | 136490 | 5928 | 72715 | 4637 | 2.20 | ＜0.01 | ＜0.01 | 1.88 |
| 4-Hydroxybenzoic acid | 374761 | 9174 | 306423 | 11527 | 1.11 | ＜0.01 | ＜0.01 | 1.22 |
| 5-Hydroxytryptamine | 9169 | 451 | 12544 | 509 | 1.37 | ＜0.01 | ＜0.01 | 0.73 |
| 6-Keto-prostaglandin F1a | 69753 | 5566 | 111724 | 6273 | 1.82 | ＜0.01 | ＜0.01 | 0.62 |
| Alanine | 6627791 | 164176 | 5149829 | 210430 | 1.33 | ＜0.01 | ＜0.01 | 1.29 |
| Betaine | 2587236 | 330760 | 4490586 | 383615 | 1.46 | ＜0.01 | 0.01 | 0.58 |
| CE (20:3 (8Z,11Z,14Z)) | 193065 | 12100 | 363891 | 21293 | 2.06 | ＜0.01 | ＜0.01 | 0.53 |
| Creatine | 951987 | 72352 | 1429802 | 110829 | 1.19 | ＜0.01 | ＜0.01 | 0.67 |
| Creatinine | 3382406 | 192474 | 4700097 | 231509 | 1.25 | ＜0.01 | ＜0.01 | 0.72 |
| Cystine | 207792 | 9060 | 158188 | 6675 | 1.08 | ＜0.01 | ＜0.01 | 1.31 |
| Decanoylcarnitine | 9698338 | 928841 | 2601178 | 166348 | 3.28 | ＜0.01 | ＜0.01 | 3.73 |
| Epinephrine | 2655189 | 153500 | 3656048 | 155219 | 1.11 | ＜0.01 | ＜0.01 | 0.73 |
| glycerol | 233694 | 5838 | 173971 | 4773 | 1.42 | ＜0.01 | ＜0.01 | 1.34 |
| Glycerol-2-phosphate | 50372 | 1970 | 66437 | 2712 | 1.18 | ＜0.01 | ＜0.01 | 0.76 |
| Glycerol-3-phosphate | 49058 | 1984 | 65642 | 2631 | 1.27 | ＜0.01 | ＜0.01 | 0.75 |
| Hypoxanthine | 2808918 | 183249 | 4112228 | 262352 | 1.15 | ＜0.01 | ＜0.01 | 0.68 |
| Isoleucine | 1411137 | 35723 | 1055790 | 29878 | 1.43 | ＜0.01 | ＜0.01 | 1.34 |
| L-Acetylcarnitine | 46184194 | 2654286 | 24049157 | 1521654 | 2.02 | ＜0.01 | ＜0.01 | 1.92 |
| Lactose | 30831 | 1575 | 18375 | 1232 | 2.00 | ＜0.01 | ＜0.01 | 1.68 |
| L-Arginine | 56370732 | 2649771 | 90069678 | 3954779 | 1.73 | ＜0.01 | ＜0.01 | 0.63 |
| L-Asparagine | 67661 | 2624 | 51177 | 2557 | 1.29 | ＜0.01 | ＜0.01 | 1.32 |
| lauric acid | 11813 | 588 | 15817 | 616 | 1.36 | ＜0.01 | ＜0.01 | 0.75 |
| L-Carnitine | 30993753 | 2890729 | 50520116 | 2979143 | 1.51 | ＜0.01 | ＜0.01 | 0.61 |
| Leucine | 3034913 | 68228 | 2405948 | 67504 | 1.23 | ＜0.01 | ＜0.01 | 1.26 |
| D-Glutamic acid | 882235 | 29697 | 600163 | 18867 | 1.71 | ＜0.01 | ＜0.01 | 1.47 |
| L-Histidine | 16263415 | 780766 | 22152786 | 878391 | 1.25 | ＜0.01 | ＜0.01 | 0.73 |
| L-Lysine | 1421534 | 59194 | 1808679 | 72552 | 1.00 | ＜0.01 | ＜0.01 | 0.79 |
| L-Serine | 16576 | 1275 | 10313 | 1162 | 1.63 | ＜0.01 | ＜0.01 | 1.61 |
| LysoPC (20:0/0:0) | 1403448 | 57444 | 1843338 | 73065 | 1.21 | ＜0.01 | ＜0.01 | 0.76 |
| LysoPC (P-18:0) | 3236850 | 107913 | 4153637 | 123248 | 1.25 | ＜0.01 | ＜0.01 | 0.78 |
| Lyxose | 11520 | 562 | 34286 | 3647 | 2.38 | ＜0.01 | ＜0.01 | 0.34 |
| Methionine | 236913 | 4759 | 197116 | 5647 | 1.05 | ＜0.01 | ＜0.01 | 1.20 |
| Nicotinic acid | 2979 | 536 | 4155 | 604 | 1.31 | 0.01 | 0.03 | 0.72 |
| Oleic acid | 65121 | 4639 | 108220 | 4342 | 2.18 | ＜0.01 | ＜0.01 | 0.60 |
| O-PHOSPHORYLETHANOLAMINE | 15090 | 596 | 18905 | 774 | 1.04 | ＜0.01 | ＜0.01 | 0.80 |
| Ornithine | 1534740 | 46212 | 1184312 | 57622 | 1.38 | ＜0.01 | ＜0.01 | 1.30 |
| PC (14:0/18:2 (9Z,12Z)) | 823561 | 38813 | 1270283 | 79421 | 1.53 | ＜0.01 | ＜0.01 | 0.65 |
| PC (16:0/16:0) | 25369275 | 1209221 | 32430680 | 1248491 | 1.00 | ＜0.01 | ＜0.01 | 0.78 |
| PC (18:1 (9Z)/18:1 (9Z)) | 1124741422 | 39461071 | 1468007323 | 49770108 | 1.23 | ＜0.01 | ＜0.01 | 0.77 |
| PC (18:3 (6Z,9Z,12Z)/P-18:1 (11Z)) | 80562455 | 3349553 | 110005732 | 3840936 | 1.42 | ＜0.01 | ＜0.01 | 0.73 |
| PC (18:4 (6Z,9Z,12Z,15Z)/20:0) | 390136087 | 17259424 | 492965270 | 19557225 | 1.04 | ＜0.01 | ＜0.01 | 0.79 |
| PC (20:3 (5Z,8Z,11Z)/P-18:1 (11Z)) | 70311690 | 2673426 | 93050965 | 2939626 | 1.32 | ＜0.01 | ＜0.01 | 0.76 |
| PI (20:4 (8Z,11Z,14Z,17Z)/18:0) | 2627328 | 86019 | 3371141 | 117359 | 1.08 | ＜0.01 | ＜0.01 | 0.78 |
| Proline | 1119118 | 70817 | 638123 | 83726 | 2.60 | ＜0.01 | ＜0.01 | 1.75 |
| SM (d18:0/16:1 (9Z)) | 819289157 | 34424013 | 1161876895 | 42397735 | 1.51 | ＜0.01 | ＜0.01 | 0.71 |
| SM (d18:0/20:2 (11Z,14Z)) | 799875 | 28461 | 1020364 | 37330 | 1.05 | ＜0.01 | ＜0.01 | 0.78 |
| SM (d18:1/14:0) | 34451478 | 1611212 | 55850403 | 2393231 | 1.83 | ＜0.01 | ＜0.01 | 0.62 |
| SM (d18:1/20:0) | 42939963 | 2420069 | 68534409 | 3098192 | 1.73 | ＜0.01 | ＜0.01 | 0.63 |
| SM (d18:1/24:1 (15Z)) | 422651793 | 17553552 | 558824606 | 21645057 | 1.11 | ＜0.01 | ＜0.01 | 0.76 |
| S-Methyl-L-cysteine | 233313 | 28711 | 291710 | 17802 | 1.15 | ＜0.01 | ＜0.01 | 0.80 |
| Stearic acid | 602130 | 39959 | 727709 | 18625 | 1.26 | ＜0.01 | ＜0.01 | 0.83 |
| Taurine | 298606 | 12964 | 195506 | 8935 | 1.79 | ＜0.01 | ＜0.01 | 1.53 |
| Threonic acid | 88972 | 4716 | 115875 | 4379 | 1.15 | ＜0.01 | ＜0.01 | 0.77 |
| Tyrosine | 1496469 | 33338 | 1228253 | 30421 | 1.09 | ＜0.01 | ＜0.01 | 1.22 |
| Urea | 22290 | 1276 | 17337 | 1466 | 1.43 | ＜0.01 | ＜0.01 | 1.29 |
| Uric Acid | 1296068 | 104698 | 966334 | 175403 | 1.02 | 0.02 | 0.04 | 1.34 |
| Valine | 789541 | 16474 | 648037 | 16892 | 1.13 | ＜0.01 | ＜0.01 | 1.22 |
| γ-Aminobutyric acid | 59730 | 2156 | 49137 | 3819 | 1.02 | ＜0.01 | ＜0.01 | 1.22 |
| Comparison VI: All-AFs (n=81) vs. Car-AF (n=32) | | | | | | | | |
| 11Z-Eicosenoic acid | 8620 | 479 | 11977 | 1241 | 1.07 | ＜0.01 | 0.01 | 1.39 |
| 2,3-Dihydroxybutanoic acid | 11711 | 599 | 27445 | 3633 | 2.01 | ＜0.01 | ＜0.01 | 2.34 |
| 2-Deoxygalactose | 1281486 | 87326 | 845981 | 89688 | 1.05 | 0.02 | 0.05 | 0.66 |
| 2-Hydroxybutyric acid | 58924 | 2517 | 112059 | 11718 | 1.94 | ＜0.01 | ＜0.01 | 1.90 |
| 2-Ketoglutaric Acid | 19119 | 1042 | 26136 | 1863 | 1.11 | ＜0.01 | 0.01 | 1.37 |
| 3-Hydroxybutyric acid | 223294 | 50765 | 1417573 | 353008 | 3.42 | ＜0.01 | ＜0.01 | 6.35 |
| 6-Keto-prostaglandin F1a | 69753 | 5566 | 108573 | 9707 | 1.47 | ＜0.01 | ＜0.01 | 1.56 |
| 9-Hexadecenoic acid | 40794 | 2744 | 74516 | 6642 | 1.87 | ＜0.01 | ＜0.01 | 1.83 |
| Aminomalonic acid | 186493 | 8164 | 132310 | 14868 | 1.46 | ＜0.01 | ＜0.01 | 0.71 |
| Asymmetric dimethylarginine | 1590550 | 82093 | 2196622 | 183032 | 1.20 | ＜0.01 | ＜0.01 | 1.38 |
| Citrate | 458137 | 17488 | 681946 | 49588 | 1.44 | ＜0.01 | ＜0.01 | 1.49 |
| D-Malic acid | 11832 | 463 | 16085 | 933 | 1.12 | ＜0.01 | ＜0.01 | 1.36 |
| dUMP | 133967 | 8938 | 67903 | 6429 | 1.53 | ＜0.01 | ＜0.01 | 0.51 |
| Glucitol | 21950 | 3820 | 914725 | 557929 | 3.25 | ＜0.01 | ＜0.01 | 41.67 |
| Glucose 6-phosphate | 165997 | 7453 | 111160 | 10224 | 1.44 | ＜0.01 | ＜0.01 | 0.67 |
| Glycerol-2-phosphate | 50372 | 1970 | 36651 | 3840 | 1.27 | ＜0.01 | ＜0.01 | 0.73 |
| Glycerol-3-phosphate | 49058 | 1984 | 35470 | 3469 | 1.28 | ＜0.01 | ＜0.01 | 0.72 |
| homocysteine | 971 | 91 | 14904 | 4011 | 3.86 | ＜0.01 | ＜0.01 | 15.36 |
| Lactose | 30831 | 1575 | 54709 | 5311 | 1.46 | ＜0.01 | ＜0.01 | 1.77 |
| L-Cysteine | 330531 | 14634 | 439056 | 41697 | 1.01 | 0.00 | 0.01 | 1.33 |
| Linoleic acid | 315921 | 13436 | 414202 | 23513 | 1.20 | ＜0.01 | ＜0.01 | 1.31 |
| LysoPC (18:3 (6Z,9Z,12Z)) | 120812 | 4602 | 98363 | 10236 | 1.06 | 0.01 | 0.04 | 0.81 |
| LysoPC (20:0/0:0) | 1403448 | 57444 | 959739 | 74933 | 1.41 | ＜0.01 | ＜0.01 | 0.68 |
| meso-Erythritol | 40655 | 1197 | 56892 | 3812 | 1.15 | ＜0.01 | ＜0.01 | 1.40 |
| Methyl galactoside | 87744 | 3925 | 130888 | 10956 | 1.37 | ＜0.01 | ＜0.01 | 1.49 |
| Myo-Inositol | 180835 | 5214 | 258305 | 14424 | 1.47 | ＜0.01 | ＜0.01 | 1.43 |
| Myo-Inositol-2-phosphate | 41892 | 3120 | 56012 | 3930 | 1.08 | ＜0.01 | 0.01 | 1.34 |
| Niacinamide | 978447 | 66963 | 637360 | 85897 | 1.65 | ＜0.01 | ＜0.01 | 0.65 |
| Oleic acid | 65121 | 4639 | 98182 | 10818 | 1.39 | ＜0.01 | 0.01 | 1.51 |
| O-PHOSPHORYLETHANOLAMINE | 15090 | 596 | 10944 | 938 | 1.20 | ＜0.01 | ＜0.01 | 0.73 |
| Oxalic acid | 1914160 | 69878 | 1446467 | 99804 | 1.06 | ＜0.01 | ＜0.01 | 0.76 |
| PC (16:0/16:0) | 25369275 | 1209221 | 32661644 | 2001274 | 1.01 | ＜0.01 | 0.01 | 1.29 |
| Pyruvate | 163432 | 9374 | 249499 | 25587 | 1.38 | ＜0.01 | 0.01 | 1.53 |
| Ribitol | 512500 | 27755 | 933829 | 81470 | 1.93 | ＜0.01 | ＜0.01 | 1.82 |
| SM (d18:0/20:2 (11Z,14Z)) | 799875 | 28461 | 1159992 | 85912 | 1.32 | ＜0.01 | ＜0.01 | 1.45 |
| Uridine | 21383 | 695 | 16176 | 931 | 1.15 | ＜0.01 | ＜0.01 | 0.76 |
| Comparison VII: Car-AF (n=32) vs. Control (n=87) | | | | | | | | |
| 11Z-Eicosenoic acid | 11977 | 1241 | 7646 | 395 | 1.20 | ＜0.01 | ＜0.01 | 1.57 |
| 2,3-Dihydroxybutanoic acid | 27445 | 3633 | 10477 | 477 | 1.85 | ＜0.01 | ＜0.01 | 2.62 |
| 2-Hydroxy-3-methylbutyric acid | 20765 | 3030 | 10292 | 571 | 1.32 | ＜0.01 | ＜0.01 | 2.02 |
| 2-Hydroxybutyric acid | 112059 | 11718 | 44946 | 1497 | 2.04 | ＜0.01 | ＜0.01 | 2.49 |
| 2-Ketoglutaric Acid | 26136 | 1863 | 11862 | 584 | 1.95 | ＜0.01 | ＜0.01 | 2.20 |
| 2-Pyrrolidone | 133667 | 9106 | 72715 | 4637 | 1.52 | ＜0.01 | ＜0.01 | 1.84 |
| 3-Hydroxybutyric acid | 1417573 | 353008 | 200015 | 17233 | 2.50 | ＜0.01 | ＜0.01 | 7.09 |
| 5-Hydroxytryptamine | 7654 | 780 | 12544 | 509 | 1.50 | ＜0.01 | ＜0.01 | 0.61 |
| 9-Hexadecenoic acid | 74516 | 6642 | 36577 | 1969 | 1.71 | ＜0.01 | ＜0.01 | 2.04 |
| Aminomalonic acid | 132310 | 14868 | 199269 | 9423 | 1.20 | ＜0.01 | ＜0.01 | 0.66 |
| Citrate | 681946 | 49588 | 445763 | 12648 | 1.19 | ＜0.01 | ＜0.01 | 1.53 |
| Creatine | 988117 | 148989 | 1429802 | 110829 | 1.09 | ＜0.01 | 0.01 | 0.69 |
| Cystine | 281163 | 20716 | 158188 | 6675 | 1.32 | ＜0.01 | ＜0.01 | 1.78 |
| D-Malic acid | 16085 | 933 | 9685 | 315 | 1.53 | ＜0.01 | ＜0.01 | 1.66 |
| Glucitol | 914725 | 557929 | 601790 | 229623 | 1.68 | ＜0.01 | 0.01 | 1.52 |
| Glucose 6-phosphate | 111160 | 10224 | 173411 | 7436 | 1.37 | ＜0.01 | ＜0.01 | 0.64 |
| Glycerol-2-phosphate | 36651 | 3840 | 66437 | 2712 | 1.62 | ＜0.01 | ＜0.01 | 0.55 |
| Glycerol-3-phosphate | 35470 | 3469 | 65642 | 2631 | 1.65 | ＜0.01 | ＜0.01 | 0.54 |
| homocysteine | 14904 | 4011 | 676 | 61 | 3.38 | ＜0.01 | ＜0.01 | 22.05 |
| Lactose | 54709 | 5311 | 18375 | 1232 | 2.29 | ＜0.01 | ＜0.01 | 2.98 |
| L-Arginine | 54626422 | 3422284 | 90069678 | 3954779 | 1.17 | ＜0.01 | ＜0.01 | 0.61 |
| L-Aspartic acid | 10791 | 620 | 7709 | 327 | 1.03 | ＜0.01 | ＜0.01 | 1.40 |
| L-Glutamic acid | 879243 | 67038 | 600163 | 18867 | 1.10 | ＜0.01 | ＜0.01 | 1.47 |
| LysoPC (20:0/0:0) | 959739 | 74933 | 1843338 | 73065 | 1.76 | ＜0.01 | ＜0.01 | 0.52 |
| LysoPC (P-18:0) | 2598574 | 154841 | 4153637 | 123248 | 1.38 | ＜0.01 | ＜0.01 | 0.63 |
| Lyxose | 13856 | 1164 | 34286 | 3647 | 1.30 | ＜0.01 | ＜0.01 | 0.40 |
| Methyl galactoside | 130888 | 10956 | 88672 | 3566 | 1.11 | ＜0.01 | ＜0.01 | 1.48 |
| Myo-Inositol | 258305 | 14424 | 183953 | 4379 | 1.13 | ＜0.01 | ＜0.01 | 1.40 |
| Myo-Inositol-2-phosphate | 56012 | 3930 | 35484 | 1635 | 1.14 | ＜0.01 | ＜0.01 | 1.58 |
| Niacinamide | 637360 | 85897 | 1444240 | 121504 | 1.82 | ＜0.01 | ＜0.01 | 0.44 |
| Nicotinic acid | 2245 | 611 | 4155 | 604 | 1.62 | ＜0.01 | ＜0.01 | 0.54 |
| Oleic acid | 927344 | 56452 | 611140 | 22199 | 1.35 | ＜0.01 | ＜0.01 | 1.52 |
| O-PHOSPHORYLETHANOLAMINE | 10944 | 938 | 18905 | 774 | 1.49 | ＜0.01 | ＜0.01 | 0.58 |
| Oxalic acid | 1446467 | 99804 | 2249688 | 98346 | 1.04 | ＜0.01 | ＜0.01 | 0.64 |
| PC (14:0/18:2 (9Z,12Z)) | 747195 | 45373 | 1270283 | 79421 | 1.14 | ＜0.01 | ＜0.01 | 0.59 |
| Phenylalanine | 1036310 | 49031 | 758047 | 18186 | 1.06 | ＜0.01 | ＜0.01 | 1.37 |
| Proline | 1021066 | 89576 | 638123 | 83726 | 1.49 | ＜0.01 | ＜0.01 | 1.60 |
| Pyruvate | 249499 | 25587 | 148366 | 7334 | 1.20 | ＜0.01 | ＜0.01 | 1.68 |
| Ribitol | 933829 | 81470 | 481724 | 11680 | 1.71 | ＜0.01 | ＜0.01 | 1.94 |
| Taurine | 371083 | 27906 | 195506 | 8935 | 1.46 | ＜0.01 | ＜0.01 | 1.90 |
| Threonic acid | 77003 | 7834 | 115875 | 4379 | 1.30 | ＜0.01 | ＜0.01 | 0.66 |
| Uridine | 16176 | 931 | 22654 | 630 | 1.08 | ＜0.01 | ＜0.01 | 0.71 |
| γ-Aminobutyric acid | 69825 | 5044 | 49137 | 3819 | 1.12 | ＜0.01 | ＜0.01 | 1.42 |
| Comparison VIII: Fir-AF (n=22) vs. Control (n=87) | | | | | | | | |
| 2-Ketoglutaric Acid | 19473 | 2235 | 11862 | 584 | 1.40 | ＜0.01 | 0.01 | 1.64 |
| 2-Hydroxy-3-methylbutyric acid | 14723 | 1116 | 10292 | 571 | 1.50 | ＜0.01 | ＜0.01 | 1.43 |
| 2-Pyrrolidone | 161314 | 13209 | 72715 | 4637 | 2.55 | ＜0.01 | ＜0.01 | 2.22 |
| 4-Hydroxybenzoic acid | 389694 | 11253 | 306423 | 11527 | 1.32 | ＜0.01 | ＜0.01 | 1.27 |
| 6-Keto-prostaglandin F1a | 72841 | 8676 | 111724 | 6273 | 1.04 | ＜0.01 | ＜0.01 | 0.65 |
| Alanine | 6620907 | 257314 | 5149829 | 210430 | 1.27 | ＜0.01 | ＜0.01 | 1.29 |
| Asparagine | 94349 | 2719 | 78415 | 2587 | 1.17 | ＜0.01 | ＜0.01 | 1.20 |
| Betaine | 1883962 | 225717 | 4490586 | 383615 | 1.48 | ＜0.01 | 0.01 | 0.42 |
| Buprenorphine | 12144338 | 1200898 | 17581994 | 1013591 | 1.08 | 0.01 | 0.02 | 0.69 |
| CE (20:3 (8Z,11Z,14Z)) | 188427 | 16988 | 363891 | 21293 | 1.97 | ＜0.01 | ＜0.01 | 0.52 |
| Creatinine | 3154684 | 215424 | 4700097 | 231509 | 1.14 | ＜0.01 | ＜0.01 | 0.67 |
| Cystine | 205457 | 15153 | 158188 | 6675 | 1.11 | ＜0.01 | 0.01 | 1.30 |
| Decanoylcarnitine | 9286206 | 758717 | 2601178 | 166348 | 2.71 | ＜0.01 | ＜0.01 | 3.57 |
| Diethylphosphate | 5720 | 776 | 3603 | 300 | 1.43 | 0.02 | 0.06 | 1.59 |
| Glutamate-3TMS | 899294 | 51018 | 600163 | 18867 | 1.79 | ＜0.01 | ＜0.01 | 1.50 |
| Glycerol-2-phosphate | 48981 | 3542 | 66437 | 2712 | 1.11 | ＜0.01 | 0.01 | 0.74 |
| Glycerol-3-phosphate | 47986 | 3585 | 65642 | 2631 | 1.15 | ＜0.01 | ＜0.01 | 0.73 |
| glycerol-3TMS | 243540 | 11754 | 173971 | 4773 | 1.41 | ＜0.01 | ＜0.01 | 1.40 |
| Heptadecanoic acid | 13619 | 1114 | 16348 | 463 | 1.19 | ＜0.01 | 0.01 | 0.83 |
| Histidine | 621027 | 37470 | 508095 | 27616 | 1.17 | ＜0.01 | ＜0.01 | 1.22 |
| Hypoxanthine | 2551508 | 303285 | 4112228 | 262352 | 1.55 | ＜0.01 | 0.01 | 0.62 |
| Isoleucine | 1460203 | 71228 | 1055790 | 29878 | 1.43 | ＜0.01 | ＜0.01 | 1.38 |
| L-Acetylcarnitine | 51224704 | 2326069 | 24049157 | 1521654 | 1.97 | ＜0.01 | ＜0.01 | 2.13 |
| Lactose | 30573 | 2452 | 18375 | 1232 | 2.07 | ＜0.01 | ＜0.01 | 1.66 |
| L-Arginine | 56153820 | 3003144 | 90069678 | 3954779 | 1.12 | ＜0.01 | ＜0.01 | 0.62 |
| L-Asparagine | 63762 | 3272 | 51177 | 2557 | 1.06 | ＜0.01 | ＜0.01 | 1.25 |
| lauric acid | 11009 | 992 | 15817 | 616 | 1.85 | ＜0.01 | ＜0.01 | 0.70 |
| L-Carnitine | 28567244 | 4520236 | 50520116 | 2979143 | 1.15 | 0.02 | 0.05 | 0.57 |
| Leucine | 3139096 | 118900 | 2405948 | 67504 | 1.19 | ＜0.01 | ＜0.01 | 1.30 |
| L-Serine | 16112 | 2192 | 10313 | 1162 | 1.84 | ＜0.01 | ＜0.01 | 1.56 |
| Lysine | 740601 | 24712 | 608734 | 14359 | 1.18 | ＜0.01 | ＜0.01 | 1.22 |
| LysoPC (16:1 (9Z)/0:0) | 21808609 | 1601055 | 29794688 | 1332004 | 1.09 | 0.01 | 0.02 | 0.73 |
| LysoPC (20:2 (11Z,14Z)) | 8167585 | 665378 | 6246452 | 228929 | 1.27 | ＜0.01 | ＜0.01 | 1.31 |
| Lyxose | 13428 | 1272 | 34286 | 3647 | 1.68 | ＜0.01 | ＜0.01 | 0.39 |
| Methionine | 242693 | 8427 | 197116 | 5647 | 1.10 | ＜0.01 | ＜0.01 | 1.23 |
| Monooleoylglycerol | 1508174 | 72423 | 2308194 | 97294 | 1.44 | ＜0.01 | ＜0.01 | 0.65 |
| Niacinamide | 885544 | 150083 | 1444240 | 121504 | 1.27 | 0.01 | 0.03 | 0.61 |
| O-Desmethylnaproxen | 392071 | 43952 | 280679 | 13268 | 1.22 | ＜0.01 | 0.01 | 1.40 |
| Oleic acid | 58973 | 7228 | 108220 | 4342 | 2.13 | ＜0.01 | ＜0.01 | 0.54 |
| Ornithine | 1496140 | 76355 | 1184312 | 57622 | 1.33 | ＜0.01 | ＜0.01 | 1.26 |
| PC (14:0/18:2 (9Z,12Z)) | 809993 | 40930 | 1270283 | 79421 | 1.02 | ＜0.01 | ＜0.01 | 0.64 |
| PC (16:0/16:0) | 23388309 | 1774908 | 32430680 | 1248491 | 1.24 | ＜0.01 | ＜0.01 | 0.72 |
| PC (18:3 (6Z,9Z,12Z)/P-18:1 (11Z)) | 79221983 | 5137792 | 110005732 | 3840936 | 1.24 | ＜0.01 | ＜0.01 | 0.72 |
| PC (18:4 (6Z,9Z,12Z,15Z)/20:0) | 355056821 | 23122771 | 492965270 | 19557225 | 1.15 | ＜0.01 | ＜0.01 | 0.72 |
| PC (20:3 (5Z,8Z,11Z)/P-18:1 (11Z)) | 66490142 | 3876535 | 93050965 | 2939626 | 1.20 | ＜0.01 | ＜0.01 | 0.71 |
| Phenylalanine | 946122 | 35782 | 758047 | 18186 | 1.18 | ＜0.01 | ＜0.01 | 1.25 |
| PI (20:4 (8Z,11Z,14Z,17Z)/18:0) | 2515971 | 118766 | 3371141 | 117359 | 1.22 | ＜0.01 | ＜0.01 | 0.75 |
| Proline | 1009340 | 55085 | 638123 | 83726 | 2.49 | ＜0.01 | ＜0.01 | 1.58 |
| Pyroglutamate-TMS | 5395113 | 289074 | 4647478 | 159921 | 1.09 | 0.01 | 0.02 | 1.16 |
| SM (d18:0/16:1 (9Z)) | 816978975 | 47459279 | 1161876895 | 42397735 | 1.20 | ＜0.01 | ＜0.01 | 0.70 |
| SM (d18:0/20:2 (11Z,14Z)) | 787938 | 44666 | 1020364 | 37330 | 1.11 | ＜0.01 | 0.01 | 0.77 |
| SM (d18:1/14:0) | 32406916 | 2421316 | 55850403 | 2393231 | 1.91 | ＜0.01 | ＜0.01 | 0.58 |
| SM (d18:1/20:0) | 37244724 | 1977999 | 68534409 | 3098192 | 1.87 | ＜0.01 | ＜0.01 | 0.54 |
| SM (d18:1/24:1 (15Z)) | 397426374 | 21735867 | 558824606 | 21645057 | 1.12 | ＜0.01 | ＜0.01 | 0.71 |
| S-Methyl-L-cysteine | 184813 | 40291 | 291710 | 17802 | 1.84 | ＜0.01 | ＜0.01 | 0.63 |
| Stearic acid | 581843 | 70084 | 727709 | 18625 | 1.52 | 0.01 | 0.03 | 0.80 |
| Taurine | 292006 | 23867 | 195506 | 8935 | 1.62 | ＜0.01 | ＜0.01 | 1.49 |
| Tryptophan | 1256689 | 48513 | 1043613 | 32429 | 1.11 | ＜0.01 | ＜0.01 | 1.20 |
| Tyrosine | 1509851 | 54121 | 1228253 | 30421 | 1.08 | ＜0.01 | ＜0.01 | 1.23 |
| Urea-3TMS | 22637 | 2456 | 17337 | 1466 | 1.44 | ＜0.01 | 0.01 | 1.31 |
| Uric acid | 1265898 | 150277 | 966334 | 175403 | 1.05 | 0.01 | 0.03 | 1.31 |
| Valine | 805063 | 31312 | 648037 | 16892 | 1.02 | ＜0.01 | ＜0.01 | 1.24 |
| γ-Aminobutyric acid | 64463 | 5437 | 49137 | 3819 | 1.23 | ＜0.01 | ＜0.01 | 1.31 |
| Comparison IX: Par-AF (n=33) vs. Control (n=87) | | | | | | | | |
| 2-Ketoglutaric Acid | 17254 | 1646 | 11862 | 584 | 1.61 | ＜0.01 | 0.01 | 1.45 |
| 2-Pyrrolidone | 122485 | 7437 | 72715 | 4637 | 2.03 | ＜0.01 | ＜0.01 | 1.68 |
| 5-Hydroxytryptamine | 9845 | 702 | 12544 | 509 | 1.03 | 0.01 | 0.02 | 0.78 |
| 6-Keto-prostaglandin F1a | 68419 | 8315 | 111724 | 6273 | 2.14 | ＜0.01 | ＜0.01 | 0.61 |
| Alanine | 6119174 | 254617 | 5149829 | 210430 | 1.11 | ＜0.01 | 0.01 | 1.19 |
| Alpha-Tocopherol | 168150 | 5333 | 222379 | 7627 | 1.46 | ＜0.01 | ＜0.01 | 0.76 |
| Buprenorphine | 12650580 | 1082222 | 17581994 | 1013591 | 1.24 | 0.01 | 0.02 | 0.72 |
| CE (20:3 (8Z,11Z,14Z)) | 205801 | 25395 | 363891 | 21293 | 2.25 | ＜0.01 | ＜0.01 | 0.57 |
| Cystine | 199850 | 11961 | 158188 | 6675 | 1.00 | ＜0.01 | 0.01 | 1.26 |
| Decanoylcarnitine | 8974064 | 318036 | 2601178 | 166348 | 3.75 | ＜0.01 | ＜0.01 | 3.45 |
| Glutamate-3TMS | 780240 | 39302 | 600163 | 18867 | 1.52 | ＜0.01 | ＜0.01 | 1.30 |
| Glycerol-2-phosphate | 47939 | 3188 | 66437 | 2712 | 1.58 | ＜0.01 | ＜0.01 | 0.72 |
| Glycerol-3-phosphate | 46449 | 3312 | 65642 | 2631 | 1.70 | ＜0.01 | ＜0.01 | 0.71 |
| glycerol-3TMS | 213525 | 7109 | 173971 | 4773 | 1.24 | ＜0.01 | ＜0.01 | 1.23 |
| Isoleucine | 1293984 | 42534 | 1055790 | 29878 | 1.29 | ＜0.01 | ＜0.01 | 1.23 |
| L-Acetylcarnitine | 41364550 | 2644644 | 24049157 | 1521654 | 1.90 | ＜0.01 | ＜0.01 | 1.72 |
| Lactose | 26449 | 1881 | 18375 | 1232 | 1.91 | ＜0.01 | ＜0.01 | 1.44 |
| L-Arginine | 63091801 | 5144395 | 90069678 | 3954779 | 1.41 | ＜0.01 | ＜0.01 | 0.70 |
| L-Asparagine | 72386 | 5309 | 51177 | 2557 | 1.28 | ＜0.01 | 0.01 | 1.41 |
| lauric acid | 11020 | 917 | 15817 | 616 | 1.85 | ＜0.01 | ＜0.01 | 0.70 |
| LysoPC (20:0/0:0) | 1475298 | 89062 | 1843338 | 73065 | 1.13 | ＜0.01 | 0.02 | 0.80 |
| LysoPC (P-18:0) | 3369474 | 190945 | 4153637 | 123248 | 1.10 | ＜0.01 | 0.01 | 0.81 |
| Lyxose | 10342 | 764 | 34286 | 3647 | 2.91 | ＜0.01 | ＜0.01 | 0.30 |
| Myristic Acid | 41418 | 3792 | 48726 | 1613 | 1.00 | ＜0.01 | 0.02 | 0.85 |
| Nicotinic acid | 1857 | 389 | 4155 | 604 | 1.80 | 0.01 | 0.04 | 0.45 |
| Oleic acid | 71344 | 7775 | 108220 | 4342 | 2.16 | ＜0.01 | ＜0.01 | 0.66 |
| O-PHOSPHORYLETHANOLAMINE | 14555 | 1033 | 18905 | 774 | 1.42 | ＜0.01 | 0.01 | 0.77 |
| Ornithine | 1401659 | 66107 | 1184312 | 57622 | 1.19 | ＜0.01 | 0.02 | 1.18 |
| PC (14:0/18:2 (9Z,12Z)) | 847888 | 79686 | 1270283 | 79421 | 1.61 | ＜0.01 | ＜0.01 | 0.67 |
| PC (18:1 (9Z)/18:1 (9Z)) | 1197100998 | 70746350 | 1468007323 | 49770108 | 1.08 | ＜0.01 | 0.02 | 0.82 |
| PC (18:3 (6Z,9Z,12Z)/P-18:1 (11Z)) | 89045141 | 6379249 | 110005732 | 3840936 | 1.22 | ＜0.01 | 0.01 | 0.81 |
| PC (20:3 (5Z,8Z,11Z)/P-18:1 (11Z)) | 77377989 | 4953392 | 93050965 | 2939626 | 1.11 | ＜0.01 | 0.01 | 0.83 |
| Proline | 999293 | 71448 | 638123 | 83726 | 2.89 | ＜0.01 | ＜0.01 | 1.57 |
| SM (d18:0/16:1 (9Z)) | 882443902 | 66959871 | 1161876895 | 42397735 | 1.36 | ＜0.01 | ＜0.01 | 0.76 |
| SM (d18:1/14:0) | 37151501 | 3300648 | 55850403 | 2393231 | 1.95 | ＜0.01 | ＜0.01 | 0.67 |
| SM (d18:1/20:0) | 50438905 | 4966709 | 68534409 | 3098192 | 1.35 | ＜0.01 | 0.01 | 0.74 |
| Stearic acid | 585875 | 75957 | 727709 | 18625 | 1.53 | ＜0.01 | 0.01 | 0.81 |
| Taurine | 294115 | 21280 | 195506 | 8935 | 1.89 | ＜0.01 | ＜0.01 | 1.50 |
| 2-Hydroxybutyric acid | 60738 | 3270 | 44946 | 1497 | 1.47 | ＜0.01 | ＜0.01 | 1.35 |
| L-Threonic acid | 92674 | 6661 | 115875 | 4379 | 1.29 | 0.01 | 0.03 | 0.80 |
| Heptadecanoic acid | 13750 | 1117 | 16348 | 463 | 1.12 | 0.01 | 0.03 | 0.84 |
| Comparison X: Per-AF (n=26) vs. Control (n=87) | | | | | | | | |
| 2-Hydroxyisobutyric acid | 15805 | 1445 | 11578 | 486 | 1.11 | ＜0.01 | 0.01 | 1.37 |
| 2,2-Dimethylsuccinic acid | 18162 | 1953 | 25753 | 931 | 1.28 | ＜0.01 | ＜0.01 | 0.71 |
| 2-Hydroxy-3-methylbutyric acid | 16130 | 1439 | 10292 | 571 | 1.49 | ＜0.01 | ＜0.01 | 1.57 |
| 2-Ketoglutaric Acid | 21185 | 1589 | 11862 | 584 | 1.95 | ＜0.01 | ＜0.01 | 1.79 |
| 2-Pyrrolidone | 133259 | 10259 | 72715 | 4637 | 1.91 | ＜0.01 | ＜0.01 | 1.83 |
| 3-Hydroxyoctanoic acid | 454996 | 87382 | 822641 | 81909 | 1.35 | ＜0.01 | 0.01 | 0.55 |
| 4-Hydroxybenzoic acid | 383070 | 13736 | 306423 | 11527 | 1.02 | ＜0.01 | ＜0.01 | 1.25 |
| 5-Hydroxytryptamine | 8025 | 870 | 12544 | 509 | 1.58 | ＜0.01 | ＜0.01 | 0.64 |
| 6-Keto-prostaglandin F1a | 68834 | 11942 | 111724 | 6273 | 1.75 | ＜0.01 | ＜0.01 | 0.62 |
| Alanine | 7279167 | 293628 | 5149829 | 210430 | 1.47 | ＜0.01 | ＜0.01 | 1.41 |
| CE (20:3 (8Z,11Z,14Z)) | 180824 | 13852 | 363891 | 21293 | 1.82 | ＜0.01 | ＜0.01 | 0.50 |
| Creatine | 745620 | 111309 | 1429802 | 110829 | 1.80 | ＜0.01 | ＜0.01 | 0.52 |
| Creatinine | 2999547 | 211928 | 4700097 | 231509 | 1.22 | ＜0.01 | ＜0.01 | 0.64 |
| Cystine | 219847 | 20333 | 158188 | 6675 | 1.01 | 0.01 | 0.02 | 1.39 |
| Decanoylcarnitine | 10170606 | 430469 | 2601178 | 166348 | 2.69 | ＜0.01 | ＜0.01 | 3.91 |
| Dihydrouracil | 157859 | 21442 | 224070 | 10653 | 1.11 | ＜0.01 | 0.01 | 0.70 |
| dUMP | 150168 | 17198 | 98589 | 5734 | 1.04 | 0.02 | 0.04 | 1.52 |
| Epinephrine | 2455068 | 284876 | 3656048 | 155219 | 1.23 | ＜0.01 | 0.01 | 0.67 |
| Glutamate-3TMS | 997255 | 58013 | 600163 | 18867 | 1.99 | ＜0.01 | ＜0.01 | 1.66 |
| glycerol-3TMS | 250962 | 11121 | 173971 | 4773 | 1.50 | ＜0.01 | ＜0.01 | 1.44 |
| Histamine | 133015 | 26097 | 271824 | 25370 | 1.55 | ＜0.01 | 0.01 | 0.49 |
| Histidine | 634820 | 32971 | 508095 | 27616 | 1.05 | 0.01 | 0.03 | 1.25 |
| Hypoxanthine | 2643028 | 301251 | 4112228 | 262352 | 1.20 | ＜0.01 | 0.01 | 0.64 |
| Isoleucine | 1518313 | 70281 | 1055790 | 29878 | 1.50 | ＜0.01 | ＜0.01 | 1.44 |
| L-Acetylcarnitine | 50022246 | 2672695 | 24049157 | 1521654 | 1.99 | ＜0.01 | ＜0.01 | 2.08 |
| Lactose | 36613 | 3520 | 18375 | 1232 | 2.23 | ＜0.01 | ＜0.01 | 1.99 |
| L-Arginine | 48023684 | 3938205 | 90069678 | 3954779 | 1.83 | ＜0.01 | ＜0.01 | 0.53 |
| L-Asparagine | 64964 | 3649 | 51177 | 2557 | 1.01 | ＜0.01 | 0.01 | 1.27 |
| L-Carnitine | 26434574 | 5318681 | 50520116 | 2979143 | 1.77 | ＜0.01 | ＜0.01 | 0.52 |
| Leucine | 3268090 | 127232 | 2405948 | 67504 | 1.36 | ＜0.01 | ＜0.01 | 1.36 |
| L-Lysine | 1308658 | 86131 | 1808679 | 72552 | 1.05 | ＜0.01 | ＜0.01 | 0.72 |
| L-Serine | 19827 | 2475 | 10313 | 1162 | 1.79 | ＜0.01 | 0.01 | 1.92 |
| L-Threonic acid | 76497 | 9536 | 115875 | 4379 | 1.62 | ＜0.01 | ＜0.01 | 0.66 |
| LysoPC (20:0/0:0) | 1292374 | 100588 | 1843338 | 73065 | 1.37 | ＜0.01 | ＜0.01 | 0.70 |
| LysoPC (P-18:0) | 3029023 | 174389 | 4153637 | 123248 | 1.26 | ＜0.01 | ＜0.01 | 0.73 |
| Lyxose | 11402 | 925 | 34286 | 3647 | 1.92 | ＜0.01 | ＜0.01 | 0.33 |
| Malate-3TMS | 14523 | 815 | 9685 | 315 | 1.61 | ＜0.01 | ＜0.01 | 1.50 |
| Methionine | 254901 | 8754 | 197116 | 5647 | 1.26 | ＜0.01 | ＜0.01 | 1.29 |
| Oleic acid | 62424 | 8698 | 108220 | 4342 | 2.21 | ＜0.01 | ＜0.01 | 0.58 |
| Ornithine | 1736311 | 86438 | 1184312 | 57622 | 1.65 | ＜0.01 | ＜0.01 | 1.47 |
| PC (14:0/18:2 (9Z,12Z)) | 804168 | 58821 | 1270283 | 79421 | 1.25 | ＜0.01 | ＜0.01 | 0.63 |
| PC (16:0/16:0) | 24201001 | 2273593 | 32430680 | 1248491 | 1.08 | ＜0.01 | ＜0.01 | 0.75 |
| PC (18:1 (9Z)/18:1 (9Z)) | 1018187352 | 58697498 | 1468007323 | 49770108 | 1.37 | ＜0.01 | ＜0.01 | 0.69 |
| PC (18:3 (6Z,9Z,12Z)/P-18:1 (11Z)) | 70930215 | 4421439 | 110005732 | 3840936 | 1.60 | ＜0.01 | ＜0.01 | 0.64 |
| PC (18:4 (6Z,9Z,12Z,15Z)/20:0) | 343607010 | 26766558 | 492965270 | 19557225 | 1.24 | ＜0.01 | ＜0.01 | 0.70 |
| PC (20:3 (5Z,8Z,11Z)/P-18:1 (11Z)) | 64576545 | 4045288 | 93050965 | 2939626 | 1.41 | ＜0.01 | ＜0.01 | 0.69 |
| Phenylalanine | 969428 | 35798 | 758047 | 18186 | 1.20 | ＜0.01 | ＜0.01 | 1.28 |
| PI (18:1 (11Z)/20:3 (5Z,8Z,11Z)) | 9272697 | 593585 | 12034437 | 446484 | 1.02 | ＜0.01 | ＜0.01 | 0.77 |
| PI (20:4 (8Z,11Z,14Z,17Z)/18:0) | 2364513 | 167049 | 3371141 | 117359 | 1.37 | 0.00 | ＜0.01 | 0.70 |
| Proline | 1364093 | 189761 | 638123 | 83726 | 2.43 | ＜0.01 | ＜0.01 | 2.14 |
| SM (d18:0/16:1 (9Z)) | 741085981 | 49812356 | 1161876895 | 42397735 | 1.56 | ＜0.01 | ＜0.01 | 0.64 |
| SM (d18:0/20:2 (11Z,14Z)) | 745475 | 45365 | 1020364 | 37330 | 1.07 | ＜0.01 | ＜0.01 | 0.73 |
| SM (d18:1/14:0) | 32754540 | 1840458 | 55850403 | 2393231 | 1.66 | ＜0.01 | ＜0.01 | 0.59 |
| SM (d18:1/20:0) | 38241122 | 3270163 | 68534409 | 3098192 | 1.79 | ＜0.01 | ＜0.01 | 0.56 |
| SM (d18:1/24:1 (15Z)) | 392803956 | 32548871 | 558824606 | 21645057 | 1.22 | ＜0.01 | ＜0.01 | 0.70 |
| Taurine | 309891 | 22961 | 195506 | 8935 | 1.62 | ＜0.01 | ＜0.01 | 1.59 |
| Tyrosine | 1660312 | 56033 | 1228253 | 30421 | 1.41 | ＜0.01 | ＜0.01 | 1.35 |
| Urea-3TMS | 26490 | 2378 | 17337 | 1466 | 1.79 | ＜0.01 | ＜0.01 | 1.53 |
| Uric acid | 1391521 | 160022 | 966334 | 175403 | 1.14 | ＜0.01 | ＜0.01 | 1.44 |
| Valine | 837548 | 30518 | 648037 | 16892 | 1.19 | ＜0.01 | ＜0.01 | 1.29 |
| Comparison XI: Fir-AF (n=22) vs. Par-AF (n=33) | | | | | | | | |
| 2-Hydroxy-3-methylbutyric acid | 14723 | 1116 | 10788 | 790 | 2.02 | ＜0.01 | - | 0.73 |
| 2-Pyrrolidone | 161314 | 13209 | 122485 | 7437 | 1.95 | 0.01 | - | 0.76 |
| Alpha-Tocopherol | 189444 | 8013 | 168150 | 5333 | 1.01 | 0.03 | - | 0.89 |
| Arachidic acid | 53406 | 3858 | 43056 | 2842 | 2.10 | 0.03 | - | 0.81 |
| Asparagine | 94349 | 2719 | 80881 | 2626 | 1.72 | 0.00 | - | 0.86 |
| D-Malic acid | 11831 | 762 | 9713 | 578 | 1.42 | 0.02 | - | 0.82 |
| glycerol | 243540 | 11754 | 213525 | 7109 | 1.58 | 0.03 | - | 0.88 |
| Leucine | 3139096 | 118900 | 2781742 | 94066 | 1.74 | 0.02 | - | 0.89 |
| L-Leucine | 44414 | 7688 | 26164 | 4490 | 2.11 | 0.03 | - | 0.59 |
| Lysine | 740601 | 24712 | 662322 | 20573 | 1.55 | 0.02 | - | 0.89 |
| Lyxose | 13428 | 1272 | 10342 | 764 | 1.49 | 0.01 | - | 0.77 |
| Methionine | 242693 | 8427 | 218887 | 6475 | 1.34 | 0.04 | - | 0.90 |
| Monooleoylglycerol | 1508174 | 72423 | 1988240 | 136486 | 1.55 | 0.01 | - | 1.32 |
| Phenylalanine | 946122 | 35782 | 822665 | 27722 | 1.99 | 0.01 | - | 0.87 |
| SM (d18:1/20:0) | 37244724 | 1977999 | 50438905 | 4966709 | 1.19 | 0.03 | - | 1.35 |
| Tyrosine | 1509851 | 54121 | 1358459 | 48439 | 1.60 | 0.04 | - | 0.90 |
| Comparison XII: Fir-AF (n=22) vs. Per-AF (n=26) | | | | | | | | |
| Citrate | 429426 | 26905 | 556119 | 37966 | 1.71 | 0.01 | - | 1.30 |
| Cysteine | 73235 | 3525 | 89725 | 4865 | 1.29 | 0.02 | - | 1.23 |
| D-Malic acid | 11831 | 762 | 14523 | 815 | 1.97 | 0.02 | - | 1.23 |
| Glucitol | 16045 | 4559 | 25494 | 5316 | 2.30 | 0.02 | - | 1.59 |
| glycolic acid | 13739 | 578 | 16553 | 1004 | 1.19 | 0.03 | - | 1.20 |
| Lactate | 4300636 | 206922 | 4953739 | 215100 | 1.05 | 0.04 | - | 1.15 |
| L-Arginine | 56153820 | 3003144 | 48023684 | 3938205 | 1.14 | 0.04 | - | 0.86 |
| meso-Erythritol | 39604 | 2409 | 45884 | 2319 | 1.56 | 0.04 | - | 1.16 |
| Myristic Acid | 44218 | 4556 | 54706 | 3454 | 1.97 | 0.02 | - | 1.24 |
| PE (22:6 (4Z,7Z,10Z,13Z,16Z,19Z)/P-16:0) | 9431708 | 341807 | 8400963 | 370703 | 1.05 | 0.04 | - | 0.89 |
| PI (18:1 (11Z)/18:1 (11Z)) | 738166 | 34766 | 634088 | 54127 | 1.93 | 0.03 | - | 0.86 |
| Serine | 145630 | 7876 | 124546 | 6238 | 1.39 | 0.04 | - | 0.86 |
| Threonic acid | 98162 | 8297 | 76497 | 9536 | 1.28 | 0.02 | - | 0.78 |
| Comparison XIII: Par-AF (n=33) vs. Per-AF (n=26) | | | | | | | | |
| 2-Hydroxy-3-methylbutyric acid | 10788 | 790 | 16130 | 1439 | 2.02 | ＜0.01 | - | 1.50 |
| 2-Hydroxyisobutyric acid | 11686 | 516 | 15805 | 1445 | 1.43 | 0.03 | - | 1.35 |
| 2-Ketoglutaric Acid | 17254 | 1646 | 21185 | 1589 | 1.75 | 0.02 | - | 1.23 |
| 5-Hydroxytryptamine | 9845 | 702 | 8025 | 870 | 1.95 | 0.01 | - | 0.82 |
| beta-Alanine | 158957 | 8932 | 212814 | 13181 | 1.83 | ＜0.01 | - | 1.34 |
| Beta-Methylglucopyranoside | 874122 | 59110 | 1117685 | 96642 | 1.18 | 0.01 | - | 1.28 |
| Citrate | 400080 | 17717 | 556119 | 37966 | 1.79 | ＜0.01 | - | 1.39 |
| Creatine | 1108026 | 131323 | 745620 | 111309 | 1.90 | 0.02 | - | 0.67 |
| Dihydrouracil | 205581 | 16196 | 157859 | 21442 | 1.50 | 0.02 | - | 0.77 |
| D-Malic acid | 9713 | 578 | 14523 | 815 | 2.30 | ＜0.01 | - | 1.50 |
| glycerol | 213525 | 7109 | 250962 | 11121 | 1.05 | 0.01 | - | 1.18 |
| glycolic acid | 12862 | 584 | 16553 | 1004 | 1.54 | ＜0.01 | - | 1.29 |
| Isoleucine | 1293984 | 42534 | 1518313 | 70281 | 1.08 | 0.02 | - | 1.17 |
| Lactate | 4071387 | 160665 | 4953739 | 215100 | 1.60 | ＜0.01 | - | 1.22 |
| L-Arginine | 63091801 | 5144395 | 48023684 | 3938205 | 1.24 | 0.01 | - | 0.76 |
| Leucine | 2781742 | 94066 | 3268090 | 127232 | 1.27 | ＜0.01 | - | 1.18 |
| L-Glutamic acid | 780240 | 39302 | 997255 | 58013 | 1.83 | ＜0.01 | - | 1.28 |
| meso-Erythritol | 37238 | 1374 | 45884 | 2319 | 1.45 | ＜0.01 | - | 1.23 |
| Myristic Acid | 41418 | 3792 | 54706 | 3454 | 2.01 | ＜0.01 | - | 1.32 |
| Ornithine | 1401659 | 66107 | 1736311 | 86438 | 1.40 | ＜0.01 | - | 1.24 |
| Phenylalanine | 822665 | 27722 | 969428 | 35798 | 1.40 | ＜0.01 | - | 1.18 |
| PI (20:4 (8Z,11Z,14Z,17Z)/18:0) | 2908633 | 132762 | 2364513 | 167049 | 1.26 | 0.01 | - | 0.81 |
| Tyrosine | 1358459 | 48439 | 1660312 | 56033 | 1.24 | ＜0.01 | - | 1.22 |
| Urea | 18749 | 1743 | 26490 | 2378 | 1.28 | 0.01 | - | 1.41 |
| Uric acid | 1440488 | 161294 | 1911058 | 160022 | 2.64 | 0.01 | - | 1.33 |
| Comparison XIV: Fir-AF (n=22) vs. Car-AF (n=32) | | | | | | | | |
| 11Z-Eicosenoic acid | 7771 | 693 | 11977 | 1241 | 1.17 | ＜0.01 | 0.03 | 1.54 |
| 1-Monostearin | 27574 | 3959 | 36879 | 2600 | 1.24 | 0.01 | 0.05 | 1.34 |
| 2,3-Dihydroxybutanoic acid | 12381 | 1423 | 27445 | 3633 | 1.69 | ＜0.01 | 0.01 | 2.22 |
| 2-Hydroxybutyric acid | 55270 | 4511 | 112059 | 11718 | 1.76 | ＜0.01 | ＜0.01 | 2.03 |
| 2-Ketoglutaric Acid | 19473 | 2235 | 26136 | 1863 | 1.06 | 0.01 | 0.05 | 1.34 |
| 3-Hydroxybutyric acid | 164650 | 34298 | 1417573 | 353008 | 3.13 | ＜0.01 | ＜0.01 | 8.61 |
| 6-Keto-prostaglandin F1a | 72841 | 8676 | 108573 | 9707 | 1.29 | 0.01 | 0.04 | 1.49 |
| 9-Hexadecenoic acid | 38734 | 4693 | 74516 | 6642 | 1.78 | ＜0.01 | ＜0.01 | 1.92 |
| Aminomalonic acid | 180830 | 16928 | 132310 | 14868 | 1.23 | 0.01 | 0.04 | 0.73 |
| Asymmetric dimethylarginine | 1487384 | 100781 | 2196622 | 183032 | 1.18 | ＜0.01 | 0.01 | 1.48 |
| Buprenorphine | 12144338 | 1200898 | 8234608 | 640297 | 1.31 | ＜0.01 | 0.03 | 0.68 |
| Citrate | 429426 | 26905 | 681946 | 49588 | 1.57 | ＜0.01 | 0.00 | 1.59 |
| D-Malic acid | 11831 | 762 | 16085 | 933 | 1.20 | ＜0.01 | 0.01 | 1.36 |
| dUMP | 116712 | 14262 | 67903 | 6429 | 1.48 | ＜0.01 | 0.01 | 0.58 |
| Glucitol | 16045 | 4559 | 914725 | 557929 | 3.11 | ＜0.01 | ＜0.01 | 57.01 |
| Glucose 6-phosphate | 163607 | 14443 | 111160 | 10224 | 1.28 | ＜0.01 | 0.02 | 0.68 |
| Glycerol-2-phosphate | 48981 | 3542 | 36651 | 3840 | 1.26 | 0.01 | 0.04 | 0.75 |
| Glycerol-3-phosphate | 47986 | 3585 | 35470 | 3469 | 1.26 | 0.01 | 0.04 | 0.74 |
| homocysteine | 861 | 177 | 14904 | 4011 | 3.33 | ＜0.01 | ＜0.01 | 17.30 |
| Lactose | 30573 | 2452 | 54709 | 5311 | 1.56 | ＜0.01 | ＜0.01 | 1.79 |
| L-Cysteine | 306476 | 18551 | 439056 | 41697 | 1.05 | 0.01 | 0.03 | 1.43 |
| LysoPC (20:0/0:0) | 1426940 | 113262 | 959739 | 74933 | 1.34 | ＜0.01 | 0.01 | 0.67 |
| LysoPC (20:2 (11Z,14Z)) | 8167585 | 665378 | 6249677 | 446032 | 1.06 | 0.01 | 0.03 | 0.77 |
| meso-Erythritol | 39604 | 2409 | 56892 | 3812 | 1.32 | ＜0.01 | ＜0.01 | 1.44 |
| Methyl galactoside | 88817 | 7824 | 130888 | 10956 | 1.24 | ＜0.01 | 0.01 | 1.47 |
| Monooleoylglycerol | 1508174 | 72423 | 2238447 | 158398 | 1.21 | ＜0.01 | ＜0.01 | 1.48 |
| Myo-Inositol | 180052 | 12593 | 258305 | 14424 | 1.41 | ＜0.01 | ＜0.01 | 1.43 |
| Oleic acid | 58973 | 7228 | 98182 | 10818 | 1.56 | ＜0.01 | 0.03 | 1.66 |
| O-PHOSPHORYLETHANOLAMINE | 14238 | 1007 | 10944 | 938 | 1.12 | 0.01 | 0.05 | 0.77 |
| Oxalic acid | 1825186 | 116403 | 1446467 | 99804 | 1.02 | 0.01 | 0.05 | 0.79 |
| PC (16:0/16:0) | 23388309 | 1774908 | 32661644 | 2001274 | 1.24 | ＜0.01 | 0.01 | 1.40 |
| Pyruvate | 162948 | 20705 | 249499 | 25587 | 1.28 | 0.01 | 0.05 | 1.53 |
| Ribitol | 519994 | 45825 | 933829 | 81470 | 1.76 | ＜0.01 | ＜0.01 | 1.80 |
| SM (d18:0/20:2 (11Z,14Z)) | 787938 | 44666 | 1159992 | 85912 | 1.29 | ＜0.01 | ＜0.01 | 1.47 |
| Uridine | 21959 | 1366 | 16176 | 931 | 1.25 | ＜0.01 | 0.01 | 0.74 |
| Comparison XV: Par-AF (n=33) vs. Car-AF (n=32) | | | | | | | | |
| 11Z-Eicosenoic acid | 8218 | 637 | 11977 | 1241 | 1.11 | 0.01 | 0.02 | 1.46 |
| 2-Ketoglutaric Acid | 17254 | 1646 | 26136 | 1863 | 1.35 | ＜0.01 | ＜0.01 | 1.51 |
| 3-Hydroxybutyric acid | 177384 | 43095 | 1417573 | 353008 | 3.30 | ＜0.01 | ＜0.01 | 7.99 |
| 5-Hydroxytryptamine | 9845 | 702 | 7654 | 780 | 1.14 | ＜0.01 | 0.02 | 0.78 |
| 6-Keto-prostaglandin F1a | 68419 | 8315 | 108573 | 9707 | 1.46 | ＜0.01 | ＜0.01 | 1.59 |
| Aminomalonic acid | 180976 | 11062 | 132310 | 14868 | 1.32 | ＜0.01 | 0.01 | 0.73 |
| L-Aspartic acid | 8002 | 589 | 10791 | 620 | 1.21 | ＜0.01 | 0.01 | 1.35 |
| Arachidonic acid | 1594473 | 85236 | 2134515 | 119405 | 1.16 | ＜0.01 | ＜0.01 | 1.34 |
| Beta-Methylglucopyranoside | 874122 | 59110 | 1164177 | 75379 | 1.06 | ＜0.01 | ＜0.01 | 1.33 |
| Buprenorphine | 12650580 | 1082222 | 8234608 | 640297 | 1.17 | ＜0.01 | 0.01 | 0.65 |
| 9-Hexadecenoic acid | 35536 | 2459 | 74516 | 6642 | 2.05 | ＜0.01 | ＜0.01 | 2.10 |
| Citrate | 400080 | 17717 | 681946 | 49588 | 1.84 | ＜0.01 | ＜0.01 | 1.70 |
| Decanoylcarnitine | 2413362 | 318036 | 4075914 | 691404 | 1.42 | ＜0.01 | 0.02 | 1.69 |
| dUMP | 132705 | 14369 | 67903 | 6429 | 1.53 | ＜0.01 | ＜0.01 | 0.51 |
| Glucitol | 23095 | 7873 | 914725 | 557929 | 2.99 | ＜0.01 | ＜0.01 | 39.61 |
| Glucose 6-phosphate | 165851 | 12063 | 111160 | 10224 | 1.47 | ＜0.01 | ＜0.01 | 0.67 |
| homocysteine | 1008 | 137 | 14904 | 4011 | 3.67 | ＜0.01 | ＜0.01 | 14.78 |
| Lactose | 26449 | 1881 | 54709 | 5311 | 1.85 | ＜0.01 | ＜0.01 | 2.07 |
| Linoleic acid | 296515 | 20363 | 414202 | 23513 | 1.38 | ＜0.01 | ＜0.01 | 1.40 |
| LysoPC (20:0/0:0) | 1475298 | 89062 | 959739 | 74933 | 1.45 | ＜0.01 | ＜0.01 | 0.65 |
| D-Malic acid | 9713 | 578 | 16085 | 933 | 1.80 | ＜0.01 | ＜0.01 | 1.66 |
| Myo-Inositol | 172290 | 5577 | 258305 | 14424 | 1.63 | ＜0.01 | ＜0.01 | 1.50 |
| Myo-Inositol-2-phosphate | 41132 | 4853 | 56012 | 3930 | 1.26 | ＜0.01 | 0.02 | 1.36 |
| Myristic Acid | 41418 | 3792 | 54228 | 3678 | 1.03 | 0.01 | 0.03 | 1.31 |
| Niacinamide | 1019951 | 94915 | 637360 | 85897 | 1.73 | ＜0.01 | ＜0.01 | 0.62 |
| Oleic acid | 569782 | 43543 | 927344 | 56452 | 1.78 | ＜0.01 | ＜0.01 | 1.63 |
| Pyruvate | 160822 | 14763 | 249499 | 25587 | 1.36 | ＜0.01 | 0.02 | 1.55 |
| Ribitol | 470679 | 32881 | 933829 | 81470 | 2.04 | ＜0.01 | ＜0.01 | 1.98 |
| SM (d18:0/20:2 (11Z,14Z)) | 850693 | 51716 | 1159992 | 85912 | 1.13 | ＜0.01 | 0.01 | 1.36 |
| 2-Hydroxybutyric acid | 60738 | 3270 | 112059 | 11718 | 1.70 | ＜0.01 | ＜0.01 | 1.84 |
| 2-Hydroxy-3-methylbutyric acid | 10788 | 790 | 20765 | 3030 | 1.21 | ＜0.01 | 0.01 | 1.92 |
| 2,3-Dihydroxybutanoic acid | 11263 | 842 | 27445 | 3633 | 2.05 | ＜0.01 | ＜0.01 | 2.44 |
| meso-Erythritol | 37238 | 1374 | 56892 | 3812 | 1.37 | ＜0.01 | ＜0.01 | 1.53 |
| D-Trehalose | 225762 | 12074 | 311268 | 20275 | 1.19 | ＜0.01 | ＜0.01 | 1.38 |
| Methyl galactoside | 82703 | 4583 | 130888 | 10956 | 1.49 | ＜0.01 | ＜0.01 | 1.58 |
| Uridine | 20453 | 876 | 16176 | 931 | 1.03 | ＜0.01 | 0.01 | 0.79 |
| Comparison XVI: Per-AF (n=26) vs. Car-AF (n=32) | | | | | | | | |
| 2,3-Dihydroxybutanoic acid | 11714 | 977 | 27445 | 3633 | 1.67 | ＜0.01 | ＜0.01 | 2.34 |
| 2-Hydroxybutyric acid | 59714 | 5539 | 112059 | 11718 | 1.77 | ＜0.01 | ＜0.01 | 1.88 |
| 3-Hydroxybutyric acid | 331185 | 145426 | 1417573 | 353008 | 2.98 | ＜0.01 | ＜0.01 | 4.28 |
| 3-Hydroxypyridine | 98661 | 3541 | 122350 | 4253 | 1.03 | ＜0.01 | ＜0.01 | 1.24 |
| 6-Keto-prostaglandin F1a | 68834 | 11942 | 108573 | 9707 | 1.66 | ＜0.01 | 0.01 | 1.58 |
| 9-Hexadecenoic acid | 49210 | 6723 | 74516 | 6642 | 1.38 | ＜0.01 | 0.02 | 1.51 |
| Aminomalonic acid | 198288 | 15928 | 132310 | 14868 | 1.63 | ＜0.01 | ＜0.01 | 0.67 |
| [L-Aspartic acid](https://hmdb.ca/metabolites/HMDB0000191) | 23158 | 1910 | 31286 | 1998 | 1.22 | ＜0.01 | 0.01 | 1.35 |
| Asymmetric dimethylarginine | 1528147 | 131166 | 2196622 | 183032 | 1.24 | ＜0.01 | 0.01 | 1.44 |
| Buprenorphine | 13720993 | 1926624 | 8234608 | 640297 | 1.34 | ＜0.01 | 0.01 | 0.60 |
| dUMP | 150168 | 17198 | 67903 | 6429 | 1.77 | ＜0.01 | ＜0.01 | 0.45 |
| Glucitol | 25494 | 5316 | 914725 | 557929 | 2.41 | ＜0.01 | 0.01 | 35.88 |
| Glucose 6-phosphate | 168205 | 12988 | 111160 | 10224 | 1.51 | ＜0.01 | 0.01 | 0.66 |
| Glycerol-2-phosphate | 54638 | 3491 | 36651 | 3840 | 1.54 | ＜0.01 | ＜0.01 | 0.67 |
| Glycerol-3-phosphate | 53275 | 3347 | 35470 | 3469 | 1.57 | ＜0.01 | ＜0.01 | 0.67 |
| glycolic acid | 16553 | 1004 | 12227 | 548 | 1.23 | ＜0.01 | ＜0.01 | 0.74 |
| homocysteine | 1015 | 170 | 14904 | 4011 | 3.53 | ＜0.01 | ＜0.01 | 14.69 |
| L-Glutamic acid | 1991271 | 82782 | 1520324 | 103307 | 1.17 | ＜0.01 | ＜0.01 | 0.76 |
| Methyl galactoside | 93235 | 8568 | 130888 | 10956 | 1.10 | ＜0.01 | 0.01 | 1.40 |
| Myo-Inositol | 192344 | 9936 | 258305 | 14424 | 1.13 | ＜0.01 | 0.01 | 1.34 |
| Myo-Inositol-2-phosphate | 40088 | 5596 | 56012 | 3930 | 1.25 | ＜0.01 | 0.02 | 1.40 |
| Niacinamide | 1004378 | 116835 | 637360 | 85897 | 1.95 | ＜0.01 | 0.02 | 0.63 |
| Oleic acid | 62424 | 8698 | 98182 | 10818 | 1.59 | 0.01 | 0.04 | 1.57 |
| O-PHOSPHORYLETHANOLAMINE | 16490 | 984 | 10944 | 938 | 1.55 | ＜0.01 | ＜0.01 | 0.66 |
| Oxalic acid | 2015432 | 117444 | 1446467 | 99804 | 1.22 | ＜0.01 | 0.01 | 0.72 |
| PC (16:0/16:0) | 24201001 | 2273593 | 32661644 | 2001274 | 1.17 | ＜0.01 | 0.01 | 1.35 |
| PC (18:4 (6Z,9Z,12Z,15Z)/20:0) | 343607010 | 26766558 | 452952626 | 22321794 | 1.20 | ＜0.01 | 0.01 | 1.32 |
| Ribitol | 559239 | 65211 | 933829 | 81470 | 1.67 | ＜0.01 | ＜0.01 | 1.67 |
| SM (d18:0/20:2 (11Z,14Z)) | 745475 | 45365 | 1159992 | 85912 | 1.51 | ＜0.01 | ＜0.01 | 1.56 |
| Urea | 26490 | 2378 | 17845 | 2705 | 1.67 | ＜0.01 | 0.01 | 0.67 |
| Uridine | 22078 | 1471 | 16176 | 931 | 1.22 | ＜0.01 | 0.01 | 0.73 |
