# Supplementary material for: Comprehensive metabolomic characterization of atrial fibrillation
Source: Front Cardiovasc Med. 2022 Aug 8;9:911845. doi: 10.3389/fcvm.2022.911845 (PMC9393302; doi:10.3389/fcvm.2022.911845)
Supplement: Supplementary file 9 [file Table_9.DOCX]

**Supplemental Table 9.** Differential metabolites and the diagnostic potential for each comparison in the discovery phase

| Differential metabolites | AUROC | 95%CI | | Sensitivity  (%) | Specificity  (%) | LogOR | 95%CI | |
| --- | --- | --- | --- | --- | --- | --- | --- | --- |
|  |  | Lower | Upper |  |  |  | Lower | Upper |
| Comparison I: CP (n=143) vs. Control (n=86) | | | | | | | | |
| Lactate | 0.8485 | 0.7967 | 0.9003 | 0.7902 | 0.8023 | 5.85 | 4.24 | 7.45 |
| D-Glutamic acid | 0.7941 | 0.7351 | 0.8531 | 0.7483 | 0.7442 | 3.93 | 2.72 | 5.13 |
| Glycerol-3-phosphate | 0.7774 | 0.7166 | 0.8383 | 0.7093 | 0.7622 | -2.75 | -3.62 | -1.87 |
| Decanoylcarnitine | 0.8534 | 0.8008 | 0.9060 | 0.8605 | 0.7692 | 2.05 | 1.49 | 2.6 |
| Lactate, glycerol-3-phosphate, and decanoylcarnitine | 0.9430 | 0.9140 | 0.9720 | 0.8880 | 0.9070 | 2.73 | 2.13 | 3.33 |
| Comparison II: All-AFs plus Car-AF (n=113) vs. Sus- AF (n=30) | | | | | | | | |
| Cysteine | 0.8711 | 0.7980 | 0.9442 | 76.67% | 84.07% | -5.97 | -8.14 | -3.80 |
| Proline | 0.8327 | 0.7364 | 0.9291 | 86.73% | 80.00% | 1.43 | 0.80 | 2.05 |
| 2-Pyrrolidone | 0.9038 | 0.8355 | 0.9722 | 86.73% | 90.00% | 3.22 | 2.15 | 4.29 |
| Propionylcarnitine | 0.8870 | 0.8265 | 0.9476 | 76.67% | 88.50% | 3.75 | 2.30 | 5.21 |
| Cystine | 0.8705 | 0.8052 | 0.9358 | 69.91% | 93.33% | 2.61 | 1.57 | 3.65 |
| Cysteine, proline, and 2-pyrrolidone | 0.9737 | 0.9528 | 0.9947 | 89.38% | 100.00% | 2.77 | 1.92 | 3.62 |
| Comparison III: Sus-AF (n=30) vs. Control (n=87) | | | | | | | | |
| Glyceric acid | 0.8314 | 0.7431 | 0.9197 | 83.72% | 76.67% | -4.51 | -6.47 | -2.56 |
| Lyxose | 0.8492 | 0.7781 | 0.9203 | 54.65% | 100.00% | -2.60 | -3.80 | -1.41 |
| [O-phosphorylethanolamine](javascript:;) | 0.9027 | 0.8458 | 0.9596 | 90.70% | 80.00% | -5.30 | -7.39 | -3.21 |
| Glycerol-2-phosphate | 0.8663 | 0.8024 | 0.9301 | 63.95% | 100.00% | -3.67 | -5.14 | -2.21 |
| Glycerol-3-phosphate | 0.8860 | 0.8275 | 0.9445 | 75.58% | 93.33% | -4.58 | -6.39 | -2.78 |
| L-Glutamic acid | 0.8822 | 0.8213 | 0.9430 | 83.72% | 67.05% | -4.73 | -6.72 | -2.75 |
| Glyceric acid, lyxose, and O-phosphorylethanolamine | 0.9616 | 0.9320 | 0.9912 | 100.00% | 81.40% | 2.82 | 1.88 | 3.76 |
| Comparison IV: All-AFs (n=81) vs. Control (n=87) | | | | | | | | |
| Isoleucine | 0.8056 | 0.7394 | 0.8719 | 86.42% | 69.77% | 4.89 | 3.22 | 6.56 |
| Glycerol | 0.8076 | 0.7425 | 0.8728 | 80.25% | 73.26% | 5.11 | 3.39 | 6.84 |
| L-Glutamic acid | 0.8358 | 0.7746 | 0.8970 | 90.12% | 67.44% | 4.88 | 3.29 | 6.47 |
| Ornithine | 0.7699 | 0.6974 | 0.8424 | 76.54% | 69.77% | 2.91 | 1.76 | 4.07 |
| Lactose | 0.8283 | 0.7651 | 0.8915 | 85.19% | 70.93% | 2.95 | 1.97 | 3.94 |
| CE (20:3 (8Z,11Z,14Z)) | 0.8140 | 0.7479 | 0.8800 | 86.05% | 65.43% | -2.39 | -3.20 | -1.57 |
| Decanoylcarnitine | 0.9005 | 0.8525 | 0.9486 | 86.05% | 83.95% | 2.83 | 1.98 | 3.69 |
| SM (d18:1/14:0) | 0.8262 | 0.7621 | 0.8902 | 86.05% | 69.14% | -3.29 | -4.37 | -2.21 |
| 2-Pyrrolidone | 0.8250 | 0.7602 | 0.8898 | 86.42% | 72.94% | 2.56 | 1.76 | 3.37 |
| Lactose, decanoylcarnitine, and 2-pyrrolidone | 0.9436 | 0.9088 | 0.9785 | 95.06% | 83.53% | 2.84 | 2.12 | 3.57 |
| Comparison V: Car-AF (n=32) vs. Control (n=87) | | | | | | | | |
| Homocysteine | 0.8840 | 0.8059 | 0.9621 | 65.63% | 100.00% | 1.78 | 1.07 | 2.49 |
| D-Malic acid | 0.8910 | 0.8303 | 0.9516 | 84.38% | 80.23% | 6.32 | 3.83 | 8.80 |
| 2-Ketoglutaric acid | 0.9106 | 0.8542 | 0.9671 | 78.13% | 91.86% | 4.26 | 2.69 | 5.83 |
| Glycerol-3-phosphate | 0.8590 | 0.7771 | 0.9409 | 93.02% | 65.63% | -3.48 | -4.80 | -2.17 |
| Ribitol | 0.9110 | 0.8409 | 0.9811 | 81.25% | 93.02% | 7.16 | 4.33 | 9.99 |
| Lactose | 0.9190 | 0.8652 | 0.9727 | 87.50% | 84.88% | 3.40 | 2.16 | 4.64 |
| 2-Hydroxybutyric acid | 0.8990 | 0.8322 | 0.9658 | 78.13% | 87.21% | 4.93 | 3.01 | 6.85 |
| LysoPC (P-18:0) | 0.8699 | 0.8012 | 0.9386 | 87.21% | 78.13% | -3.88 | -5.44 | -2.32 |
| LysoPC (20:0/0:0) | 0.8924 | 0.8232 | 0.9617 | 87.21% | 78.13% | -4.60 | -6.33 | -2.86 |
| Homocysteine, lactose, and ribitol | 0.9844 | 0.9657 | 1.0000 | 93.75% | 95.00% | 3.36 | 2.17 | 4.55 |
| Comparison VI: Fir-AF (n=22) vs. Control (n=87) | | | | | | | | |
| Isoleucine | 0.8356 | 0.7487 | 0.9226 | 95.45% | 67.44% | 5.76 | 3.08 | 8.45 |
| Glycerol | 0.8414 | 0.7566 | 0.9263 | 90.91% | 73.26% | 6.61 | 3.54 | 9.68 |
| L-Glutamic acid | 0.8605 | 0.7826 | 0.9383 | 81.82% | 76.74% | 5.31 | 2.99 | 7.63 |
| Lactose | 0.8372 | 0.7502 | 0.9242 | 81.82% | 75.58% | 2.67 | 1.38 | 3.95 |
| SM (d18:1/20:0) | 0.8467 | 0.7744 | 0.9191 | 65.12% | 100.00% | -3.42 | -5.02 | -1.82 |
| Decanoylcarnitine | 0.8969 | 0.8369 | 0.9570 | 86.05% | 86.36% | 2.49 | 1.42 | 3.56 |
| SM (d18:1/14:0) | 0.8488 | 0.7657 | 0.9320 | 74.42% | 86.36% | -3.43 | -5.02 | -1.84 |
| Monooleoylglycerol | 0.8346 | 0.7550 | 0.9142 | 73.26% | 90.91% | -4.08 | -6.12 | -2.05 |
| 2-Pyrrolidone | 0.8866 | 0.8227 | 0.9506 | 90.91% | 74.12% | 3.66 | 2.00 | 5.33 |
| L-Glutamic acid, decanoylcarnitine, and 2-pyrrolidone | 0.9540 | 0.9170 | 0.9910 | 95.45% | 84.71% | 2.88 | 1.84 | 3.93 |
| Comparison VII: Per-AF (n=26) vs. Control (n=87) | | | | | | | | |
| Leucine | 0.8332 | 0.7410 | 0.9253 | 84.62% | 74.42% | 6.18 | 3.37 | 8.98 |
| Isoleucine | 0.8475 | 0.7530 | 0.9420 | 84.62% | 77.91% | 5.83 | 3.36 | 8.31 |
| Glycerol | 0.8511 | 0.7548 | 0.9473 | 84.62% | 77.91% | 6.52 | 3.76 | 9.29 |
| 2-Ketoglutaric Acid | 0.8708 | 0.8028 | 0.9387 | 100.00% | 60.47% | 3.69 | 2.14 | 5.24 |
| D-Glutamic acid | 0.9146 | 0.8638 | 0.9654 | 100.00% | 74.42% | 6.46 | 3.88 | 9.04 |
| Ornithine | 0.8506 | 0.7769 | 0.9243 | 96.15% | 66.28% | 3.47 | 1.88 | 5.07 |
| Tyrosine | 0.8524 | 0.7617 | 0.9431 | 92.31% | 72.09% | 6.98 | 4.02 | 9.93 |
| Lactose | 0.8945 | 0.8356 | 0.9533 | 92.31% | 73.26% | 3.33 | 1.91 | 4.75 |
| Decanoylcarnitine | 0.8953 | 0.8374 | 0.9533 | 76.74% | 92.31% | 2.54 | 1.50 | 3.58 |
| SM (d18:1/14:0) | 0.8520 | 0.7791 | 0.9248 | 86.05% | 73.08% | -3.58 | -5.16 | -1.99 |
| L-Glutamic acid, lactose, and decanoylcarnitine | 0.9665 | 0.9357 | 0.9972 | 100.00% | 86.05% | 3.21 | 2.05 | 4.37 |
| Comparison VIII: Fir-AF (n=22) vs. Par-AF (n=33) | | | | | | | | |
| Phenylalanine | 0.7025 | 0.5624 | 0.8425 | 59.09% | 81.82% | -4.61 | -8.32 | -0.90 |
| Asparagine | 0.7466 | 0.6158 | 0.8774 | 77.27% | 72.73% | -6.64 | -11.29 | -2.00 |
| Lyxose | 0.7052 | 0.5684 | 0.8421 | 90.91% | 48.48% | -1.90 | -3.51 | -0.28 |
| 2-Hydroxy-3-methylbutyric acid | 0.7342 | 0.6027 | 0.8656 | 90.91% | 51.52% | -2.37 | -4.03 | -0.71 |
| Monooleoylglycerol | 0.7245 | 0.5910 | 0.8580 | 54.55% | 90.91% | 2.74 | 0.50 | 4.99 |
| Asparagine and 2-hydroxy-3-methylbutyric acid | 0.8237 | 0.7115 | 0.9358 | 87.88% | 68.18% | 2.19 | 1.00 | 3.38 |
| Comparison IX: Fir-AF (n=22) vs. Per-AF (n=26) | | | | | | | | |
| Cysteine | 0.7091 | 0.5586 | 0.8596 | 46.15% | 95.45% | 2.57 | 0.27 | 4.87 |
| Citrate | 0.7203 | 0.5724 | 0.8682 | 84.62% | 59.09% | 2.45 | 0.40 | 4.51 |
| Myristic acid | 0.7028 | 0.5525 | 0.8531 | 65.38% | 72.73% | 1.99 | 0.18 | 3.79 |
| Glucitol | 0.7127 | 0.5624 | 0.8631 | 92.00% | 50.00% | 1.00 | 0.09 | 1.92 |
| Citrate, myristic acid, and glucitol | 0.8273 | 0.7073 | 0.9473 | 92.00% | 63.64% | 2.21 | 0.93 | 3.49 |
| Comparison X: Fir-AF (n=22) vs. Car-AF (n=32) | | | | | | | | |
| 3-Hydroxybutyric acid | 0.8693 | 0.7761 | 0.9626 | 87.50% | 72.73% | 1.69 | 0.74 | 2.64 |
| Homocysteine | 0.8578 | 0.7590 | 0.9567 | 65.63% | 100.00% | 1.28 | 0.56 | 2.01 |
| Citrate | 0.8494 | 0.7422 | 0.9567 | 84.38% | 77.27% | 4.00 | 1.68 | 6.32 |
| Ribitol | 0.8636 | 0.7577 | 0.9696 | 81.25% | 86.36% | 4.29 | 1.86 | 6.72 |
| Glucitol | 0.8409 | 0.7356 | 0.9462 | 65.63% | 95.45% | 1.39 | 0.49 | 2.30 |
| 2-Hydroxybutyric acid | 0.8168 | 0.7069 | 0.9266 | 53.13% | 100.00% | 2.82 | 1.18 | 4.46 |
| SM (d18:0/20:2 (11Z,14Z)) | 0.7983 | 0.6784 | 0.9182 | 65.63% | 90.91% | 3.68 | 1.35 | 6.02 |
| Homocysteine, ribitol, and glucitol | 0.9828 | 0.9565 | 1.0000 | 90.63% | 100.00% | 3.32 | 1.69 | 4.95 |
| Comparison XI: Par-AF (n=33) vs. Car-AF (n=32) | | | | | | | | |
| 3-Hydroxybutyric acid | 0.8864 | 0.8071 | 0.9656 | 84.38% | 81.82% | 1.65 | 0.84 | 2.46 |
| Homocysteine | 0.8400 | 0.7381 | 0.9418 | 65.63% | 96.97% | 1.34 | 0.67 | 2.01 |
| D-Malic acid | 0.8750 | 0.7907 | 0.9593 | 93.75% | 72.73% | 5.50 | 2.90 | 8.11 |
| Citrate | 0.8864 | 0.8019 | 0.9708 | 84.38% | 81.82% | 5.50 | 2.90 | 8.11 |
| Ribitol | 0.9157 | 0.8366 | 0.9949 | 81.25% | 96.97% | 5.40 | 2.79 | 8.00 |
| Arachidonic acid | 0.8475 | 0.7456 | 0.9495 | 81.25% | 87.88% | 6.20 | 2.61 | 9.79 |
| Myo-Inositol | 0.8305 | 0.7267 | 0.9342 | 71.88% | 87.88% | 5.71 | 2.80 | 8.62 |
| Oleic acid | 0.8419 | 0.7431 | 0.9406 | 78.13% | 81.82% | 3.67 | 1.88 | 5.47 |
| D-Trehalose | 0.8333 | 0.7249 | 0.9418 | 71.88% | 90.91% | 5.71 | 2.30 | 9.12 |
| Methyl galactoside | 0.8580 | 0.7644 | 0.9515 | 71.88% | 90.91% | 5.00 | 2.25 | 7.75 |
| Homocysteine, citrate, and ribitol | 0.9602 | 0.9215 | 0.9989 | 96.88% | 78.79% | 2.71 | 1.61 | 3.81 |
| Comparison XII: Per-AF (n=26) vs. Car-AF (n=32) | | | | | | | | |
| Glycolic acid | 0.7885 | 0.6706 | 0.9063 | 88.46% | 62.50% | -4.46 | -7.15 | -1.76 |
| 3-Hydroxybutyric acid | 0.8317 | 0.7233 | 0.9402 | 78.13% | 80.77% | 1.15 | 0.51 | 1.79 |
| Homocysteine | 0.8486 | 0.7504 | 0.9467 | 65.63% | 96.15% | 1.26 | 0.60 | 1.92 |
| Ribitol | 0.8413 | 0.7282 | 0.9545 | 87.50% | 80.77% | 3.12 | 1.35 | 4.89 |
| SM (d18:0/20:2 (11Z,14Z)) | 0.8149 | 0.7040 | 0.9258 | 71.88% | 84.62% | 3.96 | 1.79 | 6.12 |
| Glycolic acid, homocysteine, and SM (d18:0/20:2 (11Z,14Z)) | 0.9567 | 0.9120 | 1.0000 | 90.63% | 84.62% | 2.77 | 1.57 | 3.96 |
