# Supplementary figures and images for: Comprehensive metabolomic characterization of atrial fibrillation
Source: Front Cardiovasc Med. 2022 Aug 8;9:911845. doi: 10.3389/fcvm.2022.911845 (PMC9393302; doi:10.3389/fcvm.2022.911845)

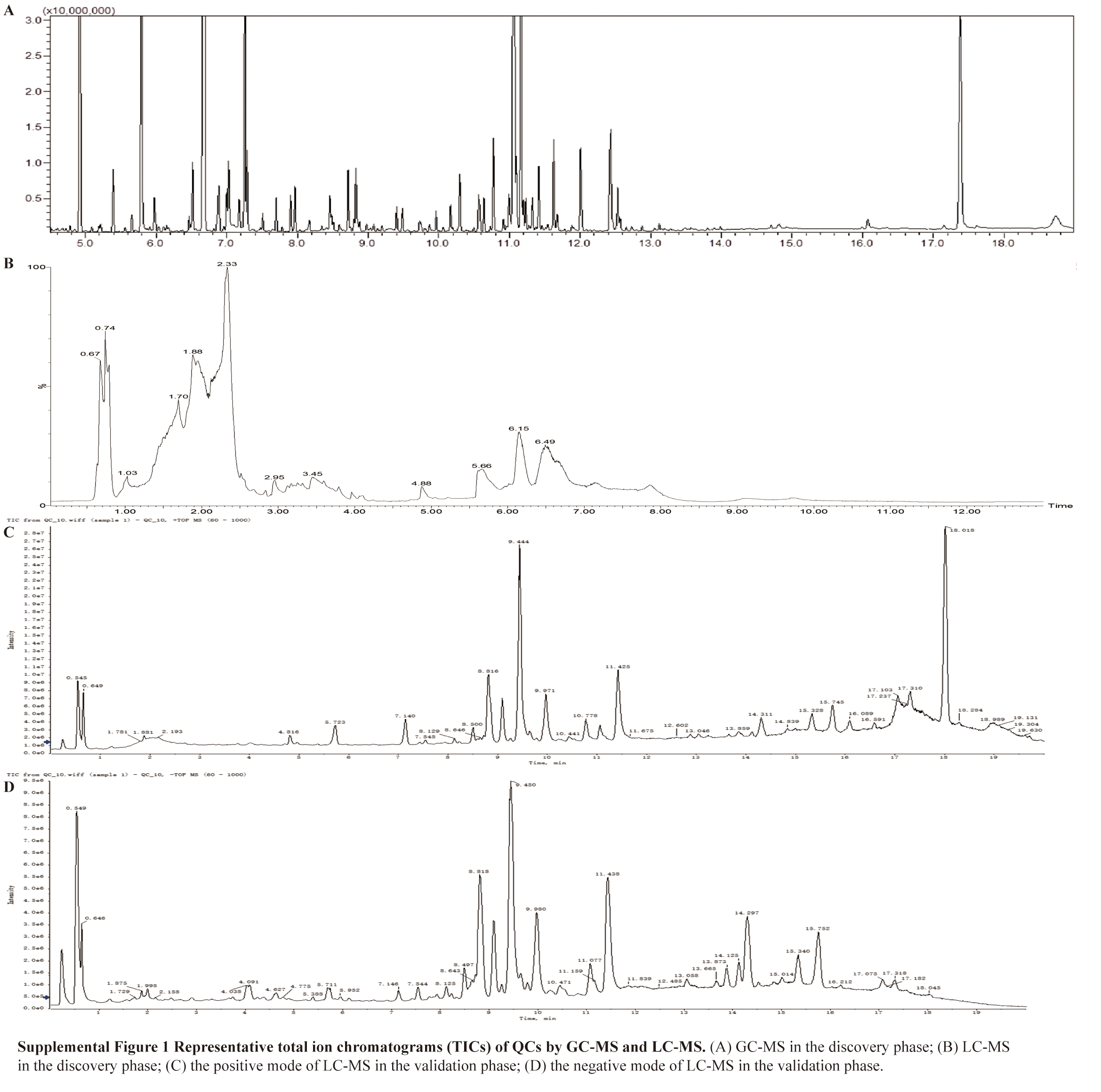

Supplement: Supplementary file 10 [file Image_1.TIF]

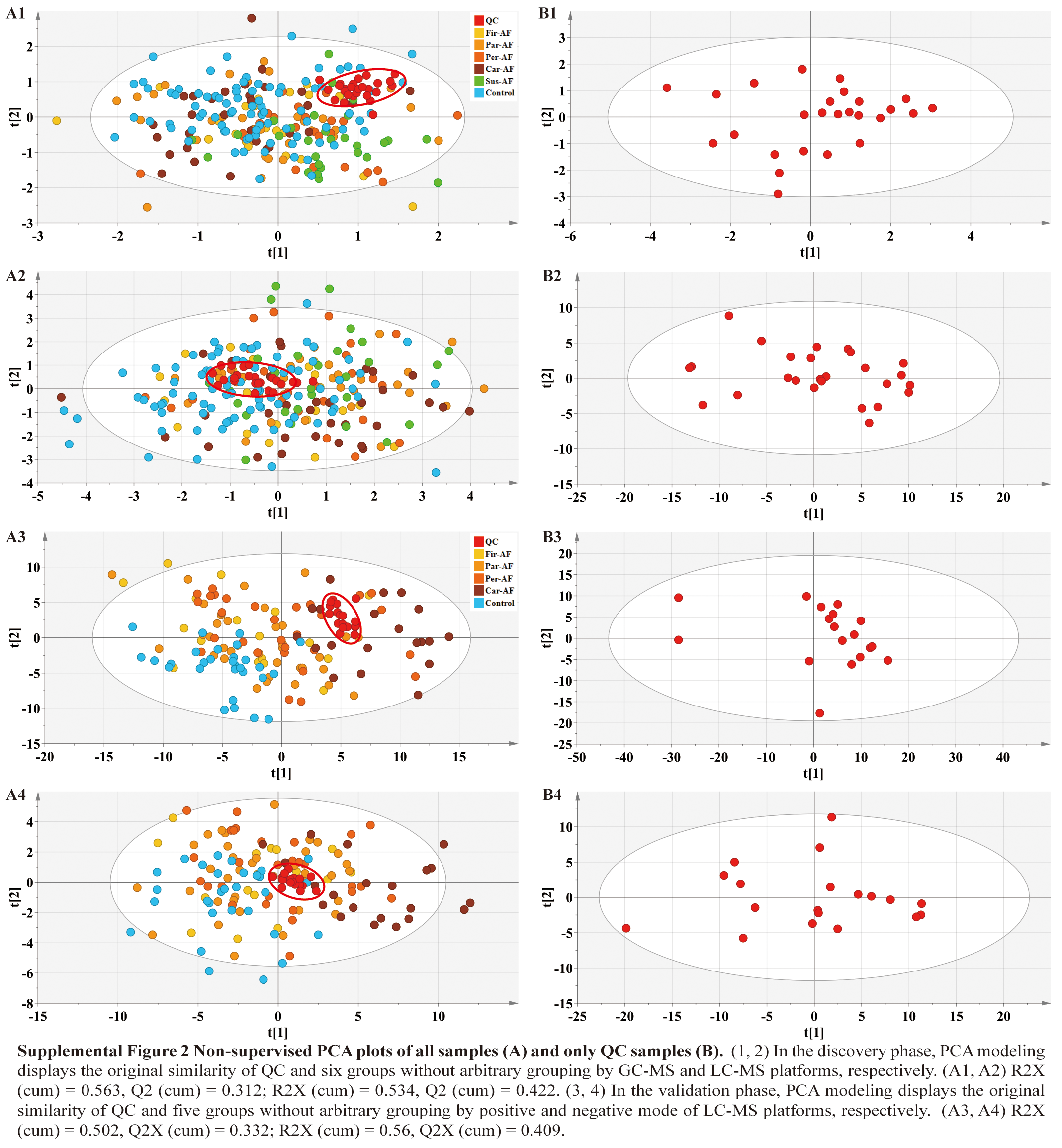

Supplement: Supplementary file 11 [file Image_2.TIF]

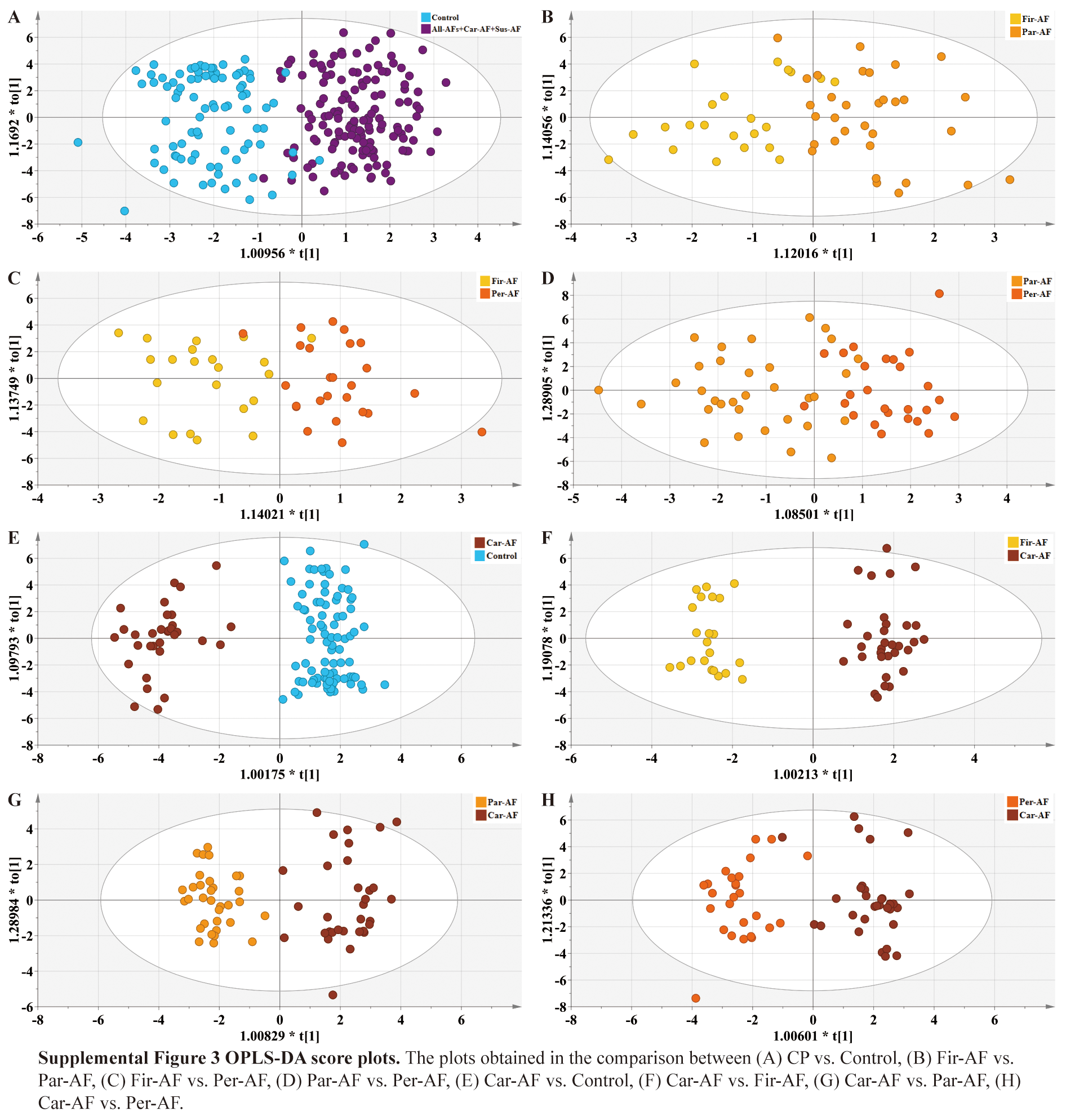

Supplement: Supplementary file 12 [file Image_3.TIF]

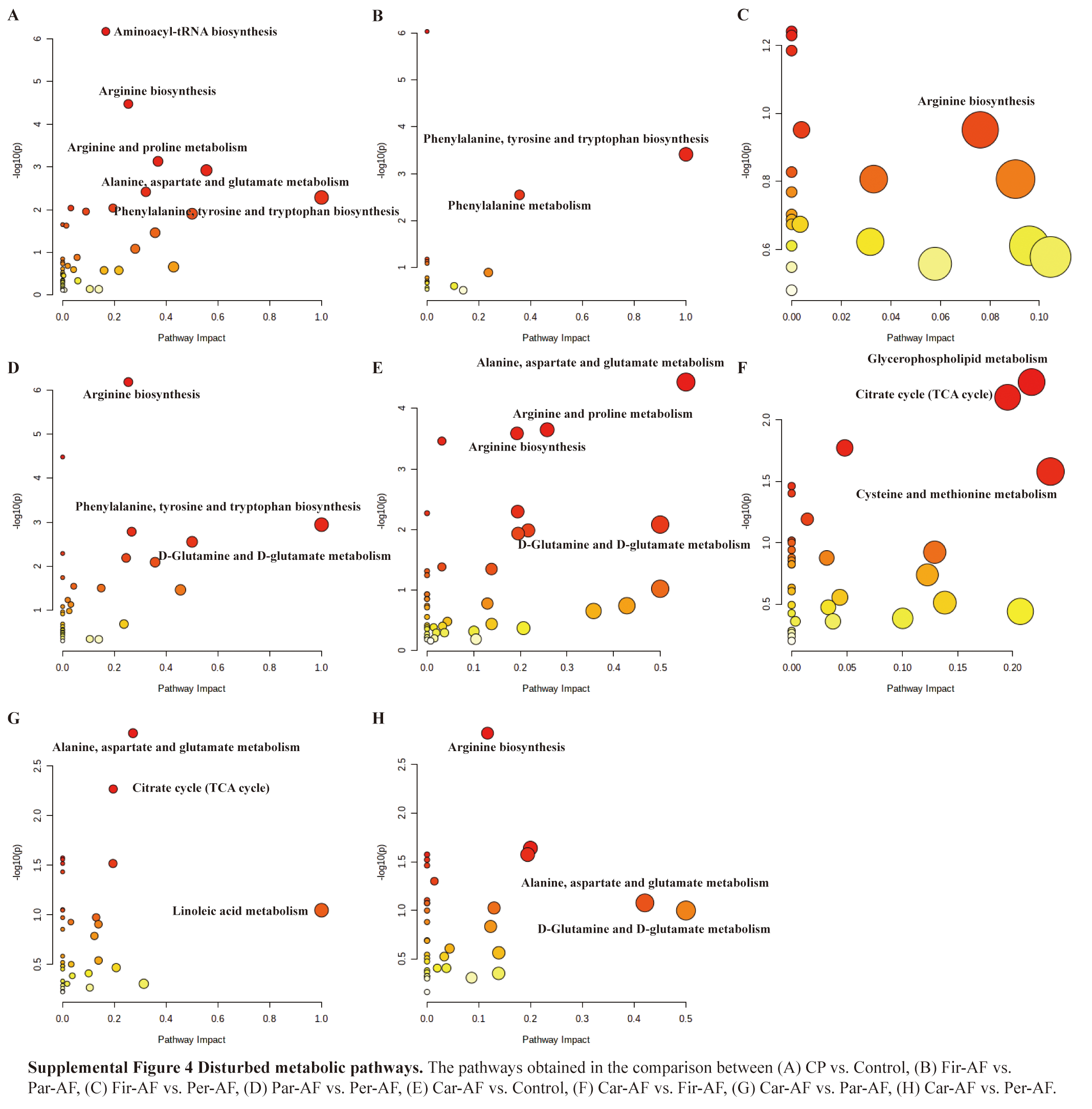

Supplement: Supplementary file 13 [file Image_4.TIF]

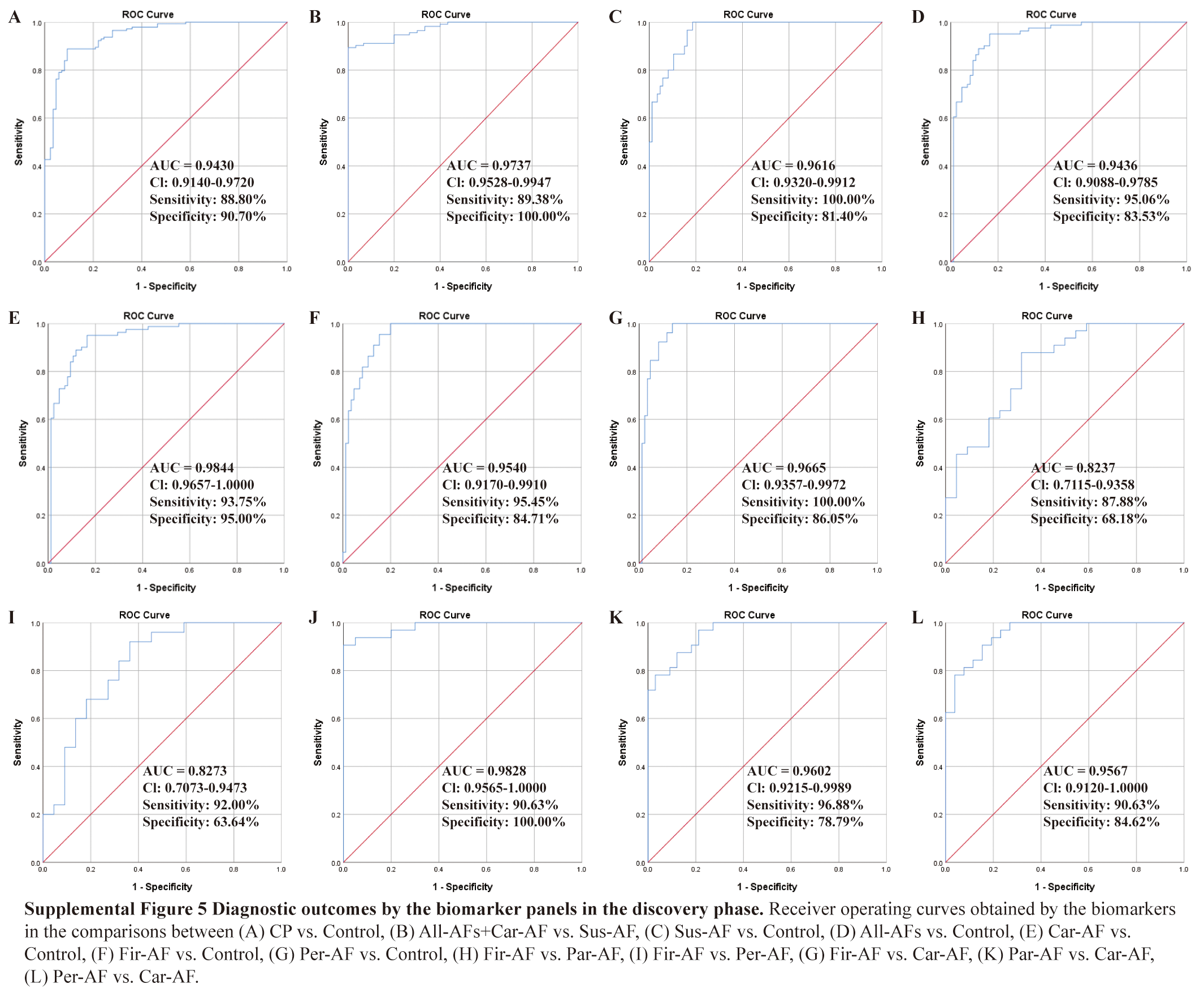

Supplement: Supplementary file 14 [file Image_5.TIF]

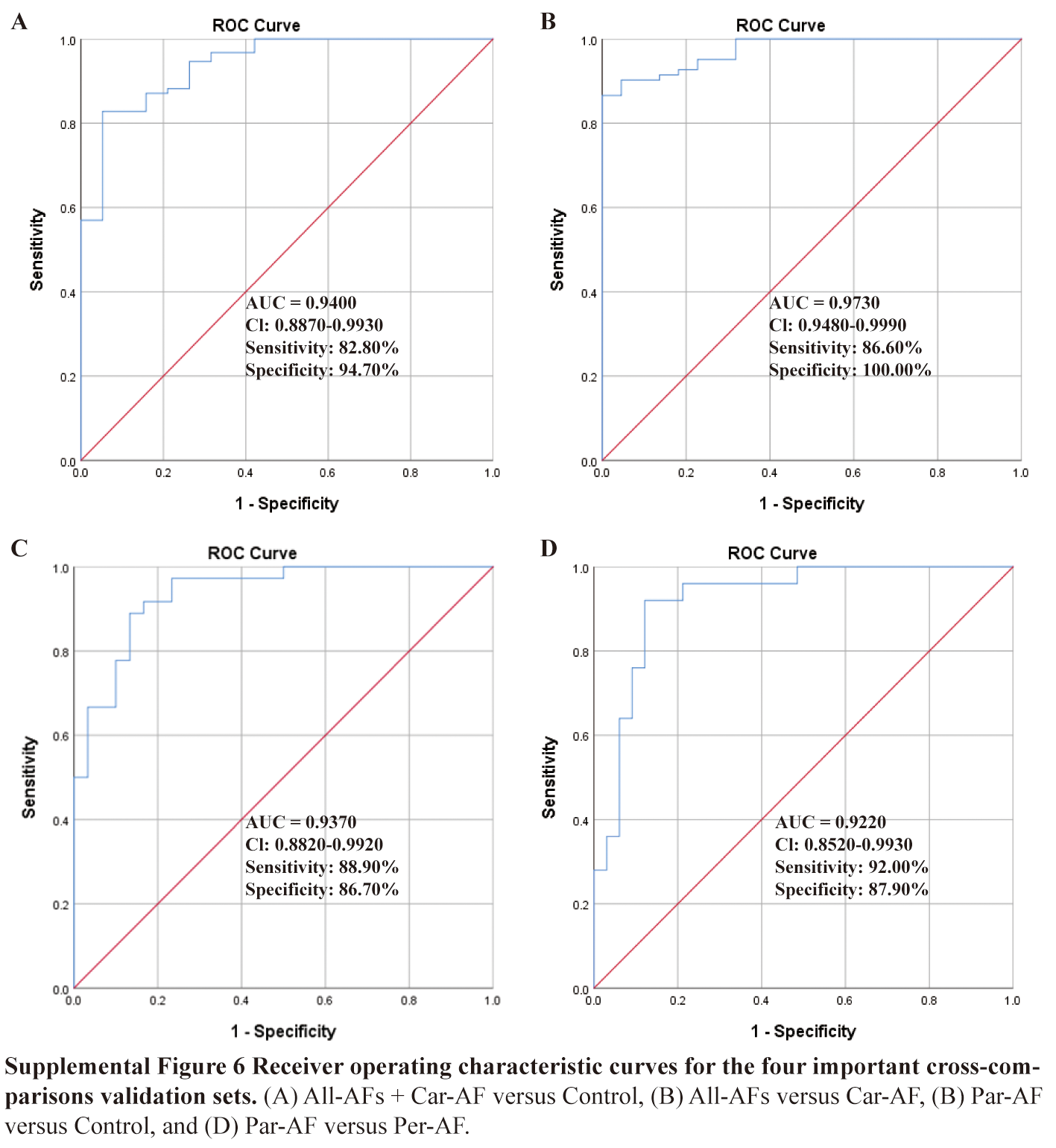

Supplement: Supplementary file 15 [file Image_6.TIF]
